# HES-Mediated Repression of Pten in *Caenorhabditis elegans*

**DOI:** 10.1534/g3.115.019463

**Published:** 2015-10-04

**Authors:** Han Ting Chou, Raymarie Gomez Vazquez, Kun Wang, Richard Campbell, Gaolin Zheng Milledge, Walter W. Walthall, Casonya M. Johnson

**Affiliations:** * Department of Biology, Georgia State University, Atlanta, Georgia 30303; † Center for Obesity Reversal, Georgia State University, Atlanta, Georgia 30303; ‡ Department of Environmental Health Science, Division of Biostatistics, New York University School of Medicine, New York, New York 10016; § Department of Mathematics and Computer Science, North Carolina Central University, Durham, North Carolina 27707

**Keywords:** hairy/enhancer-of-split, dauer recovery, gonad morphology, unfertilized oocytes, gene expression microarray

## Abstract

The hairy/enhancer-of-split (HES) group of transcription factors controls embryonic development, often by acting downstream of the Notch signaling pathway; however, little is known about postembryonic roles of these proteins. In *Caenorhabditis elegans*, the six proteins that make up the REF-1 family are considered to be HES orthologs that act in both Notch-dependent and Notch-independent pathways to regulate embryonic events. To further our understanding of how the REF-1 family works to coordinate postembryonic cellular events, we performed a functional characterization of the REF-1 family member, HLH-25. We show that, after embryogenesis, *hlh-25* expression persists throughout every developmental stage, including dauer, into adulthood. Like animals that carry loss-of-function alleles in genes required for normal cell-cycle progression, the phenotypes of *hlh-25* animals include reduced brood size, unfertilized oocytes, and abnormal gonad morphology. Using gene expression microarray, we show that the HLH-25 transcriptional network correlates with the phenotypes of *hlh-25* animals and that the *C. elegans* Pten ortholog, *daf-18*, is one major hub in the network. Finally, we show that HLH-25 regulates *C. elegans* lifespan and dauer recovery, which correlates with a role in the transcriptional repression of *daf-18* activity. Collectively, these data provide the first genetic evidence that HLH-25 may be a functional ortholog of mammalian HES1, which represses PTEN activity in mice and human cells.

In *Caenorhabditis elegans*, HLH-25 is one of six members of the REF-1 family of basic helix-loop-helix (bHLH) transcription factors (Alper and Kenyon 2001). This family is characterized by the presence of two bHLH domains and are considered functional orthologs of the hairy/enhancer-of-split (HES) family, partly because of their roles as HES-like transducers of Notch signaling in early embryos (Neves and Priess 2005). REF-1, the first family member to be identified via genetic screens, acts downstream of both Notch and GATA signaling to direct endoderm specification (Hermann *et al.* 2000; Neves *et al.* 2007) and to direct neuronal lineage decisions (Lanjuin *et al.* 2006), including the V-ray lineage in *C. elegans* males (Ross *et al.* 2005). In addition to acting downstream of embryonic Notch signaling events, the family member HLH-29 is targeted by LIN-12/Notch during vulva muscle formation in L4 stage animals (Li *et al.* 2013). HLH-29 also acts postembryonically to transcriptionally regulate a diverse set of genes, including those required for chemorepulsive behaviors (McMiller *et al.* 2007), IP3-mediated movement of unfertilized oocytes and fertilized eggs through the spermatheca (White *et al.* 2012), iron homeostasis, and oxidative stress response (Quach *et al.* 2013).

Biological roles have not yet been identified for HLH-25. Notch-dependent, embryonic expression of the gene *hlh-25* occurs in descendants of the AB blastomeres, cells that eventually give rise to the pharynx, the nervous system, and the hypodermis (Neves and Priess 2005; Schnabel and Priess 1997). Notch-independent, embryonic expression of *hlh-25* is mediated by the GATA factor MED-1 and occurs in descendants of the MS blastomere, which also give rise to the pharynx and muscle and to the somatic gonad (Broitman-Maduro *et al.* 2005; Schnabel and Priess 1997). Yeast two-hybrid analysis and protein binding microarrays suggest that the REF-1 family proteins fail to heterodimerize with other bHLH proteins and instead bind E-box-like sequences as homodimers (Grove *et al.* 2009; Powell *et al.* 2004). Of all the REF-1 family proteins, HLH-25 has the greatest degree of DNA-binding promiscuity and tightly binds to five E-box-like sequences. As a comparison, REF-1 binds tightly and HLH-26 binds weakly to the same E-box-like sequence, whereas HLH-29 only binds weakly to two sequences (Grove *et al.* 2009). Gene Ontology (GO) of the genes containing HLH-25 binding sites predicts that HLH-25 plays critical roles in cell division and development, including embryonic development, larval development, growth, and specification of cell fate (Grove *et al.* 2009).

HLH-25’s putative role in regulating cell division and embryonic and postembryonic development supports previous studies suggesting that the *C. elegans* REF-1 family proteins are orthologs of the mammalian HES protein family (Neves *et al.* 2007). Like the other REF-1 family proteins, the sequence similarity between HLH-25 and the HES protein family lies within the bHLH domain. HLH-25 is more similar to HES1 than to the other mouse HES proteins but is most similar to the mouse Atonal homolog protein Math1 when amino acid residues flanking the bHLH domain are included in the analysis. Both Atonal1 and Hes1 play well-established roles in directing neuronal cell fate decisions (Akazawa *et al.* 1995; Ben-Arie *et al.* 2000; Ouji *et al.* 2013; Tomita *et al.* 2000; Wang *et al.* 2001), as do the respective, previously characterized *C. elegans* orthologs LIN-32 and LIN-22 (Miller and Portman 2011; Wrischnik and Kenyon 1997). Other studies suggest that the human and mouse HES1 proteins are oncogenic (Axelson 2004; Gao *et al.* 2014; Sang *et al.* 2010), underscoring the roles that these proteins play in regulating cell division and cell proliferation.

In this study, we describe a functional characterization of HLH-25 by using reverse genetics and molecular approaches. We show that *hlh-25* is expressed postembryonically in body wall muscles and in neurons and that the phenotypes of *hlh-25* animals include reduced brood size, unfertilized oocytes, and abnormal gonad morphology, all of which are consistent with defective cell-cycle or cell division events during embryogenesis. We show that the predicted HLH-25 transcriptional network, based on gene expression microarray, includes the Pten ortholog DAF-18 and other genes that mediate cell division, embryonic development, and larval development. Finally, we show that the ability of HLH-25 to facilitate dauer recovery and lifespan correlates with our mRNA studies suggesting that HLH-25 transcriptionally represses *daf-18*/Pten expression.

## Materials and Methods

### *C. elegans* growth and culture conditions

The following strains were used: N2 Bristol wild-type (Brenner 1974); VC1220, *hlh-25(ok1710)* II; RB712, *daf-18*(ok480) IV; IC748, quIs18; CMJ3001, cmjEx31(P*hlh-25*::GFP::unc-54 ’UTR in pPD95.67 + pRF4); CMJ3002, *hlh-25(ok1710)* II; cmjEx32[(P*hlh-25*::*hlh-25*) + (Pmyo2::mCherry::unc-54 3’UTR)]; and CMJ3003, *hlh-25(ok1710)* II; *daf-18(ok480)*. The extrachromosomal array cmjEx31 includes 1540 nucleotides upstream of the predicted initiation codon, plus the first 30 nucleotides of the coding region for *hlh-25*, fused in-frame to sequences coding green fluorescent protein. The extrachromosomal array cmjEx32 includes the genomic DNA sequences spanning from 1540 nucleotides upstream of the initiator codon to 573 nucleotides downstream of the predicted stop codon for *hlh-25*. VC1220 animals were outcrossed 10 times to N2 animals, and final homozygosity was confirmed by polymerase chain reaction (PCR). Animals were maintained at 20° on nematode growth media (NGM) agar plates seeded with the *Escherichia coli* strain OP50 and were synchronized by alkaline hypochlorite treatment as previously described (Lewis and Fleming 1995). Bacteria-mediated RNA interference was performed as previously described (Kamath *et al.* 2003; Quach *et al.* 2013) via HT115-producing dsRNA for either the control gene *unc-55* or for *hlh-25/hlh-27*. The genes *hlh-25* and *hlh-27* are predicted to produce identical mRNAs and it is impossible to selectively silence *hlh-25*.

### Microscopy

For imaging, animals were anesthetized either with 0.2% levamisole (Govindan *et al.* 2009) or with 10 mM NaN_3_, mounted on 2% agarose pads containing 10 mM NaN_3_, and imaged using either a 40× or a 60× water-immersion objective with the Nikon Eclipse 90i microscope equipped with a Nikon Coolsnap CCD camera. Z projections (sums of intensity) were obtained using NIS-Elements AR, version 4.0. For expression profiling, animals were cultured for at least two generations at 16° and then were synchronized and allowed to grow to the appropriate stage (L1−L4). Forty animals per larval stage were examined for expression. For dauer stage expression, 10 starvation-induced dauer were imaged.

### Egg-laying assays

L4-stage animals were selected to individual NGM plates and allowed to molt to adulthood at 20°. At the start of the egg-laying period, each animal was moved daily to a new plate, already seeded with OP50, until 3 d after eggs or unfertilized oocytes were no longer detected. Eggs and oocytes were counted on the day that the hermaphrodite was removed. The plate was then incubated at 20° for 24 hr, and the numbers of live progeny and unhatched eggs were counted. Eggs that did not hatch within the 24-hr period were scored as dead, whereas the number of live progeny was checked again in the next 24 hr and then removed. For rescue assays, the ratio of unfertilized oocytes to fertilized eggs was calculated daily.

### Lifespan assays

Lifespan assays were performed as previously reported (Quach *et al.* 2013). Lifespan survival comparisons were done individually for each of three biological replicates using the log rank test in GraphPad Prism 6 (GraphPad Software, Inc) and were then repeated with the data from all biological replicates combined into one experiment. Combined data are presented as a graph in Figure 5. Individual data are shown in Table S2.

### Dauer recovery assays

To induce dauer formation, embryos were collected by hypochlorite treatment, washed extensively in water, and then approximately 200 embryos were placed onto an NGM plate containing a sparse lawn of OP50 (∼10 µL of saturated OP50, 24 hr before use). Embryos were allowed to hatch, and the dauer larva to develop, undisturbed at 27° for at least 96 hr. Cultures were visually examined to ensure that at least 90% of the larvae were arrested as dauer. Failure to include OP50 on the plate resulted in animals that arrested as starved L1 stage larvae. For dauer recovery, 25 dauers per strain were individually moved to a plate with a thick lawn of OP50 and incubated at 20°. Dauers were examined every 3 hr during a 24-hr period for fat accumulation, pharyngeal pumping, crescent formation, and then at 8-hr intervals for another 24 hr for oocyte/embryo formation, and onset of egg-laying. The rates of onset of pharyngeal pumping were plotted as survival from dauer, and were compared by log-rank test using GraphPad Prism 6. The timing of dauer recovery varied with the day of the assay, with the onset of pumping occurring in 50% of wild-type animals as early as 3 hr or as late as 8.5 hr. In all assays the trend was the same, and the data shown are representative of one experiment.

### Total RNA isolation, cDNA synthesis, reverse transcription, and quantitative PCR (qPCR)

Total RNA extraction and cDNA synthesis were carried out as previously described (Quach *et al.* 2013) except total RNA was extracted from synchronized L4 stage animals. After cDNA synthesis, qPCR assays were performed with Taqman Gene Expression Assays (Invitrogen) for detection of amplicon, using relative quantitation with normalization against the endogenous control gene *pmp-3* (Hoogewijs *et al.* 2008). Values shown represent the averages of at least three different experiments. The ID numbers for the Taqman Assays used in this study are available upon request.

### Gene expression microarray

Gene expression microarray (GEO accession #GSE65417), including probe preparation, hybridization, fluidics run and chip scan, was performed by Georgia State University DNA/Protein Core Facility. Global gene expression in synchronized populations of VC1220 animals was compared to expression in N2 (wild-type) animals using GeneChip *C. elegans* Genome Array (Affymetrix). Data collection was carried out using GCOS 1.4 software (Affymetrix). Data analysis was performed using GeneSpring *GX 11* Software (Agilent, Palo Alto, CA), and probe intensity values were normalized using robust multichip average algorithm. The quality controls on samples and on probe sets were performed stepwise to detect the outlying samples and the poor probe sets. The principal components analysis score plot and hybridization controls plot were applied for sample detection. Those probe sets passing minimal detection cutoffs and quality control measurements subsequently were used for statistical analysis. The Student’s *t*-test was performed to find the candidates for differential expression, and genes with significant signal level between different conditions (*P* < 0.05) were collected. In addition, fold change analysis were performed on the genes with significant expression, and all genes showing greater than two-fold change were considered putative targets.

### Functional analysis by GO

GO analysis was performed via the Database for Annotation, Visualization and Integrated Discovery (*i.e.*, DAVID), version 6.7, to cluster related target genes (Huang da *et al.* 2009a,b). Additional GO terms and functional information for putative targets were assigned based on Wormbase annotations (Harris *et al.* 2010). Interactions and connectivity between target gene products were identified using the Search Tool for the Retrieval of Interacting Genes/Proteins (Szklarczyk *et al.* 2011) and redrawn manually.

### Mobility assays

For locomotion assays, individual L4 stage animals were placed by platinum wire onto an unseeded NGM plate, allowed to recover for 5 min, and then examined after the start of forward locomotion for 30 sec. For thrashing assays, individual animals were placed into 50 µL of M9 medium. Each lateral movement of the head was counted as one thrash, and movement was monitored for one 30-sec interval per animal. Each biological replica included at least 25 animals per strain, and each assay was repeated for three biological replicas.

### Data availability

The Gene expression microarray (GEO accession #GSE65417) will release for public access on January 1, 2016.

## Results

### Phenotypes of *hlh-25(ok1710)* animals and P*hlh-25*::GFP activity suggest roles for HLH-25 in reproduction

**Figure 1:**
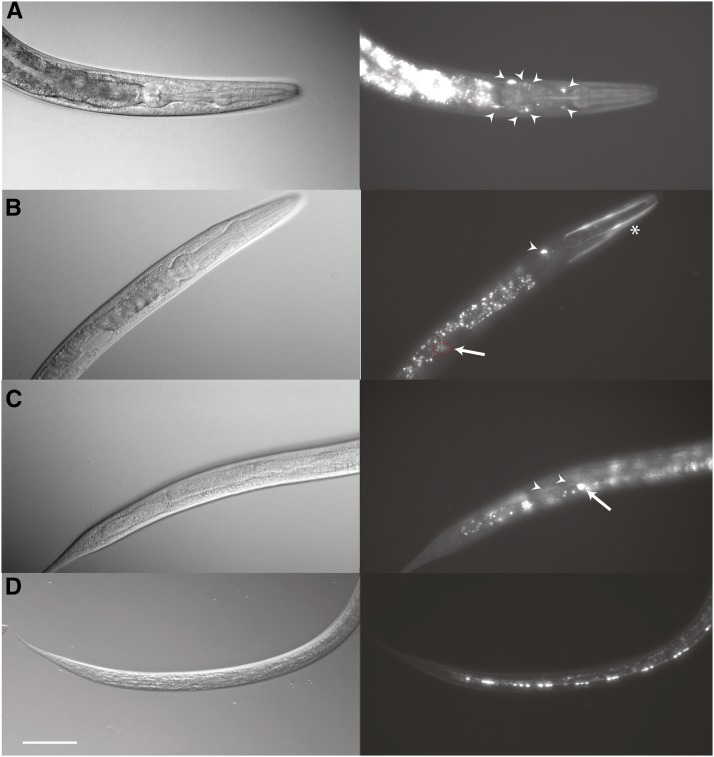
Postembryonic expression of P*hlh-25*::GFP. (A−D) Nomarski images (left) accompanied by green fluorescent protein fluorescent images (right). (A) Expression of *cmjEx31* in multiple head neurons (arrowheads). This pattern was detected in animals at all stages, with a few additional neurons at dauer stage. (B) L3 expression in distal tip cell (arrow, red dashed circle), unidentified head neuron (arrowhead) and head muscles (asterisk). (C) L3 stage expression in PDE (arrow). Arrowheads indicate PDE processes. (D) Dauer stage expression in ventral nerve cord neurons. In all images anterior is to the right, dorsal is up. Scale bar represents 50 µm.

Previous studies indicate that *hlh-25* is expressed in eight-cell stage embryos in response to Notch signalling, and in later stage embryos in response to GATA signalling (Broitman-Maduro *et al.* 2005; Neves and Priess 2005). Using an *hlh-25*::*GFP* transcriptional reporter *cmjEx31* (supporting information, Figure S1), we detected postembryonic expression of *hlh-25* at all larval and adult stages, including dauer stage, in unidentified neurons of the head and tail, the anal depressor muscle, anterior and posterior intestinal cells, and the head and body wall muscles of transgenic animals (Figure 1). We detected expression in the PDE sensory neuron pairs and rare, transient expression in migrating distal tip cells of L3/L4 stage animals. We also detected stage-specific *hlh-25* expression in multiple neurons of the ventral nerve cord in starvation-induced dauer stage animals. We were able to use cell body location and commissure morphology to identify three of the motor neurons as VD1, DA3, and DA7. Expression in the body wall and head muscles and in the PDE neuron pair was not evident in dauer larvae.

**Figure 2:**
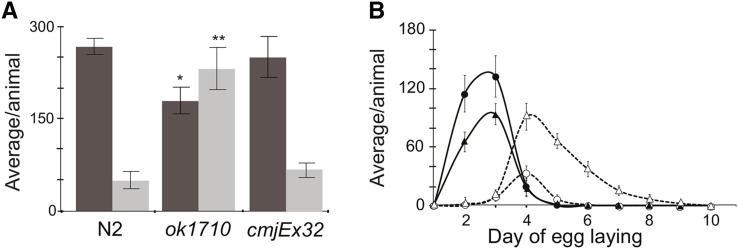
Brood size and unfertilized oocytes *hlh-25(ok1710)* animals. (A) *hlh-25(ok1710)* animals have fewer live progeny and lay more unfertilized eggs than wild-type animals. Expression of the extrachromosomal array cmjEx32, which carries *hlh-25* genomic sequences, rescues this phenotype in *hlh-25(ok1710)* animals. Graph shows mean of total numbers of live progeny (dark bars) and unfertilized eggs (light bars) produced during the entire lifespans of wild-type (n = 7), *hlh-25(ok1710)* (n = 12), and *hlh-25(ok1710*; *cmjEx32)* animals (n = 9). *P-value < 0.05, **P-value < 0.005. (B) Average numbers of live progeny (solid lines) and unfertilized eggs (dashed lines) produced per day of egg laying by the same *hlh-25(ok1710)* animals (triangles) and wild-type animals (circles) as those shown in (A).

The *hlh-25(ok1710)* allele is a null allele that was generated by ethyl methanesulfonate mutagenesis. It is a 1550-bp deletion that spans from 292 bp upstream of the initiator codon to 348 bp downstream of the terminator codon (Figure S1). We outcrossed these animals 10 times, confirmed homozygosity for the *ok1710* allele by PCR, and then examined them for mutant phenotypes. *hlh-25(ok1710)* animals produced fewer live progeny at 20° than wild-type animals (Figure 2A, *P*-value = 0.0027) whereas laying more unfertilized oocytes (*P*-value = 0.00014) throughout the egg-laying period.

Adult hermaphrodites produce more oocytes than sperm and will continue to lay unfertilized eggs for a short period after depleting their sperm supply. Under conditions where food is plentiful, and when cultured at 20°, wild-type hermaphrodites lay eggs over the first 5 d of adulthood (Ward and Carrel 1979; White *et al.* 2012). As previously described, we found that wild-type animals lay an increasing number of unfertilized eggs as the sperm supply diminishes. In our assays, wild-type animals continued to lay a small number of unfertilized eggs for one additional day after the active egg-laying period ended (egg-laying day 6), to produce an average total of 49 unfertilized oocytes (Figure 2B, Table S1). *hlh-25(ok1710)* animals continued to produce unfertilized oocytes for 4 days after the active egg-laying period ended (egg-laying day 9), and produced an average total of 232 unfertilized oocytes. This phenotype has been recently named uno-o (Riesen *et al.* 2014), and is also seen in animals that carry null alleles of the REF-1 family protein HLH-29 (White *et al.* 2012). We were able to partially rescue the uno-o phenotype in *hlh-25(ok1710)* animals by expressing *hlh-25* from an extrachromosomal array, *cmjEx32*, so that transgenic animals produced an average of 38% fewer unfertilized oocytes than *hlh-25(ok1710)* animals (Figure 2A).

**Figure 3:**
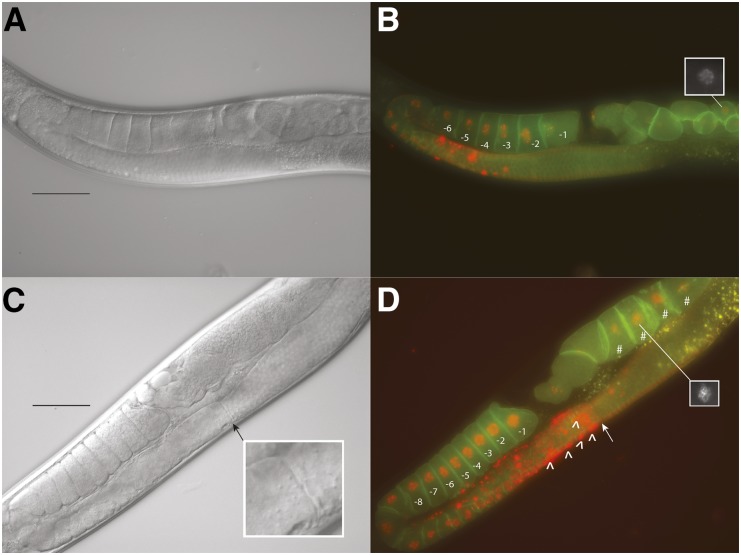
Gonad and oocyte morphologies of *hlh-25(ok1710)* animals. Nomarski (A, C) and merged fluorescent (B, D) images of representative gonad architecture and oocyte morphology in wild-type animals (A, B) as analyzed in a strain coexpressing a fluorescent chromosome marker (mCherry-Histone H2b) and a marker targeting green fluorescent protein to the plasma membrane (PI4,5P_2_) (Green *et al.* 2011), (B). Representative gonad architecture and oocyte morphology in *hlh-25*(*ok1710)* animals (C, D). Phenotypes include increased oocyte number, distal gonad arm constriction (arrow), increased apoptosis (^), and arrested embryos in uterus (#). Insert in (C) shows magnified view of distal gonad arm constriction. Inserts in (B) and (D) show embryonic nuclei. In all images, ventral is up, anterior is to the right, scale bar = 50 µm.

We also examined the gonad arms of adult hermaphrodites. In wild-type animals, oocytes production occurs as progressive movement through two U-shaped gonad arms, starting with the production of germline nuclei at the distal end of each gonad arm, and ending with the ovulation of the oocyte into the sperm-filled spermatheca at the ends proximal to the uterus (Hubbard and Greenstein 2000). In the distal, syncytial region of each arm, the germline nuclei go through mitotic and meiotic divisions while being surrounded by a commonly shared cytoplasmic core. Nuclei start to compartmentalize into oocytes as they move progressively closer to a bend in the gonad arm, and are fully compartmentalized, or budded, as they leave the bend and proceed towards the spermatheca (Green *et al.* 2011). We found that organization of the gonad arm and germline progression were abnormal in 63% of *hlh-25(ok1710)* animals. As shown in Figure 3, and in Figure S2 and Figure S3, gonad architecture was variably abnormal and included constrictions in the distal and proximal gonad arms, increased apoptosis, increased oocyte number, delayed oocyte compartmentalization, abnormal chromatin condensation, and arrested embryos in the uterus.

Other phenotypes of *hlh-25(ok1710)* animals included decreased movement as monitored by thrashing and locomotion assays (Figure S4), leading us to classify *hlh-25* mutants as locomotion variants. This phenotype correlates with the expression of *cmjEx31* in the body wall muscles and in head neurons, but we could not accurately assay for rescue of this phenotype by transgenic expression of *hlh-25* because transgenic animals also carried the *rol-6* marker, which causes a dominant locomotor defect. We did not pursue the locomotion phenotype further in this study.

### DAF-18 is one major hub in the *hlh-25* transcriptional network

We used gene expression microarray to compare the expression profiles of late L4/young-adult stage *hlh-25(ok1710)* animals to profiles of age-matched, wild-type animals. We found that the expression of 630 genes, 506 of which were up-regulated and 124 of which were down-regulated, was altered by at least 2.0-fold (Table S3), and confirmed by reverse transcription (RT)-qPCR that the expression of 21 genes was significantly affected in L4-stage *hlh-25(ok1710)* animals (Table 1). As shown in Table 1, the direction of the change in expression was the same for 18 of the 21 genes. It is possible that the opposite, but significant change in the three remaining genes was due to differences in the developmental age of animals at the times of RNA extraction. Interestingly, 152 of these genes, indicated by asterisks in Table 1 and Table S3, contain predicted HLH-25 binding sites within their putative promoters (Grove *et al.* 2009). Hypergeometric analysis, indicated that this overlap of 152 genes was significantly lower than expected when compared to an overlap between the list of genes identified by protein binding microarray to a randomly chosen gene set of the same size (*P*-value <2.5 × 10^-05^).

**Table 1:** Validation of randomly selected HLH-25 targets by RT-qPCR

WormBase ID	Sequence ID	Gene Name	log_2_ FC (Microarray)	log_2_ FC (RT-qPCR)
WBGene00000230	F27C1.7	*atp-3*	1.14405	1.203510
WBGene00015102	B0280.5	*cpg-2*	1.29866	2.277368
WBGene00000913	T07A9.6	*daf-18**^a^*	1.57550	1.181618
WBGene00001263	K04H4.1	*emb-9*	1.77821	0.688850
WBGene00010305	F59A2.5	F59A2.5*^a^*	−1.17632	−1.630390
WBGene00001394	W02A2.1	*fat-2**^a^*	1.03562	1.718458
WBGene00014095	ZK829.4	*gdh-1**^a^*	2.48027	1.338850
WBGene00001758	Y45G12C.2	*gst-10**^a^*	1.36737	0.774000
WBGene00003022	ZK418.4	*lin-37*	1.58496	1.489140
WBGene00003037	JC8.8	*lin-54*	0.59567	0.425533
WBGene00003230	W02A2.7	*mex-5*	1.04264	0.574521
WBGene00003231	AH6.5	*mex-6*	1.11770	0.656000
WBGene00003242	C37C3.6	*mig-6*	1.71370	0.877350
WBGene00003473	K11G9.6	*mtl-1**^a^*	−4.08804	−1.578000
WBGene00004078	F52E1.1	*pos-1*	1.91456	1.017858
WBGene00004302	K01G5.4	*ran-1**^a^*	1.49570	0.989226
WBGene00004736	K11D9.2	*sca-1*	1.78660	1.270413
WBGene00004984	ZC404.8	*spn-4*	2.06005	0.947934
WBGene00009221	F28F8.2	*acs-2*	−1.71450	1.107159
WBGene00003421	F09E8.3	*msh-5*	1.70929	−1.120290
WBGene00011733	T12D8.5	T12D8.5	−3.24793	0.441060

RT-qPCR, reverse transcription quantitative polymerase chain reaction.

a Target previously predicted by protein binding microarray (Grove *et al.* 2009).

**Figure 4:**
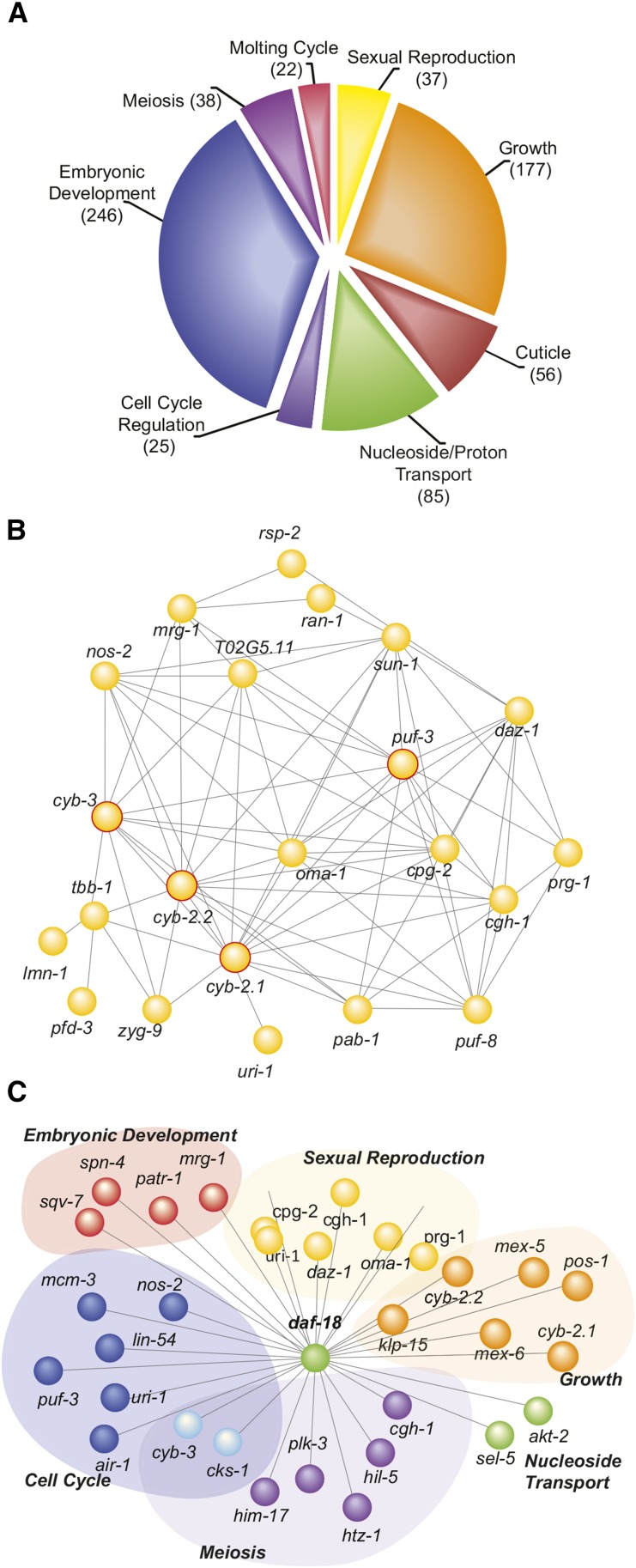
Functional annotation of HLH-25 targets. (A) Distribution of HLH-25 targets into top eight GO clusters. (B) Predicted and known interactions between genes in the ‘sexual reproduction’ cluster. The hub genes *cgh-1*, *cyb-2.1*, cyb-2.2, *cyb-3*, and *puf-3* are outlined in red. The hub gene *daf-18* is not a part of the ‘sexual reproduction’ cluster. (C) Representative genes from the 62 HLH-25 targets that form predicted or known interactions with *daf-18*. All genes, except *daf-18*, clustered under “embryonic development ending in birth (EDB)” Most genes also clustered under at least one other top GO category, including “nucleoside/nucleotide transport” (green spheres).

We clustered related target genes based on enriched GO terms. A total of 328 genes were organized into 10 annotation clusters that all fit within the broad category of “Development” (top eight clusters are shown in Figure 4A). Using the Search Tool for the Retrieval of Interacting Genes/Proteins (STRING 9.0) to identify predicted and known interactions between proteins encoded by the target genes (Szklarczyk *et al.* 2011), we found that the *hlh-25* regulatory network has a high degree of overlap and interconnectivity. For example, 38 genes clustered under the GO term “Sexual Reproduction.” Of those, 22 genes (58%) formed a single, highly interconnected, interaction network (Figure 4B) and also were associated with at least two other clusters. This trend of high connectivity among genes within a cluster and significant overlap among genes between clusters was evident for the entire network. We also identified six genes that we considered to be major hubs (Table 2). These genes were associated with at least four of the GO clusters and formed predicted interactions with at least 35 other targets in the network. Importantly, the hub genes underscore the developmental influence of the *hlh-25* regulatory network, encoding helicases, cyclins, and RNA-binding proteins with established roles in embryogenesis (Table 2). The *C. elegans* homolog of the mammalian *Pten* tumor suppressor, DAF-18 (Masse *et al.* 2005), is one of the major hubs, forming connections with 62 proteins distributed across six annotation clusters (Figure 4C, bold genes in Table S3).

**Table 2:** Hub genes in the HLH-25 network

Wormbase ID	Sequence ID	Gene Name	Functional Summary*^a^*	GO Cluster Association					
				ED	S	P	M	G	C
WBGene00000479	C07H6.5.2	*cgh-1*	ATP-dependent helicase; prevents apoptosis of developing embryos	X	X	X	X	X	
WBGene00000866	Y43E12A.1	*cyb-2.1*	Cyclin B family; required for oocyte maturation	X	X	X		X	X
WBGene00000868	T06E6.2a.2	*cyb-3*	G2/mitotic-specific, Cyclin B3	X	X	X			X
WBGene00000913	T07A9.6	*daf-18*	Phosphatase; negative regulator of insulin-like signaling	X			X	X	
WBGene00003155	C25D7.6.2	*mcm-3*	DNA replication licensing factor	X			X		X
WBGene00004239	Y45F10A.2.1	*puf-3*	RNA binding protein; required for cell-cycle timing, spindle positioning, pronuclei formation	X	X				X

GO, Gene Ontology; ED, embryonic development ending in birth; S, sexual reproduction; P, nucleoside, proton transport; M, meiosis; G, regulation of growth; C, cell cycle.

a Functional summaries are based on Wormbase annotations (www.wormbase.org).

### HLH-25 functions upstream of DAF-18 to regulate dauer recovery

It is not clear whether the REF-1 proteins and the HES proteins arose from the same parent gene; however, conservation within the helix-loop-helix domains and the ability to transduce Notch signals suggest that REF-1 proteins and HES proteins are members of the same functional family. Importantly, the identification of *daf-18* as a major hub in the HLH-25 transcriptional network, together with the identification of predicted HLH-25 binding sites in the *daf-18* promoter (Grove *et al.* 2009), underscores the functional homology between the HLH-25 and vertebrate HES1 protein. In the mouse thymus, HES1 acts downstream of Notch signalling to repress *Pten* activity during T-cell differentiation (Wong *et al.* 2012), and the resistance of T-cell acute lymphoblastic leukemia cells to Notch inhibition depends on functional HES1 binding sites in the *Pten* promoter (Palomero *et al.* 2007). Therefore, we further characterized the genetic interaction between the genes encoding the HES homolog HLH-25 and the PTEN homolog DAF-18.

Using RT-qPCR, we found that the levels of *daf-18* mRNA increase greater than two-fold in embryos (data not shown) and in L4 stage animals (Table 1) compared with age-matched controls. The levels of *daf-18* mRNA also increased in wild-type animals that were subjected to *hlh-25* RNA interference (Figure S5), suggesting that the increased *daf-18* activity is due to loss of HLH-25 in *hlh-25(ok1710)* animals. We failed to detect differences in *daf-18* transcriptional activity in wild-type and *hlh-25* animals expressing P*daf-18*::GFP via a previously described, integrated transgene. These animals were generated in a *daf-2* loss-of-function background, which increases the transcriptional activity of the *daf-18* promoter (Solari *et al.* 2005). It is possible that loss of *daf-2* masks the effects of HLH-25. Unfortunately, we have not yet been successful in generating stable transgenic lines expressing P*daf-18*::GFP in animals that produce functional DAF-2.

**Figure 5:**
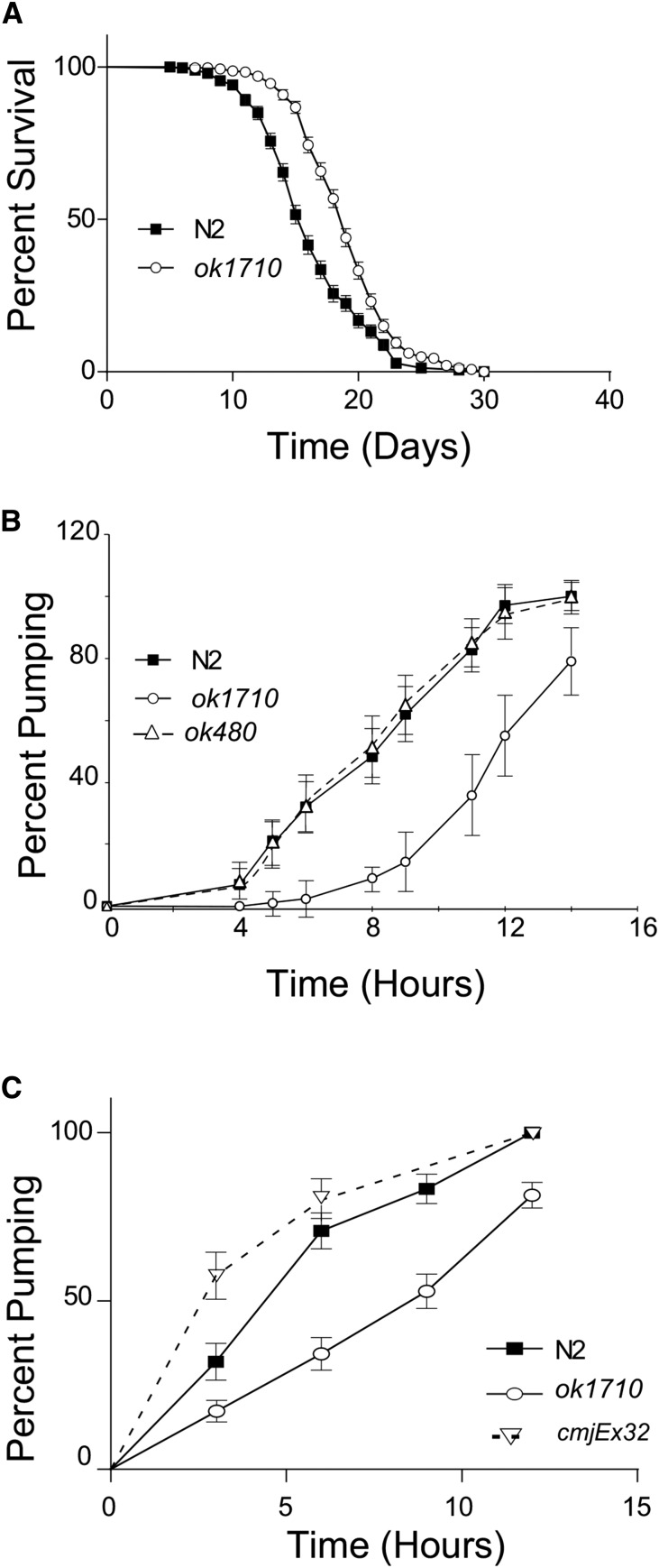
HLH-25 regulates lifespan and dauer recovery. (A) *hlh-25(ok1710)* animals (n = 282) have longer than lifespans than wild-type animals (n = 264, *P*-value < 0.0001). (B) Representative experiment showing the percentage of animals with pharyngeal pumping during dauer recovery at 20°. *P*-value < 0.0001 and *P*-value = 0.938 when *hlh-25(ok1710)* animals and *daf-18(ok480)* animals, respectively, were compared with wild-type. (C) Representative experiment showing the percentage of animals with pharyngeal pumping during dauer recovery at 20°. *P*-value < 0.0001 and *P*-value = 0.2771 when *hlh-25(ok1710)* and *hlh-25(ok1710;cmjEx32)* animals, respectively, were compared with wild-type.

In *C. elegans*, longevity and entry into dauer stage are controlled through the insulin signalling pathway, and depend on the insulin receptor DAF-2. Decreased insulin signalling, via loss-of-function mutations in *daf-2*, increases *C. elegans* lifespan and causes a dauer-constitutive phenotype. These phenotypes are suppressed in *daf-18* animals: *daf-18* animals have reduced lifespan (Solari *et al.* 2005) and animals that carry strong loss-of-function alleles are unable to undergo dauer development (Gil *et al.* 1999; Mihaylova *et al.* 1999; Rouault *et al.* 1999). The reduced lifespan of *daf-18* animals can be rescued, and slightly extended beyond the wild-type lifespan, by the overexpression of *daf-18* from the extra-chromosomal array *quIs18* (Brisbin *et al.* 2009; Solari *et al.* 2005). We reasoned that since *hlh-25(ok1710)* animals have increased *daf-18* activity, they should have an extended lifespan. As expected, the lifespan of *hlh-25(ok1710)* animals (median LS = 19 d) is significantly longer than the lifespan of wild-animals (median LS = 16 d, *P*-value <0.0001) grown at 20° (Figure 5A, Table S2). This result is comparable with the extended lifespan previously reported for *C. elegans quIs18* females (Brisbin *et al.* 2009).

Normally when wild-type, starvation-induced dauer larvae are moved to favorable growth conditions, they take approximately 60 minutes to commit to exiting the dauer stage at 25°, and they molt into the L4 stage at approximately 10 hr (Golden and Riddle 1982; Golden and Riddle 1984a,b,c). As animals begin to exit dauer stage and enter into the L4 molt, fat accumulation and pharyngeal pumping begin after approximately 60 min and 3 hr, respectively, of making the dauer recovery/exit decision (Proudfoot *et al.* 1993). The onset of vulva formation is detectable within 12 hr of the exit decision, and recovering dauers enter late L4/early adult stage within 24 hr of the decision to exit dauer stage.

Because *daf-18(ok480)* animals do not form dauers under starvation conditions, we used heat stress to induce dauer formation via an IIS-independent pathway in wild-type, *hlh-25(ok1710)*, *daf-18(ok480)*, and *quIs18* animals, and compared their dauer recovery phenotypes. We found that at least 80% of wild-type animals (n = 1199) reached early L4 stage within 12 hr after the switch to 20°. As expected, dauer recovery was similar to wild-type in *daf-18(ok480)* animals (n = 485) but was longer in *quIs18* animals (n = 955). At 12 hr, approximately 65% of the *daf-18(ok480)* animals, but only 10% of the *quIs18* animals, reached early L4. Interestingly, the dauer recovery timing of the *hlh-25(ok1710)* animals (n = 738) was very similar to the *quIs18* animals: only 21% of *hlh-25(ok1710)* animals reached L4 stage within 12 hr, and only 45% reached L4 stage within 24 hr. This delayed dauer recovery phenotype in *hlh-25(ok1710)* animals was partially rescued by transgenic expression of *hlh-25* (*cmjEx32*): 70% of transgenic animals (n = 300) reached L4 within 24 hr. For a more quantitative approach, we monitored the onset of pharyngeal pumping during dauer recovery. Dauer recovery in *daf-18(ok480)* animals was not significantly different from recovery in wild-type animals; however, it took 1.5 times longer for the onset of pharyngeal pumping to occur in 50% of *hlh-25*(*ok1710*) animals (*P*-value <0.0001) than in wild-type animals (Figure 5B). This delay was rescued by by transgenic expression of *hlh-25* (Figure 5C). These results, together with our gene expression data, suggest that HLH-25 acts upstream of *daf-18* to negatively regulate lifespan and dauer recovery.

## Discussion

This study is a functional characterization of HLH-25, a member of the *C. elegans* REF-1 family of transcriptional regulators. Previous studies have shown that the gene *hlh-25* is transcriptionally activated through Notch-signaling in cells that eventually give rise to the pharynx and nervous system, and through the Notch-independent GATA family of transcription factors in tissues that give rise to the pharynx, muscle, and somatic gonad. Here we show that expression of *hlh-25* persists throughout the *C. elegans* life cycle. Although we identified a number of HLH-25 targets that are germline expressed and whose mRNAs are stored in oocytes before fertilization, we were unable to recover transgenic lines that report germline expression of *hlh-25*. Future studies may further define the role of HLH-25 in embryogenesis, which includes determining whether the maternal contribution of HLH-25 is required for proper embryogenesis.

Our results show that the phenotypes of *hlh-25* animals include reduced brood size, unfertilized oocytes in the uterus, abnormal gonad morphology/architecture, extended lifespan, and delayed dauer recovery. In previous studies, functional annotation of candidate target genes suggested overlapping roles in cell signaling events for all of the REF-1 family proteins, and HES-like roles for HLH-25 and HLH-29 in regulating embryonic and larval development, growth, cell fate specification, and reproductive behaviors (Grove *et al.* 2009). Interestingly, this functional overlap is suggested, but not clearly evident, in the phenotypes of REF-1 family mutants that have been characterized to date. Both *ref-1* and *hlh-29* animals, for example, show varying degrees of embryonic lethality that would ultimately lead to reduced brood size (Alper and Kenyon 2001; McMiller *et al.* 2007; White *et al.* 2012); however, the reduced brood size in *hlh-25* animals in not due to lethality during embryogenesis, but to abnormalities that occur before fertilization. Likewise, mutations in *ref-1*, *hlh-29*, and *hlh-25* cause morphologic defects that affect sexual reproduction. Loss of *ref-1* affects cell fusion events during vulva morphogenesis (Alper and Kenyon 2001) and ray lineage decisions during morphogenesis of the male tail (Ross *et al.* 2005). Loss of either *hlh-29* or *hlh-25* results in unfertilized oocytes, abnormal gonad morphologies, and the exploded through vulva phenotype; however, the underlying mechanisms of these phenotypes appears to differ in *hlh-25* and *hlh-29* animals. The unfertilized oocyte phenotype in *hlh-29* animals, for example, is caused in part by altered expression of genes required for inositol 1, 4, 5-triphosphate (IP_3_) signaling, which affects the ability of oocytes and eggs to move through the spermatheca (White *et al.* 2012). On the basis of our observations of independent ovulation events, however, spermatheca function in *hlh-25* animals appears normal, and genes required for IP_3_ signaling are not affected by loss of HLH-25. These data suggest that the REF-1 family proteins do not act within a single transcriptional cascade to regulate specific events, and that each family member plays unique, stage-specific roles in *C. elegans* development.

Our gene expression microarray analysis suggests that there are six major hubs in the HLH-25 transcriptional network, including the *C. elegans* PTEN homolog, *daf-18*. We demonstrated by RT-qPCR that *daf-18* expression is normally repressed by HLH-25. This result is the first direct correlation between a HES protein target gene in mammals and a REF-1 family target gene in *C. elegans*. Hes1 directs cell division, in part, by repressing *Pten* expression in mouse (Wong *et al.* 2012), in human large cell neuroendocrine carcinoma (Nasgashio *et al.* 2011), and in regenerating Clara cells of wild-type lungs (Xing *et al.* 2012). In *C. elegans*, DAF-18 is required during embryogenesis to maintain G2 arrest in germline precursor cells (Fukuyama *et al.* 2006) via inhibition of Tor complex 1 signaling (Fukuyama *et al.* 2012). We did not examine embryonic or L1-stage phenotypes of *hlh-25* animals; however, we show that, like animals that overexpress *daf-18* (Brisbin *et al.* 2009; Liu and Chin-Sang 2015), *hlh-25* animals have an extended lifespan and take longer to exit dauer stage. Further examination of both the embryonic and postembryonic regulation of *daf-18* by HLH-25 will further our understanding of the mechanisms and biological significance of HES-dependent activation and repression of PTEN.

*C. elegans* enter the dauer stage in response to adverse growth conditions, maintain this state while conditions remain undesirable, and then exit, or recover from dauer stage when environmental conditions are more favorable (Hu 2007; Ouellet *et al.* 2008). The decision and ability to enter into dauer stage requires the integration of dietary and environmental signals through multiple molecular pathways, including insulin/insulin-like growth factor, transforming growth factor-β, and guanylyl cyclase, and the molecular details of dauer entry in response to starvation, heat stress, and overcrowding have been well established (reviewed in Fielenbach and Antebi 2008). Less is known about the molecular requirements for dauer maintenance and dauer recovery. Studies suggest that GLP-1−dependent Notch activation in chemosensory neurons is required for dauer maintenance (Ouellet *et al.* 2008) and that metabotropic acetylcholine signaling is required for the induction of an insulin-like signal during dauer recovery (Tissenbaum *et al.* 2000). Previous studies have also shown that dauer recovery requires LIN-12−dependent Notch activation, though the downstream target of this signaling event has not been identified (Ouellet *et al.* 2008). Future studies will determine whether HLH-25 acts downstream of Notch to modulate dauer recovery by repressing *daf-18* activity.

Finally, GO and protein interaction analysis of the HLH-25 network suggests that HLH-25 affects genes required for three highly connected processes: cell division, cell-cycle regulation, and reproduction, including gamete formation. The connectivity between the genes in these processes suggests that the HLH-25 regulatory network is amendable to change without the corresponding disruption in overall functionality. This feature is illustrated by the hub gene *daf-18*, and the hub genes *cgh-1*, *cyb-2.1*, *cyb-3*, *mcm-3*, and *puf-3*, which encode proteins required for early embryonic cytokinesis, meiosis II, G2/mitosis transition, licensing of DNA replication, and spindle positioning, respectively (Audhya *et al.* 2005; Boag *et al.* 2005; Hubstenberger *et al.* 2012; Kaitna *et al.* 2002; Sonneville *et al.* 2012; van der Voet *et al.* 2009). Although mutations in any of the hub genes, or in any of the nodes connected to them, result in severe phenotypes, including sterility, mutant animals are viable and have functional cell cycles. Future work will include further defining and validating the HLH-25 transcriptional network and correlating this network with the postembryonic functions of HLH-25.

Taken together, our results demonstrate that the *C. elegans* HES homolog, HLH-25, functions postembryonically to transcriptionally regulate genes required for cell division, cell-cycle regulation, and sexual reproduction, including the gene encoding the *C. elegans* Pten homolog, DAF-18.

## Supplementary Material

Supporting Information

## References

[bib1] Akazawa C. Ishibashi M. Shimizu C. Nakanishi S. Kageyama R., 1995 A mammalian helix-loop-helix factor structurally related to the product of *Drosophila* proneural gene atonal is a positive transcriptional regulator expressed in the developing nervous system. J. Biol. Chem. 270: 8730–8738. 7721778 10.1074/jbc.270.15.8730

[bib2] Alper S. Kenyon C., 2001 REF-1, a protein with two bHLH domains, alters the pattern of cell fusion in *C. elegans* by regulating Hox protein activity. Development 128: 1793–1804. 11311160 10.1242/dev.128.10.1793

[bib3] Audhya A. Hyndman F. McLeod I. X. Maddox A. S. Yates J. R. 3rd, 2005 A complex containing the Sm protein CAR-1 and the RNA helicase CGH-1 is required for embryonic cytokinesis in *Caenorhabditis elegans*. J. Cell Biol. 171: 267–279. 16247027 10.1083/jcb.200506124 PMC2171198

[bib4] Axelson H., 2004 The Notch signaling cascade in neuroblastoma: role of the basic helix-loop-helix proteins HASH-1 and HES-1. Cancer Lett. 204: 171–178. 15013216 10.1016/S0304-3835(03)00453-1

[bib5] Ben-Arie N. Hassan B. A. Bermingham N. A. Malicki D. M. Armstrong D., 2000 Functional conservation of atonal and Math1 in the CNS and PNS. Development 127: 1039–1048. 10662643 10.1242/dev.127.5.1039

[bib6] Boag P. R. Nakamura A. Blackwell T. K., 2005 A conserved RNA-protein complex component involved in physiological germline apoptosis regulation in C. elegans. Development 132: 4975–4986. 16221731 10.1242/dev.02060

[bib7] Brenner S., 1974 The genetics of *Caenorhabditis elegans*. Genetics 77: 71–94. 4366476 10.1093/genetics/77.1.71 PMC1213120

[bib8] Brisbin S. Liu J. Boudreau J. Peng J. Evangelista M., 2009 A role for C. elegans Eph RTK signaling in PTEN regulation. Dev. Cell 17: 459–469. 19853560 10.1016/j.devcel.2009.08.009

[bib9] Broitman-Maduro G. Maduro M. F. Rothman J. H., 2005 The noncanonical binding site of the MED-1 GATA factor defines differentially regulated target genes in the *C. elegans* mesendoderm. Dev. Cell 8: 427–433. 15737937 10.1016/j.devcel.2005.01.014

[bib10] Fielenbach N. Antebi A., 2008 *C. elegans* dauer formation and the molecular basis of plasticity. Genes Dev. 22: 2149–2165. 18708575 10.1101/gad.1701508 PMC2735354

[bib11] Fukuyama M. Rougvie A. E. Rothman J. H., 2006 *C. elegans* DAF-18/PTEN mediates nutrient-dependent arrest of cell cycle and growth in the germline. Curr. Biol. 16: 773–779. 16631584 10.1016/j.cub.2006.02.073

[bib12] Fukuyama M. Sakuma K. Park R. Kasuga H. Nagaya R., 2012 *C. elegans* AMPKs promote survival and arrest germline development during nutrient stress. Biol. Open 1: 929–936. 23213370 10.1242/bio.2012836 PMC3507181

[bib13] Gao F. Zhang Y. Wang S. Liu Y. Zheng L., 2014 Hes1 is involved in the self-renewal and tumourigenicity of stem-like cancer cells in colon cancer. Sci. Rep. 4: 3963. 24492635 10.1038/srep03963 PMC3912485

[bib14] Gil E. B. Malone Link E. Liu L. X. Johnson C. D. Lees J. A., 1999 Regulation of the insulin-like developmental pathway of *Caenorhabditis elegans* by a homolog of the PTEN tumor suppressor gene. Proc. Natl. Acad. Sci. USA 96: 2925–2930. 10077613 10.1073/pnas.96.6.2925 PMC15871

[bib15] Golden J. W. Riddle D. L., 1982 A pheromone influences larval development in the nematode *Caenorhabditis elegans*. Science 218: 578–580. 6896933 10.1126/science.6896933

[bib16] Golden J. W. Riddle D. L., 1984 a A *Caenorhabditis elegans* dauer-inducing pheromone and an antagonistic component of the food supply. J. Chem. Ecol. 10: 1265–1280. 24318910 10.1007/BF00988553

[bib17] Golden J. W. Riddle D. L., 1984 b The *Caenorhabditis elegans* dauer larva: developmental effects of pheromone, food, and temperature. Dev. Biol. 102: 368–378. 6706004 10.1016/0012-1606(84)90201-x

[bib18] Golden J. W. Riddle D. L., 1984 c A pheromone-induced developmental switch in *Caenorhabditis elegans*: Temperature-sensitive mutants reveal a wild-type temperature-dependent process. Proc. Natl. Acad. Sci. USA 81: 819–823. 6583682 10.1073/pnas.81.3.819 PMC344929

[bib19] Govindan J. A. Nadarajan S. Kim S. Starich T. A. Greenstein D., 2009 Somatic cAMP signaling regulates MSP-dependent oocyte growth and meiotic maturation in *C. elegans*. Development 136: 2211–2221. 19502483 10.1242/dev.034595 PMC2729340

[bib20] Green R. A. Kao H. L. Audhya A. Arur S. Mayers J. R., 2011 A high-resolution *C. elegans* essential gene network based on phenotypic profiling of a complex tissue. Cell 145: 470–482. 21529718 10.1016/j.cell.2011.03.037 PMC3086541

[bib21] Grove C. A. De Masi F. Barrasa M. I. Newburger D. E. Alkema M. J., 2009 A multiparameter network reveals extensive divergence between *C. elegans* bHLH transcription factors. Cell 138: 314–327. 19632181 10.1016/j.cell.2009.04.058 PMC2774807

[bib22] Harris T. W. Antoshechkin I. Bieri T. Blasiar D. Chan J., 2010 WormBase: a comprehensive resource for nematode research. Nucleic Acids Res. 38: D463–D467. 19910365 10.1093/nar/gkp952 PMC2808986

[bib23] Hermann G. J. Leung B. Priess J. R., 2000 Left-right asymmetry in *C. elegans* intestine organogenesis involves a LIN-12/Notch signaling pathway. Development 127: 3429–3440. 10903169 10.1242/dev.127.16.3429

[bib24] Hoogewijs D. Houthoofd K. Matthijssens F. Vandesompele J. Vanfleteren J. R., 2008 Selection and validation of a set of reliable reference genes for quantitative sod gene expression analysis in *C. elegans*. BMC Mol. Biol. 9: 9. 18211699 10.1186/1471-2199-9-9 PMC2254638

[bib25] Hu, P. J., 2007 Dauer (August 8, 2007), WormBook, ed. The C. elegans Research Community WormBook, /10.1895/wormbook.1.144.1, http//www.wormbook.org.

[bib26] Huang da W. Sherman B. T. Lempicki R. A, 2009 a Bioinformatics enrichment tools: paths toward the comprehensive functional analysis of large gene lists. Nucleic Acids Res. 37: 1-13. 19033363 10.1093/nar/gkn923 PMC2615629

[bib27] Huang da W. Sherman B. T. Lempicki R. A., 2009 b Systematic and integrative analysis of large gene lists using DAVID bioinformatics resources. Nat. Protoc. 4: 44–57. 19131956 10.1038/nprot.2008.211

[bib28] Hubbard E. J. Greenstein D., 2000 The *Caenorhabditis elegans* gonad: a test tube for cell and developmental biology. Dev. Dyn. 218: 2–22. 10822256 10.1002/(SICI)1097-0177(200005)218:1<2::AID-DVDY2>3.0.CO;2-W

[bib29] Hubstenberger A. Cameron C. Shtofman R. Gutman S. Evans T. C., 2012 A network of PUF proteins and Ras signaling promote mRNA repression and oogenesis in *C. elegans*. Dev. Biol. 366: 218–231. 22542599 10.1016/j.ydbio.2012.03.019 PMC3361503

[bib30] Kaitna S. Schnabel H. Schnabel R. Hyman A. A. Glotzer M., 2002 A ubiquitin C-terminal hydrolase is required to maintain osmotic balance and execute actin-dependent processes in the early *C. elegans* embryo. J. Cell Sci. 115: 2293–2302. 12006614 10.1242/jcs.115.11.2293

[bib31] Kamath R. S. Fraser A. G. Dong Y. Poulin G. Durbin R., 2003 Systematic functional analysis of the *Caenorhabditis elegans* genome using RNAi. Nature 421: 231–237. 12529635 10.1038/nature01278

[bib32] Lanjuin A. Claggett J. Shibuya M. Hunter C. P. Sengupta P., 2006 Regulation of neuronal lineage decisions by the HES-related bHLH protein REF-1. Dev. Biol. 290: 139–151. 16376329 10.1016/j.ydbio.2005.11.018

[bib33] Lewis J. A. Fleming J. T., 1995 Basic culture methods. Methods Cell Biol. 48: 3–29. 8531730

[bib34] Li P. Collins K. M. Koelle M. R. Shen K., 2013 LIN-12/Notch signaling instructs postsynaptic muscle arm development by regulating UNC-40/DCC and MADD-2 in *Caenorhabditis elegans*. eLife 2: e00378. 23539368 10.7554/eLife.00378 PMC3601818

[bib35] Liu J. Chin-Sang I. D., 2015 *C. elegans* as a model to study PTEN’s regulation and function. Methods 77-78: 180–190. 25514044 10.1016/j.ymeth.2014.12.009

[bib36] Masse I. Molin L. Billaud M. Solari F., 2005 Lifespan and dauer regulation by tissue-specific activities of *Caenorhabditis elegans* DAF-18. Dev. Biol. 286: 91–101. 16153634 10.1016/j.ydbio.2005.07.010

[bib37] McMiller T. L. Sims D. Lee T. Williams T. Johnson C. M., 2007 Molecular characterization of the *Caenorhabditis elegans* REF-1 family member, hlh-29/hlh-28. Biochim. Biophys. Acta 1769: 5–19. 17258327 10.1016/j.bbaexp.2006.12.001

[bib38] Mihaylova V. T. Borland C. Z. Manjarrez L. Stern M. J. Sun H., 1999 The PTEN tumor suppressor homolog in *Caenorhabditis elegans* regulates longevity and dauer formation in an insulin receptor-like signaling pathway. Proc. Natl. Acad. Sci. USA 96: 7427–7432. 10377431 10.1073/pnas.96.13.7427 PMC22102

[bib39] Miller R. M. Portman D. S., 2011 The Wnt/beta-catenin asymmetry pathway patterns the atonal ortholog lin-32 to diversify cell fate in a *Caenorhabditis elegans* sensory lineage. J. Neurosci. 31: 13281–13291. 21917811 10.1523/JNEUROSCI.6504-10.2011 PMC3183998

[bib40] Nasgashio R. Sato Y. Matsumoto T. Kageyama T. Hattori M., 2011 The balance between the expressions of hASH1 and HES1 differs between large cell neuroendocrine carcinoma and small cell carcinoma of the lung. Lung Cancer 74: 405–410. 21601304 10.1016/j.lungcan.2011.04.012

[bib41] Neves A. Priess J. R., 2005 The REF-1 family of bHLH transcription factors pattern *C. elegans* embryos through Notch-dependent and Notch-independent pathways. Dev. Cell 8: 867–879. 15935776 10.1016/j.devcel.2005.03.012

[bib42] Neves A. English K. Priess J. R., 2007 Notch-GATA synergy promotes endoderm-specific expression of ref-1 in *C. elegans*. Development 134: 4459–4468. 18003741 10.1242/dev.008680

[bib43] Ouellet J. Li S. Roy R., 2008 Notch signalling is required for both dauer maintenance and recovery in *C. elegans*. Development 135: 2583–2592. 18599512 10.1242/dev.012435

[bib44] Ouji Y. Ishizaka S. Nakamura-Uchiyama F. Wanaka A. Yoshikawa M., 2013 Induction of inner ear hair cell-like cells from Math1-transfected mouse ES cells. Cell Death Dis. 4: e700. 23828563 10.1038/cddis.2013.230 PMC3730404

[bib45] Palomero T. Sulis M. L. Cortina M. Real P. J. Barnes K., 2007 Mutational loss of PTEN induces resistance to NOTCH1 inhibition in T-cell leukemia. Nat. Med. 13: 1203–1210. 17873882 10.1038/nm1636 PMC2600418

[bib46] Powell L. M. Zur Lage P. I. Prentice D. R. Senthinathan B. Jarman A. P., 2004 The proneural proteins Atonal and Scute regulate neural target genes through different E-box binding sites. Mol. Cell. Biol. 24: 9517–9526. 15485919 10.1128/MCB.24.21.9517-9526.2004 PMC522279

[bib47] Proudfoot L. Kusel J. R. Smith H. V. Harnett W. Worms M. J., 1993 Rapid changes in the surface of parasitic nematodes during transition from pre- to post-parasitic forms. Parasitology 107: 107–117. 8355993 10.1017/s0031182000079464

[bib48] Quach T. K. Chou H. T. Wang K. Milledge G. Z. Johnson C. M., 2013 Genome-wide microarrray analysis reveals roles for the REF-1 family member HLH-29 in ferritin synthesis and peroxide stress response. PLoS One 8: e59719. 23533643 10.1371/journal.pone.0059719 PMC3606163

[bib49] Riesen M. Feyst I. Rattanavirotkul N. Ezcurra M. Tullet J. M., 2014 MDL-1, a growth- and tumor-suppressor, slows aging and prevents germline hyperplasia and hypertrophy in *C. elegans*. Aging (Albany) 6: 98−117. 10.18632/aging.100638 PMC3969279 24531613

[bib50] Ross J. M. Kalis A. K. Murphy M. W. Zarkower D., 2005 The DM domain protein MAB-3 promotes sex-specific neurogenesis in *C. elegans* by regulating bHLH proteins. Dev. Cell 8: 881–892. 15935777 10.1016/j.devcel.2005.03.017

[bib51] Rouault J. P. Kuwabara P. E. Sinilnikova O. M. Duret L. Thierry-Mieg D., 1999 Regulation of dauer larva development in *Caenorhabditis elegans* by daf-18, a homologue of the tumour suppressor PTEN. Curr. Biol. 9: 329–332. 10209098 10.1016/s0960-9822(99)80143-2

[bib52] Sang L. Roberts J. M. Coller H. A., 2010 Hijacking HES1: how tumors co-opt the anti-differentiation strategies of quiescent cells. Trends Mol. Med. 16: 17–26. 20022559 10.1016/j.molmed.2009.11.001 PMC2864914

[bib53] Schnabel, R., and J. R. Priess, 1997 *Specification of Cell Fates in the Early Embryo*, pp. 361–382 in *C. elegans II*, Ed. 2, edited by D. L. Riddle, T. Blumenthal, B. J. Meyer, and J. R. Priess. Cold Spring Harbor Laboratory Press, Cold Spring Harbor, New York. 21413232

[bib54] Solari F. Bourbon-Piffaut A. Masse I. Payrastre B. Chan A. M., 2005 The human tumour suppressor PTEN regulates longevity and dauer formation in *Caenorhabditis elegans*. Oncogene 24: 20–27. 15637588 10.1038/sj.onc.1207978

[bib55] Sonneville R. Querenet M. Craig A. Gartner A. Blow J. J., 2012 The dynamics of replication licensing in live *Caenorhabditis elegans* embryos. J. Cell Biol. 196: 233–246. 22249291 10.1083/jcb.201110080 PMC3265957

[bib56] Szklarczyk D. Franceschini A. Kuhn M. Simonovic M. Roth A., 2011 The STRING database in 2011: functional interaction networks of proteins, globally integrated and scored. Nucleic Acids Res. 39: D561–D568. 21045058 10.1093/nar/gkq973 PMC3013807

[bib57] Tissenbaum H. A. Hawdon J. Perregaux M. Hotez P. Guarente L., 2000 A common muscarinic pathway for diapause recovery in the distantly related nematode species *Caenorhabditis elegans* and *Ancylostoma caninum*. Proc. Natl. Acad. Sci. USA 97: 460–465. 10618440 10.1073/pnas.97.1.460 PMC26685

[bib58] Tomita K. Moriyoshi K. Nakanishi S. Guillemot F. Kageyama R., 2000 Mammalian achaete-scute and atonal homologs regulate neuronal versus glial fate determination in the central nervous system. EMBO J. 19: 5460–5472. 11032813 10.1093/emboj/19.20.5460 PMC314003

[bib59] van der Voet M. Lorson M. A. Srinivasan D. G. Bennett K. L. van den Heuvel S., 2009 *C. elegans* mitotic cyclins have distinct as well as overlapping functions in chromosome segregation. Cell Cycle 8: 4091–4102. 19829076 10.4161/cc.8.24.10171 PMC3614003

[bib60] Wang S. W. Kim B. S. Ding K. Wang H. Sun D., 2001 Requirement for math5 in the development of retinal ganglion cells. Genes Dev. 15: 24–29. 11156601 10.1101/gad.855301 PMC312600

[bib61] Ward S. Carrel J. S., 1979 Fertilization and sperm competition in the nematode *Caenorhabditis elegans*. Dev. Biol. 73: 304–321. 499670 10.1016/0012-1606(79)90069-1

[bib62] White A. Fearon A. Johnson C. M., 2012 HLH-29 regulates ovulation in *C. elegans* by targeting genes in the inositol triphosphate signaling pathway. Biol. Open 1: 261–268. 23213416 10.1242/bio.2012046 PMC3507288

[bib63] Wong G. W. Knowles G. C. Mak T. W. Ferrando A. A. Zuniga-Pflucker J. C., 2012 HES1 opposes a PTEN-dependent check on survival, differentiation, and proliferation of TCRbeta-selected mouse thymocytes. Blood 120: 1439–1448. 22649105 10.1182/blood-2011-12-395319 PMC3423782

[bib64] Wrischnik L. A. Kenyon C. J., 1997 The role of lin-22, a hairy/enhancer of split homolog, in patterning the peripheral nervous system of *C. elegans*. Development 124: 2875–2888. 9247331 10.1242/dev.124.15.2875

[bib65] Xing Y. Li A. Borok Z. Li C. Minoo P., 2012 NOTCH1 is required for regeneration of Clara cells during repair of airway injury. Stem Cells 30: 946–955. 22331706 10.1002/stem.1059 PMC4005608

