# Supplementary material for: HES-Mediated Repression of Pten in Caenorhabditis elegans
Source: G3 (Bethesda). 2015 Oct 4;5(12):2619–28. doi: 10.1534/g3.115.019463 (PMC4683635; doi:10.1534/g3.115.019463)
Supplement: Supporting Information [file supp_5_12_2619__index.html]

HES-Mediated Repression of Pten in Caenorhabditis elegans — Supporting Information 

# HES-Mediated Repression of Pten in *Caenorhabditis elegans*

## Supporting Information for Chou et al., 2015

**Files in this Data Supplement:**

- Supporting Information - Figures S1-S5 and Tables S1-S3 (PDF, 1.4 MB)
- Figure S1 - Mutant allele and extrachromosomal arrays for *hlh-25* (PDF, 477 KB)
- Figure S2 - *hlh-25(ok1710)* gonad architecture and oocyte morphology phenotypes (PDF, 581 KB)
- Figure S3 - *hlh-25(ok1710)* gonad architecture and oocyte morphology phenotypes (PDF, 520 KB)
- Figure S4 - *hlh-25(ok1710)* animals move slower than wild-type (PDF, 541 KB)
- Figure S5 - *daf-18* mRNA Levels in Adult Stage Animals (PDF, 512 KB)
- Table S1 - Average numbers of live progeny and unfertilized oocytes per day of egg laying per animal (PDF, 360 KB)
- Table S2 - Lifespan Measurements (PDF, 360 KB)
- Table S3 - Genes changed by 2.0 fold or greater in *hlh-25(ok1710)* animals (PDF, 729 KB)
