# Supplementary material for: HES-Mediated Repression of Pten in Caenorhabditis elegans
Source: G3 (Bethesda). 2015 Oct 4;5(12):2619–28. doi: 10.1534/g3.115.019463 (PMC4683635; doi:10.1534/g3.115.019463)
Supplement: Supporting Information [file supp_g3.115.019463_019463SI.pdf]

HES-mediated Repression of PTEN in *C. elegans*

Han Ting Chou[*§], Raymarie Gomez Vazquez[*], Kun Wang[#], Richard Campbell[*], Walter W. Walthall[*], Casonya M. Johnson[*§1]

[*]Department of Biology and [§]Center for Obesity Reversal, Georgia State University, Atlanta, GA 30303, [#]Department of Environmental Health Science, Division of Biostatistics, New York University School of Medicine, New York, NY 10016

[1]Corresponding author: Georgia State University, Dept. of Biology, 161 Jesse Hill Jr. Dr., SE., Atlanta, GA 30303. E-mail: cjohnson113@gsu.edu

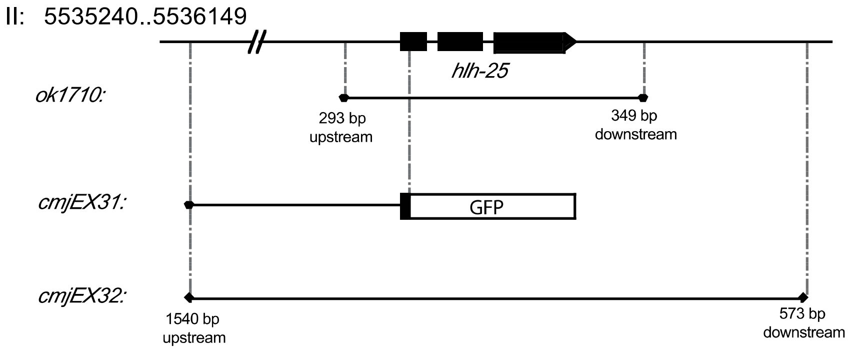

Figure S1. Mutant allele and extrachromosomal arrays for *hlh-25.* The *hlh-25(ok1710)* allele is a 1550 bp deletion of chromosome II that removes the entire coding region for *hlh-25* and additional flanking sequences. The extrachromosomal array *cmjEx31* (II:5533700..5535270::GFP)is a transcriptional fusion that contains 1540 base pairs upstream of the *hlh-25* initiator codon and the first 30 nucleotides of the *hlh-25* coding region. These sequences are fused in-frame to the GFP coding sequences. The extrachromosomal array cmjEx32 (II:5533700..5536722) contains genomic DNA sequences ranging from 1540 bp upstream of the *hlh-25* initiator codon to 573 bp downstream of the *hlh-25* terminator codon.

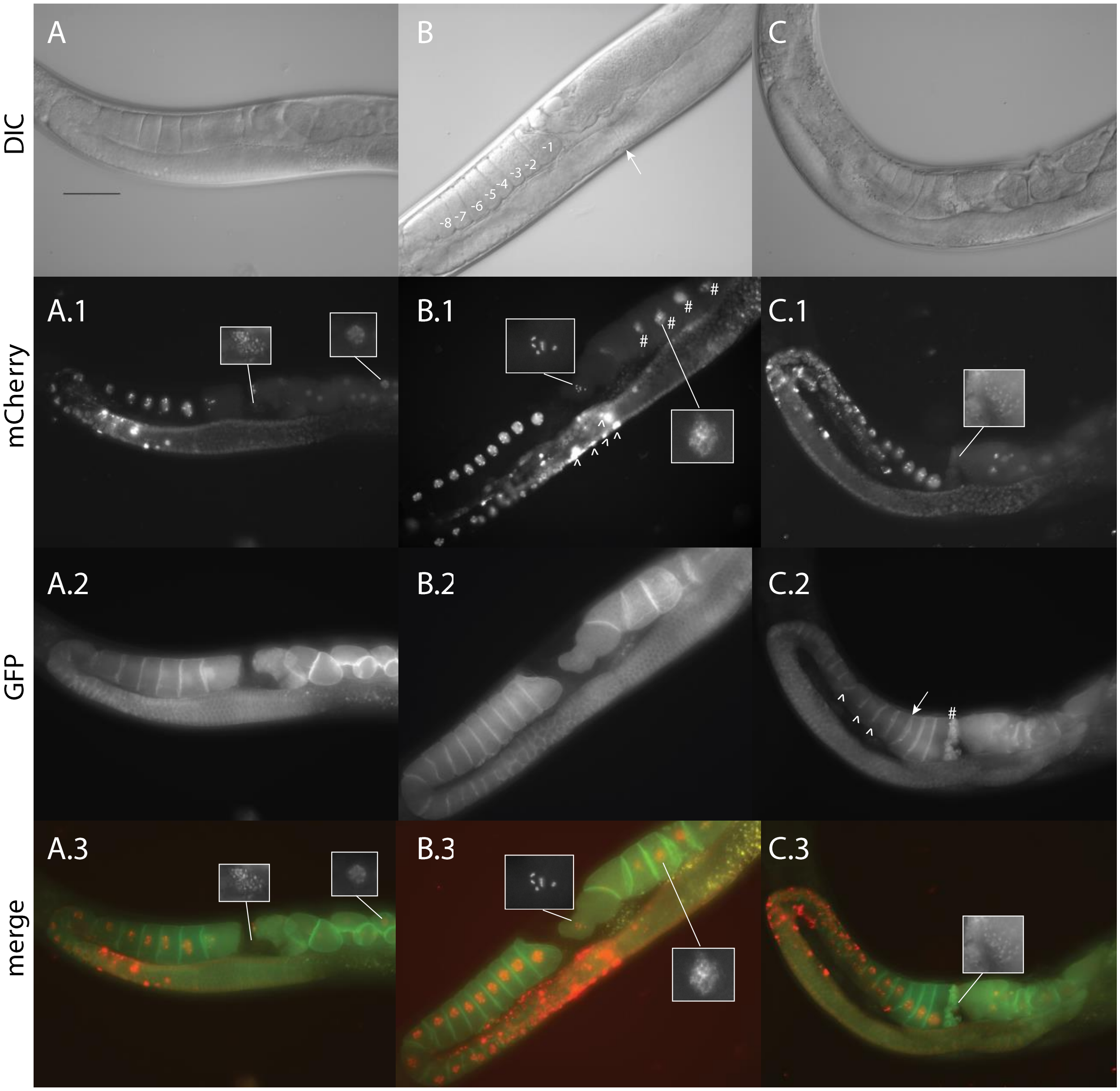

Figure S2. ***hlh-25(ok1710)* gonad architecture and oocyte morphology phenotypes.** Representative 1) Normarski, 2) mCherry, 3) GFP, and 4) merged mCherry/GFP images of A) wild-type gonad arm seen in Figure 3 and B-C) *hlh-25(ok1710)* gonad arms showing B) increased oocyte number, distal gonad arm constriction (arrow), increased apoptosis (^), abnormal embryonic chromatin (insert, right) and arrested embryos in uterus (#); C) P14,5P_2 debris (#), irregular cellular junctions (arrow), incomplete oocyte compartmentalization (^). Scale bar is 50 µM. In all images ventral is up.

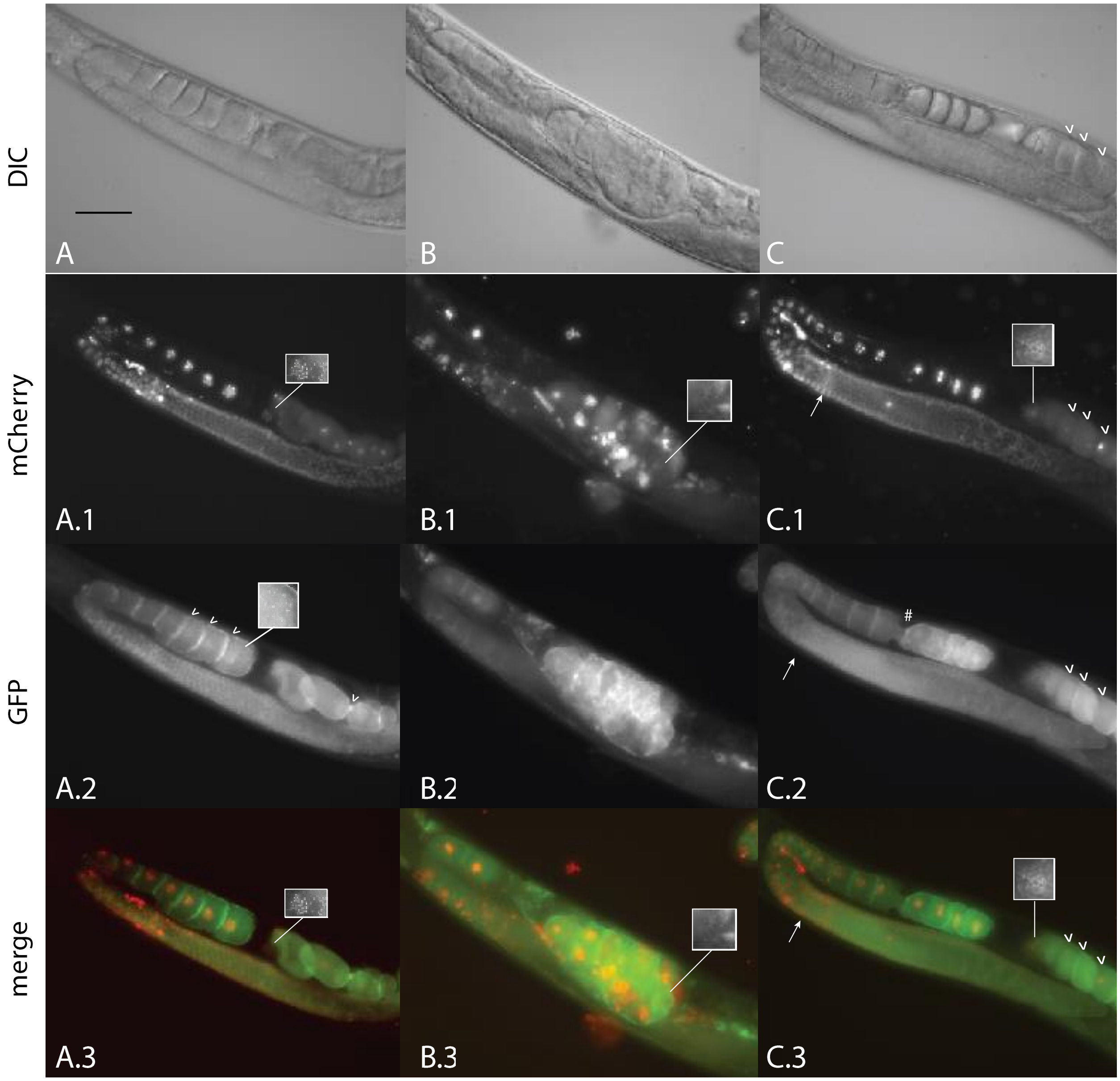

Figure S3. ***hlh-25(ok1710)*** **gonad architecture and oocyte morphology phenotypes.** Representative 1) Normarski, 2) mCherry, 3) GFP, and 4) merged mCherry/GFP images of *hlh-25(ok1710)* gonad arms showing A) irregular cellular junctions (^) and cytoplasmic debris in oocytes (insert), B) gonad arm tumor with oocytes containing decondensed chromatin and irregular nuclei. Note position of sperm (inserts) and presumed spermatheca, relative to tumor. Morphology of the distal arm was difficult to capture in image, but showed mild pathfinding defects; C) proximal (white arrow) and distal (#) constrictions of the gonad arm, and abnormal embryos in spermatheca/uterus (^). The proximal constriction is similar to the one resulting in a gonad arm tumor in B. Scale bar is 50 µM. In all images, ventral is up.

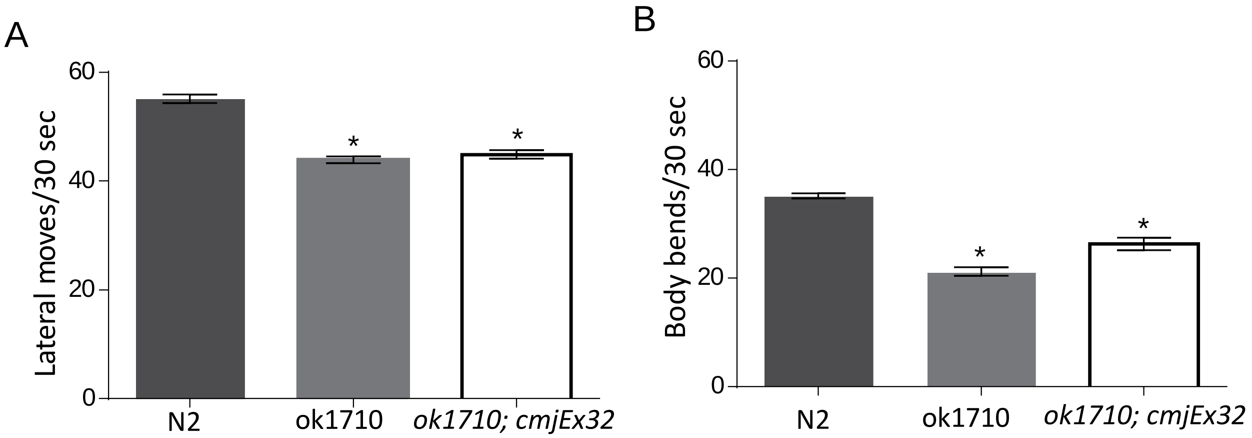

Figure S4. *hlh-25(ok1710)* animals move slower than wild-type. A) Thrashing and B) Locomotion assays comparing *hlh-25(ok1710)* animals (grey bars) to wild-type (black bars) animals. Transgenic expression of cmjEx32 did not rescue the locomotion defects in transgenic *hlh-25(ok1710)* animals (white bars) transgene did not rescue the locomotion defects in null animals. Significance was determined using the student's T test. *,P-value <0.0001.

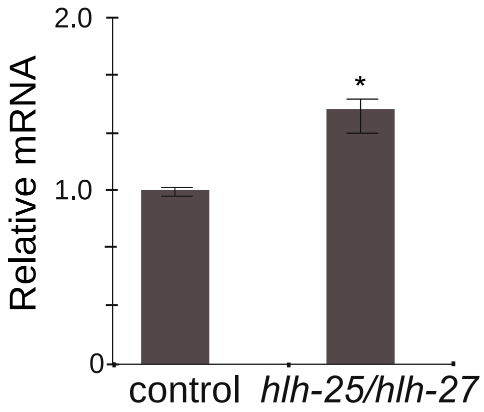

Figure S5. *daf-18* mRNA Levels in Adult Stage Animals. RT-qPCR measurements of *daf-18* mRNA in animals subjected *hlh-25/hlh-27* RNAi relative to animals subjected to control (*unc-55*) RNAi. Error bars represent stand error of the mean (SEM) *P-value <0.05.

Table S1. Average numbers of live progeny and unfertilized oocytes per day of egg laying per animal.

	Days of Egg-Laying										
	**1**	**2**	**3**	**4**	**5**	**6**	**7**	**8**	**9**	**10**	**TOTAL**
WT brood size	0.0	114.6	132.1	20.9	0.1	0.0	0.0	0.0	0.0	0.0	268
ok1710 brood size	0.9	65.1	93.5	18.6	0.9	0.3	0.1	0.0	0.0	0.0	179
WT oocytes laid	0.0	0.0	8.4	33.1	6.9	0.9	0.0	0.0	0.0	0.0	49
ok1710 oocytes laid	0.0	1.9	11.3	90.9	66.1	37.3	15.3	6.6	2.2	0.0	232

Table S2. Lifespan Measurements

Strain	Biological Replicate	Total # of Animals to Die/or to be Censored	Median Life Span of All Animals (days)	P-value Log-Rank Test compared to N2 animals
N2	1	88/13	15	
	2	90/10	17	
	3	86/12	15	
	**all**	**264/35**	**16**	
hlh-25	1	93/7	20	< 0.0001
	2	93/7	19	0.0046
	3	96/4	19	< 0.0001
	**all**	**282/18**	**19**	**< 0.0001**

Table S3 Genes changed by 2.0 fold or greater in *hlh-25(ok1710)* animals.

Genes Downregulated in *hlh-25* Mutants						
Probe Set ID	FCAbsolute	regulation	log2 FC	Gene Symbol	WormBase Gene ID	Entrez Gene
192195_at	3.28185	down	-1.71451	acs-2	WBGene00009221	3565680
174675_at	9.91220	down	-3.30920	acs-2	WBGene00009221	3565680
**175809_at**	**2.54856**	**down**	**-1.34968**	**akt-2***	**WBGene00000103**	**181524**
174489_at	3.19418	down	-1.67544	anc-1	WBGene00000140	172034
179272_at	3.27792	down	-1.71278	C06B3.6	WBGene00007365	182293
186281_s_at	2.94725	down	-1.55937	C08F11.12	WBGene00007459	178333
180306_at	2.03640	down	-1.02602	C10G8.4	WBGene00015683	182503
187800_at	2.35348	down	-1.23479	C16H3.3	WBGene00015868	181787
178441_at	3.74486	down	-1.90491	C34C6.7	WBGene00007919	174393
175482_at	2.40693	down	-1.26719	C34E10.10	WBGene00016411	175715
178672_s_at	2.02000	down	-1.01435	C45E1.4	WBGene00016663	171885
188382_s_at	2.02560	down	-1.01835	C52B9.8*	WBGene00016868	180705
173429_at	2.26820	down	-1.18155	cdk-4	WBGene00000406	181472
175601_at	2.07108	down	-1.05038	ceh-20	WBGene00000443	3565545
173465_at	2.41219	down	-1.27034	chk-1	WBGene00000498	3565921
175786_at	2.34089	down	-1.22706	cki-1	WBGene00000516	174260
190344_at	2.20686	down	-1.14199	clec-196	WBGene00009156	178506
175764_s_at	2.57972	down	-1.36722	clh-3	WBGene00000530	174187
173398_s_at	2.55895	down	-1.35555	cnc-7*	WBGene00010005	180337
172240_x_at	3.20165	down	-1.67882	cnc-7 *	WBGene00010005	180337
175247_s_at	2.17671	down	-1.12215	cTel55X.1	WBGene00007068	181791
188900_at	2.57840	down	-1.36648	ctr-9*	WBGene00007184	176335
175602_at	2.01796	down	-1.01289	cul-1	WBGene00000836	176466
**173185_s_at**	**2.15732**	**down**	**-1.10924**	**D1043.1**	**WBGene00008361**	**174777**
190646_s_at	2.01751	down	-1.01257	ddo-2	WBGene00017565	179130
193932_s_at	2.00236	down	-1.00170	dnj-17	WBGene00001035	176761
182470_at	5.60096	down	-2.48568	F09F7.6	WBGene00017303	184260
174649_s_at	2.01300	down	-1.00935	F13H8.2	WBGene00017435	174038
180425_s_at	3.02041	down	-1.59474	F15E6.3	WBGene00017484	177195
186521_at	2.01465	down	-1.01053	F21C10.10*	WBGene00017659	179290
187613_at	2.11831	down	-1.08292	F22D6.8*	WBGene00043279	4363015
174514_at	2.12010	down	-1.08413	F26F12.3	WBGene00017834	178972
173086_s_at	2.93479	down	-1.55326	F26F12.3	WBGene00017834	178972
174420_at	2.25216	down	-1.17131	F29C6.1	WBGene00009243	172947

174067_at	2.02129	down	-1.01528	F33H1.4	WBGene00009365	174580
173629_at	2.28682	down	-1.19334	F36A2.13	WBGene00009460	172697
187019_at	2.08845	down	-1.06243	F37C12.10	WBGene00018153	185422
187391_at	2.00271	down	-1.00195	F40B5.1	WBGene00018225	185512
173550_at	2.72834	down	-1.44802	F45D3.4*	WBGene00009724	179718
175278_at	2.86457	down	-1.51832	F46A8.7*	WBGene00009750	185823
180098_s_at	2.48319	down	-1.31220	F49E2.5	WBGene00009888	181175
174012_s_at	2.22870	down	-1.15620	F53B2.8	WBGene00009957	178203
174415_at	2.14591	down	-1.10159	F53B3.5	WBGene00018743	180578
182757_at	2.22093	down	-1.15116	F53F4.14*	WBGene00009996	179852
179078_s_at	2.03969	down	-1.02835	F54C9.3	WBGene00010041	174369
178192_at	2.12972	down	-1.09066	F54C9.9	WBGene00010044	174374
182088_s_at	38.09506	down	-5.25153	F57F4.4	WBGene00019017	3564957
179523_at	2.26317	down	-1.17835	F59A2.5*	WBGene00010305	175444
173696_at	2.14082	down	-1.09816	fbf-2	WBGene00001402	174017
172220_x_at	2.10182	down	-1.07164	fipr-23	WBGene00007989	173283
191759_at	3.02902	down	-1.59885	fmo-2*	WBGene00001477	177958
174574_at	2.19332	down	-1.13312	gmps-1	WBGene00010912	174672
173341_at	5.02048	down	-2.32783	haf-6	WBGene00001816	171694
**194214_x_at**	**2.04421**	**down**	**-1.03155**	**hcp-2**	**WBGene00001830**	**179353**
191712_s_at	2.26523	down	-1.17965	icl-1	WBGene00001564	178583
173301_s_at	4.87536	down	-2.28551	icl-1	WBGene00001564	178583
194142_x_at	3.12544	down	-1.64406	icl-1	WBGene00001564	178583
180721_at	5.41142	down	-2.43601	ilys-2	WBGene00016669	183475
180946_at	6.65323	down	-2.73405	ilys-3	WBGene00016670	177033
180815_at	2.02369	down	-1.01698	K01H12.1	WBGene00010484	186854
172998_s_at	2.04023	down	-1.02873	K07A1.6	WBGene00010613	172797
173190_s_at	2.11630	down	-1.08155	K08A2.4*	WBGene00019513	173643
191313_at	2.02656	down	-1.01903	K10C3.5	WBGene00010732	172838
173634_at	2.03807	down	-1.02720	lact-3*	WBGene00010897	174644
187999_at	2.12534	down	-1.08769	lea-1	WBGene00002263	3564838
174552_s_at	3.26460	down	-1.70690	lea-1	WBGene00002263	3564838
173232_s_at	2.25943	down	-1.17596	lmp-2*	WBGene00015471	180467
184694_at	7.69971	down	-2.94480	lys-10	WBGene00003099	184622
**173362_s_at**	**2.44661**	**down**	**-1.29079**	**M03C11.2***	**WBGene00010839**	**176457**
181968_s_at	2.19844	down	-1.13648	mac-1	WBGene00003119	174974
194188_x_at	2.35817	down	-1.23767	mps-2	WBGene00019282	173419
**193857_s_at**	**3.26952**	**down**	**-1.70908**	**msh-5**	**WBGene00003421**	**178268**
172744_at	17.33867	down	-4.11592	mtl-1*	WBGene00003473	179060

178512_at	2.88412	down	-1.52813	nlp-39	WBGene00008295	183774
172268_x_at	2.14778	down	-1.10284	nspc-20*	WBGene00008356	181549
174923_at	2.08022	down	-1.05673	ntl-4*	WBGene00003827	177580
173471_at	2.14734	down	-1.10255	plk-2*	WBGene00004043	171838
188279_at	2.44021	down	-1.28701	polk-1	WBGene00017696	176209
173487_at	2.28395	down	-1.19153	R04D3.2	WBGene00011010	181451
191364_at	2.36960	down	-1.24465	R05H10.5	WBGene00011045	187630
179476_at	2.21504	down	-1.14733	R07E5.1	WBGene00011109	175574
190514_at	6.27039	down	-2.64856	R10E4.11	WBGene00011206	175554
180522_at	2.22923	down	-1.15654	R11G1.2	WBGene00020011	187816
**189050_at**	**2.21158**	**down**	**-1.14508**	**R74.6**	**WBGene00011280**	**187888**
185637_at	2.01391	down	-1.01000	roik-1	WBGene00019698	171883
**190859_s_at**	**4.16545**	**down**	**-2.05847**	**sel-5**	**WBGene00004762**	**175599**
173195_s_at	2.41617	down	-1.27272	smu-2	WBGene00004896	173693
188388_at	2.63219	down	-1.39626	spg-7	WBGene00004978	171915
178149_s_at	2.11180	down	-1.07847	spp-1	WBGene00004986	176452
174411_s_at	2.04177	down	-1.02982	T01H3.2	WBGene00011348	174271
174180_at	2.14385	down	-1.10020	T02E9.5	WBGene00011383	179568
177899_at	3.30412	down	-1.72426	T03D8.7	WBGene00011394	180363
179805_s_at	2.02226	down	-1.01597	T04G9.7	WBGene00020218	180424
186427_at	2.13525	down	-1.09441	T05H4.11*	WBGene00020274	179024
174789_at	2.04914	down	-1.03502	T09B4.5	WBGene00020379	172300
172193_at	9.47063	down	-3.24346	T12D8.5	WBGene00011733	176797
179094_s_at	2.10954	down	-1.07693	T16G1.4*	WBGene00011798	179776
173020_s_at	3.44097	down	-1.78281	T19A5.1 *	WBGene00020553	179275
173496_s_at	3.60722	down	-1.85089	T23G7.3	WBGene00011966	174456
193610_s_at	3.24662	down	-1.69894	taf-1	WBGene00006382	173257
171855_x_at	2.30497	down	-1.20475	tcc-1	WBGene00013340	180167
181127_s_at	2.86219	down	-1.51712	thoc-2	WBGene00015813	175900
193643_s_at	2.49799	down	-1.32077	trxr-1*	WBGene00015553	177466
171783_x_at	2.56619	down	-1.35963	ubc-1	WBGene00006701	177170
175566_s_at	2.27868	down	-1.18820	ugt-50	WBGene00011564	181413
174606_at	2.12963	down	-1.09060	utx-1	WBGene00017046	181110
189587_s_at	2.72560	down	-1.44657	W03F9.4	WBGene00021002	178543
174109_at	6.97238	down	-2.80165	Y102A5C.6	WBGene00014955	
187234_s_at	4.36119	down	-2.12472	Y110A2AL.9	WBGene00022445	173632
171895_s_at	2.24218	down	-1.16490	Y17G7B.17	WBGene00012468	174849
174752_at	2.25226	down	-1.17137	Y39A3CL.1	WBGene00021440	175329
172668_x_at	2.30313	down	-1.20360	Y39A3CL.3*	WBGene00021441	189725

173609_at	2.94121	down	-1.55641	Y39B6A.1	WBGene00012664	180264
176082_s_at	2.16887	down	-1.11694	Y41D4B.6	WBGene00021513	176974
181789_at	2.35508	down	-1.23578	Y47H9C.8	WBGene00012951	173068
173297_at	2.49411	down	-1.31852	Y48G8AL.13*	WBGene00021691	171699
173257_s_at	2.64024	down	-1.40067	Y53F4B.45	WBGene00044728	
176209_at	2.02557	down	-1.01833	Y58A7A.5*	WBGene00021979	190383
176183_at	4.64676	down	-2.21623	Y69A2AR.25	WBGene00022096	190546
185811_at	2.05850	down	-1.04159	Y71H2B.4	WBGene00022194	175381
173410_s_at	2.00814	down	-1.00586	Y87G2A.1	WBGene00013593	173221
180427_at	3.00260	down	-1.58621	ZC395.5*	WBGene00022597	191152
173558_at	2.81234	down	-1.49177	ZC443.3	WBGene00013898	179758
174073_at	4.64250	down	-2.21490			

Genes Upregulated in *hlh-25* Mutants

Probe Set ID	FCAbsolute	regulation	log2 FC	Gene Symbol	WormBase Gene ID	Entrez Gene
177193_s_at	4.99339	up	2.32002	abu-1*	WBGene00000024	181800
179983_at	4.79301	up	2.26093	abu-10*	WBGene00000033	185248
173482_s_at	2.96507	up	1.56807	abu-11*	WBGene00000034	173404
172323_x_at	6.39367	up	2.67664	abu-6*	WBGene00000029	178903
172321_x_at	4.84522	up	2.27656	abu-7	WBGene00000030	178904
172587_x_at	4.95913	up	2.31009	abu-8 *	WBGene00000031	178904
194211_x_at	4.30954	up	2.10753	abu-8 *	WBGene00000031	178904
185654_s_at	2.03238	up	1.02317	abu-9*	WBGene00000032	187749
193635_s_at	2.31687	up	1.21218	acl-2	WBGene00011543	179398
174833_at	2.46321	up	1.30054	acs-1	WBGene00018488	179043
191641_s_at	2.29608	up	1.19917	acs-19	WBGene00007969	175500
AFFX-r2-13514-3_s_at	5.72112	up	2.51630	act-1	WBGene00000064	179533
AFFX-r2-13514-5_x_at	8.63106	up	3.10954	act-1	WBGene00000065	179533
AFFX-Ce_Actin_5_f_at	11.03174	up	3.46359	act-1*	WBGene00000063	
AFFX-Ce_Actin_M_s_at	5.75383	up	2.52452	act-1*	WBGene00000063	
190043_s_at	5.34771	up	2.41892	act-1 *	WBGene00000063	179533
172816_x_at	2.74062	up	1.45450	act-2*	WBGene00000063	179533
189551_s_at	2.20945	up	1.14369	act-5	WBGene00000067	176793
**191329_s_at**	**2.12977**	**up**	**1.09070**	**air-1***	**WBGene00000098**	**179202**
189114_at	2.16346	up	1.11334	ampd-1	WBGene00016415	173891
186867_s_at	2.04306	up	1.03073	anat-1	WBGene00015938	177439
174791_s_at	2.06205	up	1.04408	anc-1	WBGene00000140	172034
173676_s_at	2.34910	up	1.23211	apy-1	WBGene00017244	181019

189807_at	3.36129	up	1.74902	aqp-7	WBGene00000175	180589
192156_at	2.78453	up	1.47743	arf-3*	WBGene00000183	177595
173972_s_at	2.53520	up	1.34210	art-1	WBGene00000198	174140
173989_s_at	2.32190	up	1.21531	asp-6*	WBGene00000219	179209
189086_s_at	3.07307	up	1.61968	atn-1	WBGene00018488	179709
188137_s_at	2.20591	up	1.14138	atp-3	WBGene00000230	172195
193256_at	2.07521	up	1.05326	B0393.3	WBGene00007168	175630
187506_s_at	2.49310	up	1.31794	B0416.5	WBGene00015181	181158
187297_s_at	2.15766	up	1.10947	B0495.7	WBGene00015206	174245
175994_s_at	3.12701	up	1.64478	bath-42	WBGene00016803	176152
174152_s_at	2.25633	up	1.17398	bli-1	WBGene00000251	174653
188391_at	2.53678	up	1.34300	bli-2	WBGene00000252	191611
191062_s_at	2.00611	up	1.00440	bli-4	WBGene00000254	172333
184599_at	2.39537	up	1.26025	C01B10.6	WBGene00015280	177432
172353_x_at	2.52659	up	1.33719	C02E7.6	WBGene00015339	178885
172325_x_at	2.49361	up	1.31824	C02E7.7	WBGene00015340	178884
183211_s_at	4.27528	up	2.09602	C04F12.7	WBGene00007301	172819
177577_s_at	2.68114	up	1.42284	C04G2.8	WBGene00007307	177877
**187292_s_at**	**2.16427**	**up**	**1.11388**	**C05C10.5**	**WBGene00007332**	**174551**
177934_s_at	2.06550	up	1.04649	C06A1.6	WBGene00007356	174625
179164_at	2.01153	up	1.00830	C06G8.1	WBGene00007384	177972
188162_at	2.66530	up	1.41430	C09H5.2	WBGene00019875	179189
188163_s_at	2.40738	up	1.26747	C10G11.8	WBGene00015688	179189
186105_s_at	3.72186	up	1.89602	C10G11.9	WBGene00015689	172293
190329_at	2.83003	up	1.50081	C11E4.1	WBGene00007516	181178
184411_at	2.11154	up	1.07829	C12D12.1	WBGene00015713	180642
191943_at	2.01161	up	1.00835	C14C10.1	WBGene00007584	182608
183526_at	2.42174	up	1.27604	C14C6.5*	WBGene00015759	178574
184320_at	4.07984	up	2.02851	C14F11.4	WBGene00015780	182619
181473_s_at	2.87685	up	1.52449	C15C6.2*	WBGene00007601	173099
190619_at	2.19918	up	1.13697	C15C8.3	WBGene00007605	182635
**187346_at**	**2.50465**	**up**	**1.32461**	**C16C10.3***	**WBGene00007624**	**175535**
187412_at	2.32412	up	1.21668	C18H9.6*	WBGene00016004	182809
189758_s_at	2.17872	up	1.12348	C25A1.4*	WBGene00007706	172881
186257_s_at	3.01128	up	1.59038	C29H12.2	WBGene00016235	174022
**175939_s_at**	**2.24848**	**up**	**1.16895**	**C30A5.3***	**WBGene00016238**	**176158**
180909_at	2.28174	up	1.19013	C32H11.4	WBGene00007867	178244
180997_at	3.02588	up	1.59735	C34D4.3	WBGene00016399	177488
181291_at	2.53047	up	1.33941	C34D4.4	WBGene00016400	183205

183115_at	2.96271	up	1.56692	C34H4.2	WBGene00016425	183220
179035_s_at	2.47370	up	1.30667	C35A5.8	WBGene00007952	179467
181840_s_at	2.00966	up	1.00695	C35C5.3*	WBGene00007955	181343
182691_s_at	2.47417	up	1.30694	C36A4.5	WBGene00007966	172918
180231_at	2.49613	up	1.31969	C37C3.9*	WBGene00016501	179164
192234_s_at	2.91803	up	1.54500	C38C10.2	WBGene00008000	176317
189054_at	2.48832	up	1.31517	C39H7.1	WBGene00016541	177341
180540_at	3.27268	up	1.71047	C42D4.3	WBGene00016596	177492
183193_at	2.15666	up	1.10880	C46G7.2	WBGene00016722	177367
183956_at	2.00333	up	1.00240	C48E7.1	WBGene00016749	172322
179858_at	2.35668	up	1.23675	C49C8.5	WBGene00016769	177668
178517_at	2.39023	up	1.25715	C49F8.3	WBGene00008215	181459
192995_at	2.13310	up	1.09295	C50F7.4*	WBGene00016844	177555
179176_at	2.65226	up	1.40722	C52G5.2*	WBGene00008260	181420
180628_at	3.39175	up	1.76203	C53B7.3*	WBGene00016894	180957
180064_at	2.15915	up	1.11046	C54D2.1*	WBGene00016915	183776
180685_at	2.07157	up	1.05073	C54E4.2*	WBGene00016918	177010
190667_s_at	2.85074	up	1.51134	calu-1	WBGene00019760	180769
193984_s_at	2.03876	up	1.02769	cdc-48.1	WBGene00007352	174624
**188316_s_at**	**2.12480**	**up**	**1.08733**	**cdl-1**	**WBGene00000411**	**174659**
**187802_at**	**2.51161**	**up**	**1.32861**	**cgh-1**	**WBGene00000479**	**176061**
**193886_at**	**3.29850**	**up**	**1.72181**	**cks-1***	**WBGene00001051**	**177658**
184002_at	2.85086	up	1.51140	clc-1	WBGene00000522	181293
186278_at	2.48240	up	1.31173	clec-150	WBGene00019914	175261
184035_at	2.10788	up	1.07579	clec-265	WBGene00019738	180590
189915_s_at	2.14301	up	1.09964	clec-41	WBGene00007153	179797
192509_at	2.16135	up	1.11193	clec-60	WBGene00014046	191384
176743_s_at	2.02588	up	1.01855	clec-67	WBGene00018971	177151
188109_s_at	2.64431	up	1.40289	clec-87	WBGene00007709	172885
189797_s_at	2.82274	up	1.49709	col-104	WBGene00000678	176947
173235_at	3.03217	up	1.60035	col-109*	WBGene00000683	176988
172879_x_at	2.07574	up	1.05363	col-12	WBGene00000601	179452
172701_x_at	2.62046	up	1.38982	col-125	WBGene00000699	178029
172878_x_at	2.24083	up	1.16403	col-13	WBGene00000601	179452
188364_s_at	3.33723	up	1.73865	col-130	WBGene00000704	178193
183666_at	3.33237	up	1.73655	col-138	WBGene00000711	183722
188500_at	2.71134	up	1.43900	col-142	WBGene00000715	188514
172475_x_at	3.89070	up	1.96003	col-145	WBGene00000718	179297
171735_x_at	2.07681	up	1.05437	col-147	WBGene00000720	179351

188350_at	2.12146	up	1.08506	col-149	WBGene00000722	179431
172700_x_at	2.21459	up	1.14704	col-157	WBGene00000730	188426
172746_x_at	3.26193	up	1.70572	col-166	WBGene00000742	180902
172735_x_at	2.97588	up	1.57332	col-167	WBGene00000740	180907
189482_s_at	2.40100	up	1.26364	col-175	WBGene00000748	181154
188475_at	2.79251	up	1.48156	col-180	WBGene00000753	181356
189968_s_at	2.75721	up	1.46321	col-38	WBGene00000615	174370
190584_at	2.53568	up	1.34237	col-39	WBGene00000616	174651
190134_s_at	2.19661	up	1.13528	col-41*****	WBGene00000618	181610
188747_at	4.18272	up	2.06444	col-48	WBGene00000625	171873
189762_at	2.50837	up	1.32675	col-49	WBGene00000626	187239
173767_s_at	2.73004	up	1.44892	col-62	WBGene00000596	172494
189099_at	4.04222	up	2.01515	col-77	WBGene00000653	174336
190093_at	2.48279	up	1.31196	col-88	WBGene00000663	175170
177032_at	3.38155	up	1.75769	col-91	WBGene00000666	184269
188473_s_at	3.04046	up	1.60429	col-97	WBGene00000672	176721
188113_s_at	2.16606	up	1.11508	cox-15	WBGene00011526	174712
**187525_s_at**	**2.46684**	**up**	**1.30267**	**cpg-2**	**WBGene00015102**	**175991**
192114_s_at	2.36435	up	1.24145	cth-2	WBGene00022856	174155
178608_s_at	2.09800	up	1.06901	cup-2*****	WBGene00000843	172915
172706_x_at	3.45465	up	1.78854	cuticlin (F53F1.4)	WBGene00009982	179822
172741_x_at	3.75105	up	1.90729	Cuticlin (F41F3.3)	WBGene00018297	178856
**172738_x_at**	**2.95630**	**up**	**1.56379**	**cyb-2.1**	**WBGene00000866**	**177994**
**172615_x_at**	**2.75252**	**up**	**1.46075**	**cyb-2.2**	**WBGene00000867**	**171993**
**194213_x_at**	**3.38700**	**up**	**1.76001**	**cyb-2.2**	**WBGene00000867**	**171993**
**188916_s_at**	**2.80402**	**up**	**1.48750**	**cyb-3**	**WBGene00000868**	**180040**
178786_s_at	2.57252	up	1.36318	D1081.7	WBGene00008385	172638
177920_at	3.02031	up	1.59470	D1086.3	WBGene00008390	179907
187176_s_at	3.21072	up	1.68289	D2096.6	WBGene00017073	177633
188215_at	2.97808	up	1.57438	daf-18*	WBGene00000913	176869
**188460_s_at**	**2.19884**	**up**	**1.13674**	**daz-1***	**WBGene00000935**	**173931**
177978_at	2.24693	up	1.16796	dct-18	WBGene00010266	174934
188558_s_at	3.51117	up	1.81195	dim-1	WBGene00001000	181062
173606_s_at	2.11260	up	1.07902	dlc-1	WBGene00001005	175922
189606_s_at	2.08120	up	1.05742	dnj-19	WBGene00001037	3565862
177463_at	2.02057	up	1.01476	dod-19	WBGene00022644	178564
172218_x_at	2.80608	up	1.48856	dod-6*	WBGene00011869	176433
187205_s_at	2.04749	up	1.03386	dpff-1*	WBGene00016200	175832
190360_s_at	3.00888	up	1.58923	dpy-5	WBGene00001067	172197

180747_s_at	2.24597	up	1.16734	dsc-4	WBGene00001099	176852
177729_at	3.74121	up	1.90351	E01G4.6	WBGene00008448	174990
193298_s_at	2.73133	up	1.44961	eat-6	WBGene00001137	179796
193064_at	2.03858	up	1.02756	eef-1B.2	WBGene00012768	178419
190244_s_at	2.22306	up	1.15254	egl-21	WBGene00001189	177940
191276_s_at	3.65283	up	1.86901	elo-5	WBGene00001243	177320
173725_s_at	2.61705	up	1.38794	elo-6	WBGene00001244	177321
192676_s_at	2.17146	up	1.11867	emb-5	WBGene00001259	175621
190353_s_at	3.43813	up	1.78163	emb-9	WBGene00001263	176314
AFFX-r2-3026-5_at	2.41198	up	1.27022	enol-1	WBGene00011884	174423
177953_s_at	3.30550	up	1.72487	ent-2*	WBGene00010701	181648
182964_s_at	2.01310	up	1.00942	ergo-1	WBGene00019971	178602
189677_s_at	2.80319	up	1.48707	erm-1	WBGene00001333	172174
192910_s_at	2.72480	up	1.44615	F01G4.6	WBGene00008505	178020
188758_at	2.84345	up	1.50764	F07H5.8	WBGene00008559	174407
183194_s_at	2.21621	up	1.14809	F08F3.6	WBGene00017263	178925
183159_at	2.02530	up	1.01814	F09F9.2	WBGene00017307	184263
191454_at	2.11365	up	1.07973	F10G8.7	WBGene00008665	172867
**181081_at**	**2.66038**	**up**	**1.41163**	**F11A10.5****	**WBGene00008686**	**178152**
179439_s_at	2.76979	up	1.46978	F11E6.3	WBGene00008707	178533
**188584_s_at**	**2.41958**	**up**	**1.27476**	**F14B4.2**	**WBGene00008780**	**174333**
176786_at	2.21912	up	1.14999	F14H12.3	WBGene00017471	180709
178625_s_at	3.43432	up	1.78003	F14H3.6	WBGene00008825	180077
186492_s_at	3.02105	up	1.59505	F15E11.1	WBGene00017490	178710
186519_at	2.92835	up	1.55009	F15E11.12	WBGene00017498	178712
177131_s_at	2.15912	up	1.11044	F15E11.13	WBGene00017499	178709
181257_at	2.57681	up	1.36559	F17C11.2	WBGene00008914	184614
**175074_s_at**	**2.35833**	**up**	**1.23777**	**F18A1.7**	**WBGene00017548**	**174237**
178164_at	2.10092	up	1.07102	F21H7.5	WBGene00009031	180090
187770_s_at	2.35600	up	1.23634	F25B5.3	WBGene00017775	175843
194218_x_at	2.20529	up	1.14097	F25D7.4	WBGene00009113	172918
172775_x_at	2.16286	up	1.11294	F25D7.4	WBGene00009113	172918
181410_s_at	2.82273	up	1.49709	F26G1.5	WBGene00017841	173823
183734_at	2.32308	up	1.21604	F27C1.1	WBGene00017851	172198
181937_s_at	2.08541	up	1.06033	F27C1.2	WBGene00017852	172196
187429_s_at	2.12729	up	1.08902	F31E3.4*	WBGene00017951	175974
189761_s_at	2.29851	up	1.20070	F32B5.1*	WBGene00017975	173236
192078_at	2.27793	up	1.18772	F32B6.2	WBGene00009319	177841
185764_s_at	2.10595	up	1.07447	F33D4.6*	WBGene00017998	177552

176681_at	2.30441	up	1.20440	F35B3.4	WBGene00018031	185255
185153_s_at	2.08501	up	1.06005	F35E12.10	WBGene00009434	179868
179695_at	2.84233	up	1.50707	F35E12.5	WBGene00009429	185307
185480_s_at	2.38707	up	1.25524	F36H12.8	WBGene00018122	177276
187476_at	2.02052	up	1.01473	F37A4.1*	WBGene00018131	175948
184083_s_at	2.14928	up	1.10386	F37B12.3*	WBGene00009505	174439
181941_s_at	2.23337	up	1.15922	F37C4.5*	WBGene00018145	177146
173727_s_at	2.88791	up	1.53002	F40F4.6	WBGene00018237	180613
185537_at	3.39274	up	1.76245	F41E6.11	WBGene00018292	179245
186757_s_at	2.71824	up	1.44267	F42A8.1	WBGene00009626	174481
190977_s_at	2.28372	up	1.19138	F42D1.2*	WBGene00009628	181574
190958_s_at	2.36332	up	1.24081	F44E5.4*	WBGene00009691	174805
182163_s_at	2.00320	up	1.00231	F44E7.5	WBGene00018427	178967
186801_s_at	2.36351	up	1.24093	F45D11.14	WBGene00018459	173480
184276_at	2.03535	up	1.02528	F46F11.6	WBGene00018509	172218
179187_s_at	2.69422	up	1.42987	F46F2.3	WBGene00009787	181619
189706_s_at	2.18530	up	1.12783	F46H5.3	WBGene00018519	180986
191301_s_at	2.06672	up	1.04735	F47B10.2	WBGene00009813	181279
190419_s_at	2.05987	up	1.04255	F47G9.1*	WBGene00009829	179567
183020_at	2.03937	up	1.02812	F48G7.8	WBGene00018619	185996
186468_s_at	2.50372	up	1.32407	F49C12.12	WBGene00009881	177755
181311_s_at	2.24230	up	1.16498	F49C12.9*	WBGene00009878	177752
178587_s_at	2.29119	up	1.19610	F52A8.1	WBGene00009915	172482
182970_at	2.89645	up	1.53429	F55G11.4	WBGene00010124	178248
180707_at	2.34325	up	1.22851	F55H12.4	WBGene00010135	186344
182717_at	2.27705	up	1.18717	F56B3.6	WBGene00018930	176906
188635_at	2.81536	up	1.49332	F58E6.13	WBGene00077697	6418750
177974_s_at	2.23964	up	1.16326	F58G1.1	WBGene00010263	174932
172190_x_at	2.65311	up	1.40768	F59F4.2*	WBGene00010337	181669
183396_s_at	2.99084	up	1.58055	farl-11	WBGene00017349	174165
192186_at	2.05141	up	1.03661	fat-2*	WBGene00001394	178293
179338_at	2.20772	up	1.14256	frm-10	WBGene00001496	179828
193200_s_at	2.37643	up	1.24880	gale-1	WBGene00008132	173171
190626_s_at	5.58029	up	2.48034	gdh-1*	WBGene00014095	178130
188046_s_at	16.28070	up	4.02509	gfi-1	WBGene00001581	179022
190303_s_at	2.06318	up	1.04487	gly-8	WBGene00001633	176595
AFFXCe_Gapdh_M_s_at	3.14864	up	1.65473	gpd-3	WBGene00001685	
185646_at	2.73173	up	1.44982	grd-14	WBGene00001703	187934
181972_at	2.22868	up	1.15619	grd-3	WBGene00001692	177109

180979_at	2.13624	up	1.09508	grd-5	WBGene00001694	179244
188028_at	2.71935	up	1.44326	grd-6	WBGene00001695	179305
186927_s_at	2.31360	up	1.21014	grl-16*	WBGene00001725	171649
182143_s_at	2.34199	up	1.22774	grl-4	WBGene00001713	3564844
173004_s_at	2.64340	up	1.40239	grl-7	WBGene00001716	179569
187085_s_at	2.58184	up	1.36840	gst-10*	WBGene00001758	178725
190852_at	2.12030	up	1.08427	gst-13	WBGene00001761	188915
**181288_at**	**2.24946**	**up**	**1.16958**	**H02I12.5***	**WBGene00010353**	**178064**
173083_s_at	2.45459	up	1.29548	H10E21.4	WBGene00019184	175168
184288_at	2.05102	up	1.03634	H14A12.3*	WBGene00019196	176058
185593_at	2.54010	up	1.34488	H23N18.5	WBGene00019236	186770
179787_at	2.44103	up	1.28749	H36L18.2	WBGene00010424	181411
174393_at	2.59412	up	1.37524	H40L08.2	WBGene00014793	
173124_s_at	2.06670	up	1.04733	H42K12.3	WBGene00019272	
AFFX-r2-3487-5_s_at	2.07932	up	1.05611	hel-1*	WBGene00001840	180474
**188740_at**	**2.01638**	**up**	**1.01177**	**hil-5**	**WBGene00001856**	**172751**
**183760_s_at**	**2.43204**	**up**	**1.28216**	**him-1**	**WBGene00001860**	**172242**
**188001_s_at**	**2.15270**	**up**	**1.10614**	**him-17**	**WBGene00001874**	**172116**
190225_s_at	2.26938	up	1.18230	his-24	WBGene00001898	179800
172891_x_at	2.14184	up	1.09885	his-48	WBGene00001922	181545
172758_x_at	2.32038	up	1.21436	his-48	WBGene00001922	178049
172702_x_at	2.08274	up	1.05848	his-62	WBGene00001936	178049
184409_s_at	2.56318	up	1.35793	hmg-1.1*	WBGene00001971	186326
176044_at	3.38532	up	1.75929	hp0-6	WBGene00021518	175081
173626_s_at	3.60333	up	1.84933	hpo-32*	WBGene00009259	181340
188444_at	2.30207	up	1.20293	hsp-16.41	WBGene00002018	176965
172901_x_at	2.22904	up	1.15642	hsp-16.48	WBGene00002019	178660
190900_s_at	2.04685	up	1.03341	hsp-25	WBGene00002023	179287
**189914_s_at**	**2.07102**	**up**	**1.05034**	**htz-1**	**WBGene00019947**	**180872**
177684_s_at	2.49077	up	1.31659	icl-1	WBGene00001564	177212
190796_s_at	2.80481	up	1.48790	idh-1*	WBGene00010317	177775
193131_at	2.05506	up	1.03918	ifa-1	WBGene00002050	177730
192118_s_at	2.02219	up	1.01592	ifb-2	WBGene00002054	181316
189324_s_at	2.00638	up	1.00460	ile-1	WBGene00002070	173973
**192178_s_at**	**2.69565**	**up**	**1.43063**	**ima-1***	**WBGene00002072**	**172799**
**192711_at**	**2.16085**	**up**	**1.11160**	**ima-2**	**WBGene00002073**	**179555**
193731_s_at	3.36484	up	1.75054	immt-1	WBGene00020511	172329
173547_s_at	2.07086	up	1.05023	imp-1	WBGene00007979	180566
189792_s_at	2.15281	up	1.10622	imp-2*	WBGene00011481	172684

192422_s_at	2.05060	up	1.03605	ina-1	WBGene00002081	178013
188535_s_at	2.28230	up	1.19049	inf-1	WBGene00002083	176296
192240_s_at	5.17754	up	2.37227	inf-1	WBGene00018997	175966
171811_s_at	2.17749	up	1.12267	K01D12.7*	WBGene00010466	175966
187787_at	2.11033	up	1.07747	k02D10.1	WBGene00019301	176232
193627_at	3.17719	up	1.66775	K02F2.2	WBGene00019322	179698
179892_at	2.84474	up	1.50830	K06A5.2	WBGene00019430	172408
173922_s_at	2.11228	up	1.07880	K07H8.2	WBGene00019504	259316
178927_at	2.78953	up	1.48002	K08D8.5*	WBGene00010659	177623
177700_at	4.66185	up	2.22090	K08D8.6	WBGene00010660	178243
177701_s_at	2.23729	up	1.16175	K08D8.6	WBGene00010660	178241
194129_s_at	2.66204	up	1.41253	K08E3.5	WBGene00010665	178241
189319_at	2.46684	up	1.30267	K08F4.1	WBGene00010676	176814
187580_s_at	2.17728	up	1.12253	K09G1.1	WBGene00010727	177879
190815_s_at	2.71363	up	1.44023	K10C2.1	WBGene00019617	179346
189595_s_at	2.64570	up	1.40365	K10C2.3	WBGene00019619	180915
192003_s_at	2.21892	up	1.14986	K11G12.5	WBGene00019656	180917
**186482_s_at**	**2.15586**	**up**	**1.10826**	**kca-1***	**WBGene00015698**	**180940**
**187874_at**	**2.12927**	**up**	**1.09036**	**kin-3**	**WBGene00002191**	**172090**
**188638_s_at**	**2.54340**	**up**	**1.34676**	**klp-15**	**WBGene00002225**	**172978**
193446_s_at	3.76413	up	1.91232	klp-16	WBGene00002226	172213
194001_s_at	2.75596	up	1.46256	lec-1*	WBGene00002264	172784
192341_s_at	2.41289	up	1.27076	let-75*	WBGene00002348	174964
**183157_s_at**	**3.00312**	**up**	**1.58646**	**lin-37**	**WBGene00003022**	**172471**
**177366_s_at**	**2.12442**	**up**	**1.08707**	**lin-54**	**WBGene00003037**	**175986**
176605_s_at	2.22468	up	1.15360	lir-1	WBGene00003044	178280
193082_s_at	2.37902	up	1.25037	lmn-1	WBGene00003052	174240
187660_s_at	2.13794	up	1.09622	lmp-1	WBGene00003053	172687
188368_at	2.23830	up	1.16241	lon-3*	WBGene00003057	180912
AFFX-r2-18565-5_x_at	4.44784	up	2.15310	mai-1*	WBGene00003124	179673
AFFX-Ce_Gapdh_5_s_at	4.60056	up	2.20181	mai-1	WBGene00003124	174350
177535_s_at	2.09050	up	1.06385	MBOA-6	WBGene00020115	176016
**193664_s_at**	**2.07894**	**up**	**1.05585**	**mcm-3**	**WBGene00003155**	**3565232**
**173443_s_at**	**2.46743**	**up**	**1.30301**	**mei-2**	**WBGene00003184**	**180010**
**193943_at**	**2.06826**	**up**	**1.04841**	**mex-5**	**WBGene00003230**	**172374**
**192002_at**	**2.17034**	**up**	**1.11792**	**mex-6**	**WBGene00003231**	**178296**
190057_s_at	3.28763	up	1.71705	mig-6	WBGene00003242	179224
185322_s_at	2.39461	up	1.25979	mlp-1	WBGene00003375	174504
187408_s_at	2.15781	up	1.10957	mogs-1*	WBGene00008775	177998

**179898_s_at**	**2.06763**	**up**	**1.04798**	**mrg-1**	**WBGene00003406**	**175847**
193046_at	3.12463	up	1.64369	mrp-4	WBGene00003410	176702
183958_s_at	2.36102	up	1.23941	MTCH-1	WBGene00018395	174207
173556_s_at	2.21505	up	1.14734	mthf-1	WBGene00015512	174254
173285_s_at	2.24516	up	1.16682	ncx-1	WBGene00003566	181202
187929_s_at	2.46059	up	1.29900	ndg-4	WBGene00003577	180318
191753_at	2.68600	up	1.42546	NEP-17	WBGene00010070	174996
188026_at	2.13004	up	1.09088	nex-1	WBGene00003588	175582
184985_s_at	2.48704	up	1.31443	nlp-40*	WBGene00022276	175714
**184943_s_at**	**2.99886**	**up**	**1.58441**	**nos-2***	**WBGene00003784**	**171591**
**188236_s_at**	**2.23052**	**up**	**1.15738**	**nos-2***	**WBGene00003784**	**174158**
191157_s_at	2.70489	up	1.43557	npp-12	WBGene00003798	174158
179133_at	2.04446	up	1.03172	NRA-1	WBGene00011488	176788
185454_s_at	2.78433	up	1.47733	nspb-1	WBGene00018162	172348
172469_x_at	3.13336	up	1.64771	nspb-10	WBGene00007246	185443
172474_x_at	2.00632	up	1.00455	nspb-11*	WBGene00009398	182088
172344_x_at	2.33607	up	1.22408	nspb-12	WBGene00017305	185265
172595_x_at	2.61925	up	1.38915	nspb-2	WBGene00018162	184261
172417_x_at	2.94094	up	1.55628	nspb-3	WBGene00018162	185443
185645_s_at	2.04218	up	1.03011	nstp-2*	WBGene00017480	177424
187818_s_at	2.20317	up	1.13958	nurf-1	WBGene00009180	185443
**189867_s_at**	**2.38188**	**up**	**1.25210**	**oma-1***	**WBGene00003864**	**175098**
**189988_s_at**	**2.96016**	**up**	**1.56567**	**oma-2**	**WBGene00003865**	**177703**
193068_at	3.20311	up	1.67947	opt-2	WBGene00003877	179183
191389_s_at	2.11166	up	1.07838	ost-1	WBGene00003893	180919
194118_s_at	2.07389	up	1.05234	pab-1*	WBGene00003902	176931
173882_s_at	2.08967	up	1.06328	pab-2	VWBGene00003903	172920
**177834_s_at**	**3.54755**	**up**	**1.82682**	**patr-1***	**WBGene00009661**	**181473**
191298_s_at	2.45518	up	1.29583	pcp-2	WBGene00003957	174808
192032_s_at	2.00937	up	1.00675	pdi-6	WBGene00015168	177741
176873_s_at	3.10960	up	1.63673	perm-3*	WBGene00022776	180974
192285_at	2.07257	up	1.05142	pfd-3	WBGene00006889	174191
182024_at	2.17601	up	1.12168	pfn-2*	WBGene00003990	173141
190248_s_at	2.79126	up	1.48092	pgp-14	WBGene00004008	180808
180962_s_at	2.21761	up	1.14901	pgrn-1	WBGene00011936	178777
194228_x_at	2.05370	up	1.03823	pgrn-1	WBGene00011936	173054
182713_s_at	2.05814	up	1.04134	phat-3	WBGene00016781	178809
182573_at	2.92596	up	1.54891	phat-4	WBGene00020237	181416
181762_s_at	2.80537	up	1.48819	pisy-1	WBGene00012897	178812

**191208_s_at**	**3.00971**	**up**	**1.58963**	**plk-3**	**WBGene00004044**	**174902**
188111_at	2.59410	up	1.37524	plp-1	WBGene00004046	266900
**192079_s_at**	**3.77060**	**up**	**1.91479**	**pos-1**	**WBGene00004078**	**177540**
191504_at	2.06631	up	1.04706	pps-1	WBGene00004091	179162
187280_s_at	2.28044	up	1.18931	ppw-2	WBGene00004094	177893
185070_s_at	2.18892	up	1.13022	pqn-22	WBGene00004112	172152
180505_at	2.86081	up	1.51642	pqn-32	WBGene00004120	177369
183975_s_at	2.02240	up	1.01607	pqn-46	WBGene00004133	174955
172360_x_at	7.77992	up	2.95976	pqn-5	WBGene00004099	175970
183344_at	5.70779	up	2.51293	pqn-54	WBGene00004139	182136
179437_s_at	3.36556	up	1.75085	pqn-74*	WBGene00004156	187728
172443_x_at	2.49704	up	1.32022	pqn-91	WBGene00004170	178294
183577_at	3.41566	up	1.77217	pqn-95*	WBGene00004174	190671
174071_at	3.95570	up	1.98393	pqn-95*	WBGene00004174	174464
**177402_s_at**	**2.61558**	**up**	**1.38713**	**prg-1**	**WBGene00004178**	**174464**
179616_at	2.04505	up	1.03213	prx-14	WBGene00004199	172515
175239_at	3.66044	up	1.87202	pud-3*	WBGene00017501	178711
**194076_s_at**	**3.01185**	**up**	**1.59065**	**puf-3***	**WBGene00004239**	**178026**
**188144_s_at**	**2.28995**	**up**	**1.19531**	**puf-8**	**WBGene00004244**	**178320**
193700_s_at	2.14681	up	1.10220	pxf-1	WBGene00004254	174197
189418_s_at	2.08112	up	1.05736	PYK-1	WBGene00009126	172744
193523_at	2.04895	up	1.03488	qdpr-1*	WBGene00011398	177894
189080_at	2.12397	up	1.08676	R02D3.1	WBGene00019819	176842
183661_at	2.36587	up	1.24237	R02F2.1	WBGene00019831	176762
189696_s_at	2.13310	up	1.09295	R05G6.7	WBGene00019900	175757
191828_at	2.36316	up	1.24071	R08E3.1	WBGene00019957	177524
182747_at	2.49547	up	1.31931	R08E5.3	WBGene00019963	180758
192726_at	2.04977	up	1.03546	R11H6.2	WBGene00011250	178795
190616_at	2.35689	up	1.23688	R12C12.1*	WBGene00020022	179964
172547_x_at	3.26982	up	1.70921	R12E2.14	WBGene00020039	174012
171795_x_at	2.70173	up	1.43388	R12E2.14	WBGene00020039	172001
172497_x_at	4.60786	up	2.20410	R12E2.15	WBGene00020040	172001
172346_x_at	5.24090	up	2.38982	R12E2.7	WBGene00020033	172000
192383_s_at	3.42270	up	1.77513	R151.2	WBGene00020107	172002
189752_s_at	2.06616	up	1.04695	R53.2	WBGene00011272	175339
189793_at	2.80140	up	1.48615	rab-5*	WBGene00004268	174553
190167_s_at	2.44436	up	1.28946	rab-6.1	WBGene00004269	172755
191142_s_at	2.36470	up	1.24166	rad-23	WBGene00013924	176275
188311_s_at	6.73507	up	2.75169	ram-2	WBGene00004300	174785

189729_s_at	2.81688	up	1.49410	ran-1*	WBGene00004302	174687
192220_at	2.27523	up	1.18601	rer-1	WBGene00009783	176503
190792_s_at	2.96265	up	1.56689	ret-1	WBGene00004336	174410
193165_at	2.68164	up	1.42312	rhr-1	WBGene00004358	179981
189552_at	2.19476	up	1.13406	ribo-1*	WBGene00020683	177456
179134_s_at	2.04319	up	1.03082	rmd-3	WBGene00007190	178927
190655_at	2.10888	up	1.07648	rnp-1	WBGene00004384	174732
**181020_at**	**2.08841**	**up**	**1.06240**	**rod-1**	**WBGene00018900**	**179672**
188337_at	2.35086	up	1.23319	rol-1	WBGene00004394	177520
192968_s_at	2.29877	up	1.20086	rol-8	WBGene00004398	174857
**190813_at**	**2.45808**	**up**	**1.29753**	**rpa-1**	**WBGene00017546**	**174226**
193674_s_at	2.07018	up	1.04976	rsp-2	WBGene00004699	174238
192547_s_at	2.02236	up	1.01604	sams-4*	WBGene00015540	177354
188971_s_at	3.44806	up	1.78579	sca-1	WBGene00004736	174747
178399_s_at	2.41910	up	1.27447	scc-3	WBGene00004738	176512
173198_s_at	2.16781	up	1.11624	sdhb-1*	WBGene00006433	179749
180256_at	2.68077	up	1.42265	sdz-24	WBGene00019495	174482
186638_s_at	2.22852	up	1.15609	sec-16	WBGene00013985	173457
185913_at	2.03995	up	1.02854	sec-23*	WBGene00004754	176289
192713_s_at	2.17381	up	1.12023	sec-24.1	WBGene00004755	180317
181627_at	2.10770	up	1.07567	sec-6	WBGene00017284	178078
193652_s_at	4.04500	up	2.01614	sec-61	WBGene00013311	173910
174940_at	2.24985	up	1.16983	SKPO-3	WBGene00017968	177324
193777_s_at	2.09274	up	1.06539	skr-1	WBGene00004807	178407
188304_at	2.28808	up	1.19413	smd-1	WBGene00004875	172775
191015_s_at	2.50954	up	1.32742	snb-1	WBGene00004897	173269
192356_s_at	2.07901	up	1.05590	spc-1	WBGene00004951	266648
**192036_at**	**4.17071**	**up**	**2.06029**	**spn-4**	**WBGene00004984**	**180603**
192674_s_at	3.62093	up	1.85636	sqt-1	WBGene00005016	179070
**188115_s_at**	**2.40859**	**up**	**1.26819**	**sqv-7***	**WBGene00005025**	**174731**
190983_at	2.14582	up	1.10153	srs-2*	WBGene00005663	174145
187924_s_at	2.08369	up	1.05914	sss-1	WBGene00006056	177859
**179485_s_at**	**2.09596**	**up**	**1.06761**	**sun-1**	**WBGene00006311**	**177843**
183970_at	2.56546	up	1.35922	T04C12.3	WBGene00011428	179802
177794_at	2.44551	up	1.29014	T05B9.1*	WBGene00011464	179532
192888_s_at	2.13107	up	1.09158	T05D4.1	WBGene00011474	174718
181494_s_at	2.14888	up	1.10359	T07C4.3	WBGene00011560	172803
189458_s_at	2.02657	up	1.01904	T07F10.1	WBGene00011587	176451
191403_s_at	2.78086	up	1.47553	T08G11.1	WBGene00011629	179763

187557_s_at	2.26625	up	1.18031	T12A2.2	WBGene00020437	172705
189733_at	2.07873	up	1.05570	T12A2.8	WBGene00020442	175886
179619_at	2.43740	up	1.28534	T12G3.5	WBGene00011740	175885
182858_at	2.08321	up	1.05881	T15B7.1	WBGene00020516	188457
181714_at	2.84927	up	1.51059	T19B10.2	WBGene00011831	188512
192370_at	2.31709	up	1.21231	T19B4.3	WBGene00020557	179551
181644_at	3.71167	up	1.89207	T21C9.13	WBGene00011898	172232
176676_s_at	2.07760	up	1.05492	T22F3.3	WBGene00020696	179480
183341_at	2.40432	up	1.26563	T23G11.1	WBGene00011968	173054
179304_at	2.59192	up	1.37402	T24F1.2	WBGene00011994	172534
188823_s_at	2.93626	up	1.55398	T25C12.3	WBGene00012018	259727
173774_s_at	2.15379	up	1.10688	T27A8.1	WBGene00013895	171921
179502_at	2.01740	up	1.01250	T28D6.3	WBGene00012123	174727
186127_at	2.37393	up	1.24728	tag-18*	WBGene00006408	176567
190568_at	2.04250	up	1.03033	tag-72	WBGene00006447	180662
189586_s_at	2.22381	up	1.15303	tbb-1*	WBGene00006536	182875
189042_at	2.09348	up	1.06590	tkt-1*	WBGene00008506	176501
185643_s_at	2.60378	up	1.38061	tnt-2	WBGene00006587	177906
176321_s_at	2.84085	up	1.50632	trap-1	WBGene00022122	181119
176353_s_at	2.99136	up	1.58080	trap-3*	WBGene00021420	171865
192151_at	2.55109	up	1.35111	tsn-1	WBGene00006626	177028
190773_s_at	2.27201	up	1.18397	tsp-12	WBGene00006638	173811
191068_at	2.09721	up	1.06847	ttr-14*	WBGene00011460	177890
190473_at	2.60745	up	1.38264	ttr-27	WBGene00011284	181272
173919_s_at	2.16214	up	1.11246	ttr-44	WBGene00008341	179769
191548_s_at	2.02966	up	1.02124	uba-1*	WBGene00006699	179835
173746_s_at	2.04287	up	1.03060	UBQL-1	WBGene00008852	172434
193282_at	2.18463	up	1.12739	uda-1	WBGene00010697	177855
**193326_s_at**	**2.61653**	**up**	**1.38765**	**ufd-2**	**WBGene00006734**	**179395**
173905_s_at	2.08181	up	1.05784	ugt-46	WBGene00015141	174295
191440_at	2.26600	up	1.18015	ugt-47	WBGene00011006	180404
193683_s_at	2.22557	up	1.15417	unc-112*	WBGene00006836	187570
172018_x_at	2.01901	up	1.01365	unc-112*	WBGene00006836	179972
190436_at	4.28773	up	2.10021	unc-15	WBGene00006754	179972
194006_at	2.00147	up	1.00106	unc-25	WBGene00006762	172491
172153_x_at	2.01902	up	1.01366	unc-44	WBGene00006780	176713
**176736_at**	**2.04168**	**up**	**1.02976**	**uri-1**	**WBGene00016944**	**177366**
193697_s_at	3.90605	up	1.96571	vha-1*	WBGene00006910	183841
189302_s_at	3.21966	up	1.68691	vha-10	WBGene00006919	176383

193564_s_at	3.29787	up	1.72153	vha-12	WBGene00006921	172216
193987_s_at	3.68330	up	1.88100	vha-13	WBGene00013025	180692
189591_s_at	2.92140	up	1.54666	vha-15	WBGene00020507	3564970
177070_s_at	2.46908	up	1.30397	vha-16	WBGene00016258	180534
187683_at	2.32334	up	1.21620	vha-17	WBGene00009882	172134
193422_s_at	3.59784	up	1.84713	vha-2*	WBGene00006911	177757
192832_s_at	2.45723	up	1.29703	vha-4	WBGene00006913	187779
173717_s_at	2.29601	up	1.19913	vha-7	WBGene00006916	174272
187561_s_at	2.91470	up	1.54335	vha-8	WBGene00006917	178219
176872_at	2.49483	up	1.31894	vit-3	WBGene00006927	177442
190596_s_at	2.05098	up	1.03632	vps-15	WBGene00014151	180647
191005_at	2.04413	up	1.03149	W02B12.12	WBGene00012207	174827
191006_s_at	2.13260	up	1.09261	W02B12.12	WBGene00012207	174755
192905_s_at	2.50124	up	1.32264	W02F12.5*	WBGene00020950	174755
181867_s_at	2.59086	up	1.37343	W03F8.4	WBGene00020994	179063
**180101_s_at**	**2.04177**	**up**	**1.02982**	**W05F2.3**	**WBGene00021035**	**177293**
191299_s_at	2.21094	up	1.14466	W07G4.4	WBGene00012338	171908
184769_at	4.05770	up	2.02066	W08E12.2	WBGene00021083	179790
172503_x_at	4.96499	up	2.31179	W08E12.3*	WBGene00021084	189291
172545_x_at	4.43934	up	2.15035	W08E12.4*	WBGene00021085	177098
178417_s_at	2.03611	up	1.02581	W09C5.7*	WBGene00012353	189292
177973_at	2.03039	up	1.02175	wago-4	WBGene00010263	174932
192784_at	2.04782	up	1.03409	wdr-5.1	WBGene00006474	171831
190863_at	2.29453	up	1.19820	xbp-1*	WBGene00006959	175474
193650_at	2.57896	up	1.36679	xbx-6	WBGene00009580	175541
176162_at	2.57633	up	1.36532	Y105E8B.5	WBGene00013690	179361
182839_at	2.09079	up	1.06405	Y11D7A.3	WBGene00012428	173323
184279_at	2.07926	up	1.05607	Y17G9B.5	WBGene00021202	177745
177160_at	2.28604	up	1.19285	Y38C1AA.7	WBGene00021398	177244
**179987_at**	**2.18934**	**up**	**1.13049**	**Y39E4B.5**	**WBGene00012716**	**176830**
191219_at	2.05307	up	1.03779	Y41C4A.11	WBGene00012757	176735
182743_at	2.61380	up	1.38615	Y47D3B.6*	WBGene00012942	189814
176471_at	2.09709	up	1.06839	Y47D7A.13	WBGene00021625	176573
175393_at	2.02450	up	1.01756	Y47D7A.13	WBGene00021625	178841
177239_s_at	2.23800	up	1.16221	Y54E10BR.5*	WBGene00021844	171876
187073_at	2.78928	up	1.47990	Y57A10A.23	WBGene00013263	178841
187345_at	2.06958	up	1.04934	Y57E12AL.1	WBGene00021956	174863
181091_at	2.73243	up	1.45019	Y69E1A.2*	WBGene00013474	179093
187891_at	2.04595	up	1.03277	Y71H10B.1	WBGene00022201	177992

175576_s_at	2.84414	up	1.50799	Y75B7AR.1*	WBGene00022287	180573
178069_s_at	2.61506	up	1.38684	ZC247.1	WBGene00013859	178630
181230_at	2.02378	up	1.01705	zip-2	WBGene00019327	172895
184102_s_at	2.67029	up	1.41700	ZK1025.2	WBGene00014182	175240
182104_at	2.62702	up	1.39343	zk1025.3	WBGene00014183	191492
182942_at	2.41681	up	1.27311	zk1058.9	WBGene00014207	175508
183237_s_at	4.77231	up	2.25469	ZK180.5	WBGene00022679	173030
173576_at	2.28066	up	1.18945	ZK180.5	WBGene00022679	177218
180664_s_at	2.10083	up	1.07096	ZK180.6	WBGene00022680	177219
179543_s_at	2.08201	up	1.05798	ZK 265.9*	WBGene00044094	177218
182638_at	2.40699	up	1.26723	ZK418.5	WBGene00022734	3565961
175796_s_at	2.56988	up	1.36170	ZK484.1	WBGene00022748	175987
179401_at	3.03787	up	1.60306	ZK662.2	WBGene00014039	172291
190938_s_at	2.06836	up	1.04848	ZK829.7*	WBGene00014096	181660
189819_s_at	2.03957	up	1.02826	ZK829.9	WBGene00014097	178132
178652_at	2.70029	up	1.43311	ZK84.1	WBGene00022649	178134
177539_at	2.88947	up	1.53080	ZK858.2	WBGene00014116	174008
193893_at	2.18448	up	1.12729	ZK909.3	WBGene00014148	191443
191572_s_at	2.70249	up	1.43429	zyg-9	WBGene00006994	191461

*target previously predicted by protein binding microarray (GROVE *et al.* 2009)

Predicted or known interaction with *daf-18*

GROVE, C. A., F. DE MASI, M. I. BARRASA, D. E. NEWBURGER, M. J. ALKEMA *et al.*, 2009 A multiparameter network reveals extensive divergence between C. elegans bHLH transcription factors. Cell **138:** 314-327.
