# Supplementary material for: HES-Mediated Repression of Pten in Caenorhabditis elegans
Source: G3 (Bethesda). 2015 Oct 4;5(12):2619–28. doi: 10.1534/g3.115.019463 (PMC4683635; doi:10.1534/g3.115.019463)
Supplement: Supporting Information [file supp_g3.115.019463_FigureS1.pdf]

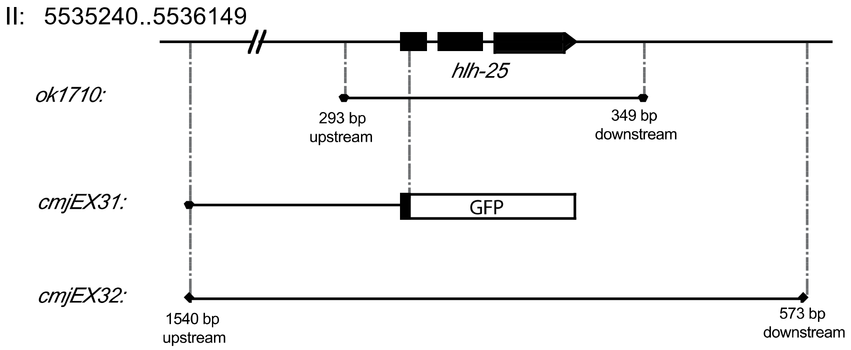

Figure S1. Mutant allele and extrachromosomal arrays for *hlh-25.* The *hlh-25(ok1710)* allele is a 1550 bp deletion of chromosome II that removes the entire coding region for *hlh-25* and additional flanking sequences. The extrachromosomal array *cmjEx31* (II:5533700..5535270::GFP)is a transcriptional fusion that contains 1540 base pairs upstream of the *hlh-25* initiator codon and the first 30 nucleotides of the *hlh-25* coding region. These sequences are fused in-frame to the GFP coding sequences. The extrachromosomal array cmjEx32 (II:5533700..5536722) contains genomic DNA sequences ranging from 1540 bp upstream of the *hlh-25* initiator codon to 573 bp downstream of the *hlh-25* terminator codon.
