# Supplementary material for: HES-Mediated Repression of Pten in Caenorhabditis elegans
Source: G3 (Bethesda). 2015 Oct 4;5(12):2619–28. doi: 10.1534/g3.115.019463 (PMC4683635; doi:10.1534/g3.115.019463)
Supplement: Supporting Information [file supp_g3.115.019463_FigureS2.pdf]

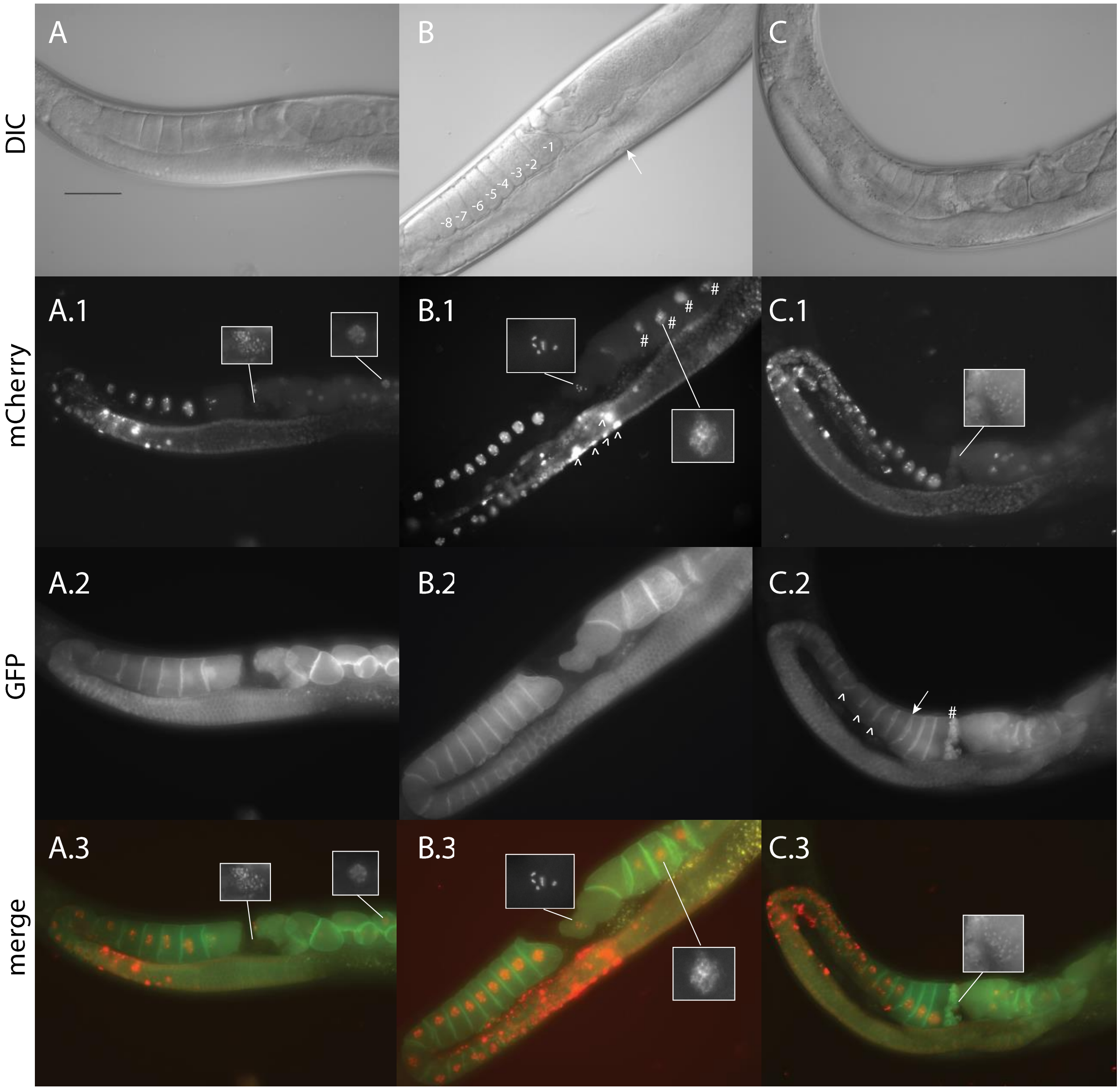

Figure S2. *hlh-25(ok1710)* gonad architecture and oocyte morphology phenotypes. Representative 1) Normarski, 2) mCherry, 3) GFP, and 4) merged mCherry/GFP images of A) wild-type gonad arm seen in Figure 3 and B-C) *hlh-25(ok1710)* gonad arms showing B) increased oocyte number, distal gonad arm constriction (arrow), increased apoptosis (^), abnormal embryonic chromatin (insert, right) and arrested embryos in uterus (#); C) P14,5P_2 debris (#), irregular cellular junctions (arrow), incomplete oocyte compartmentalization (^). Scale bar is 50 µM. In all images ventral is up.
