# Supplementary material for: HES-Mediated Repression of Pten in Caenorhabditis elegans
Source: G3 (Bethesda). 2015 Oct 4;5(12):2619–28. doi: 10.1534/g3.115.019463 (PMC4683635; doi:10.1534/g3.115.019463)
Supplement: Supporting Information [file supp_g3.115.019463_FigureS3.pdf]

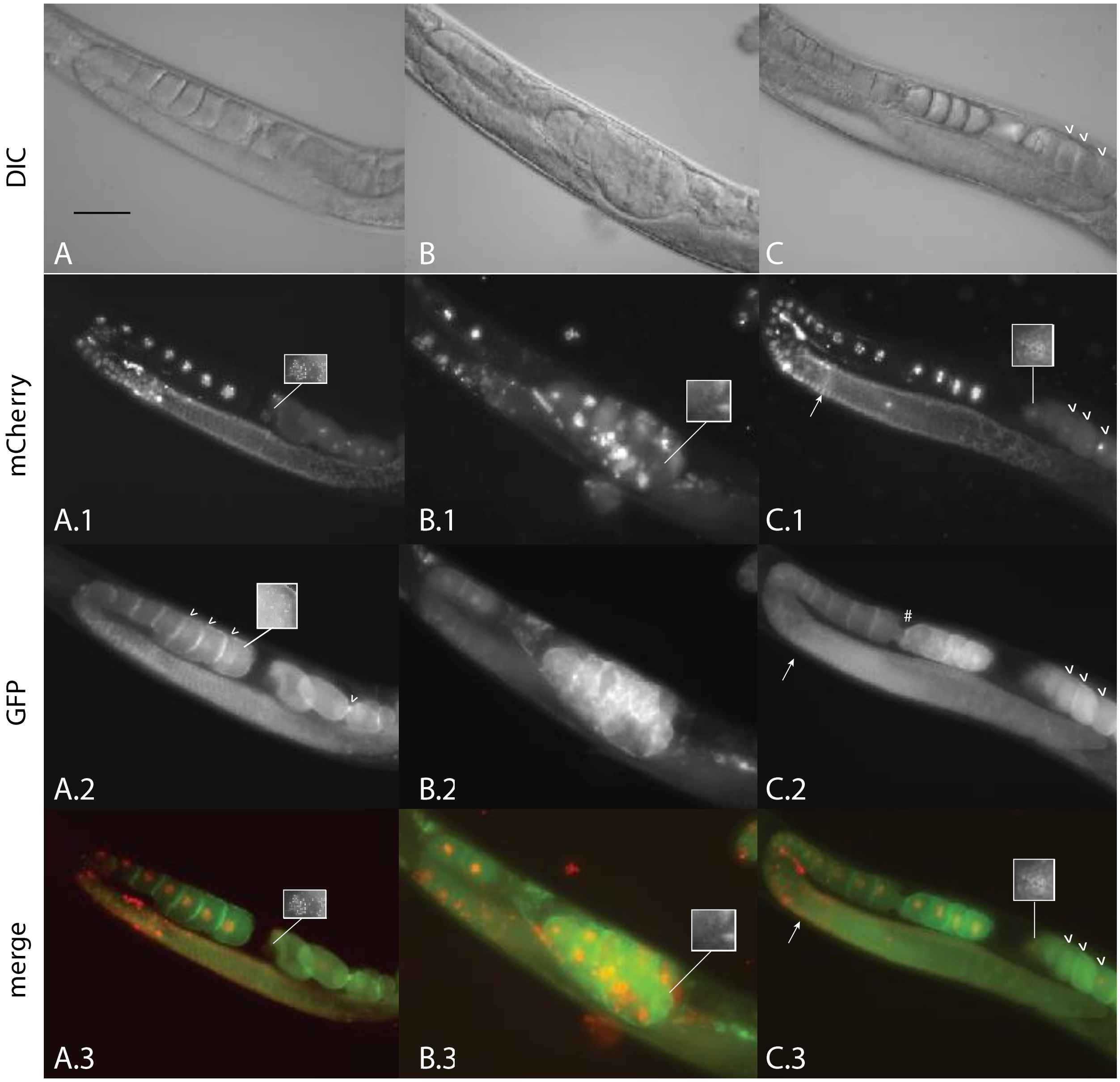

Figure S3. *hlh-25(ok1710)* gonad architecture and oocyte morphology phenotypes. Representative 1) Normarski, 2) mCherry, 3) GFP, and 4) merged mCherry/GFP images of *hlh-25(ok1710)* gonad arms showing A) irregular cellular junctions (^) and cytoplasmic debris in oocytes (insert), B) gonad arm tumor with oocytes containing decondensed chromatin and irregular nuclei. Note position of sperm (inserts) and presumed spermatheca, relative to tumor. Morphology of the distal arm was difficult to capture in image, but showed mild pathfinding defects; C) proximal (white arrow) and distal (#) constrictions of the gonad arm, and abnormal embryos in spermatheca/uterus (^). The proximal constriction is similar to the one resulting in a gonad arm tumor in B. Scale bar is 50 μM. In all images, ventral is up.
