# Supplementary material for: HES-Mediated Repression of Pten in Caenorhabditis elegans
Source: G3 (Bethesda). 2015 Oct 4;5(12):2619–28. doi: 10.1534/g3.115.019463 (PMC4683635; doi:10.1534/g3.115.019463)
Supplement: Supporting Information [file supp_g3.115.019463_FigureS4.pdf]

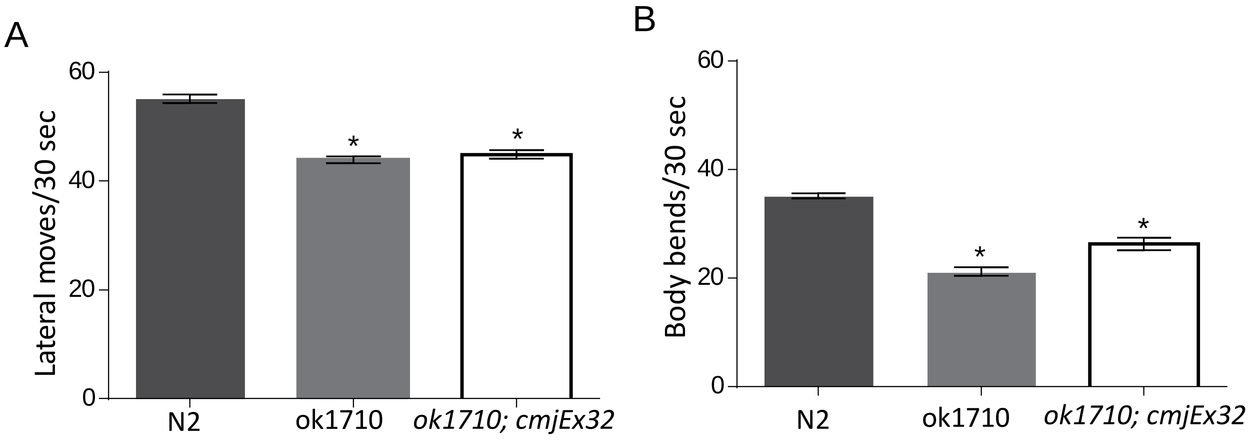

Figure S4. *hlh-25(ok1710)* animals move slower than wild-type. A) Thrashing and B) Locomotion assays comparing *hlh-25(ok1710)* animals (grey bars) to wild-type (black bars) animals. Transgenic expression of cmjEx32 did not rescue the locomotion defects in transgenic *hlh-25(ok1710)* animals (white bars) transgene did not rescue the locomotion defects in null animals. Significance was determined using the student's T test. *,P-value <0.0001.
