# Supplementary material for: HES-Mediated Repression of Pten in Caenorhabditis elegans
Source: G3 (Bethesda). 2015 Oct 4;5(12):2619–28. doi: 10.1534/g3.115.019463 (PMC4683635; doi:10.1534/g3.115.019463)
Supplement: Supporting Information [file supp_g3.115.019463_FigureS5.pdf]

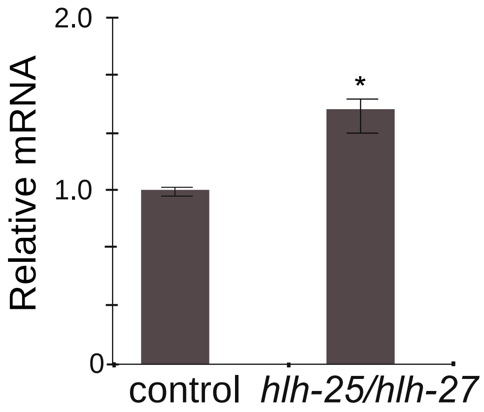

Figure S5. *daf-18* mRNA Levels in Adult Stage Animals. RT-qPCR measurements of *daf-18* mRNA in animals subjected *hlh-25/hlh-27* RNAi relative to animals subjected to control (*unc-55*) RNAi. Error bars represent stand error of the mean (SEM) *P-value <0.05.
