# Supplementary material for: HES-Mediated Repression of Pten in Caenorhabditis elegans
Source: G3 (Bethesda). 2015 Oct 4;5(12):2619–28. doi: 10.1534/g3.115.019463 (PMC4683635; doi:10.1534/g3.115.019463)
Supplement: Supporting Information [file supp_g3.115.019463_TableS1.pdf]

Table S1. Average numbers of live progeny and unfertilized oocytes per day of egg laying per animal.

	Days of Egg-Laying										
	**1**	**2**	**3**	**4**	**5**	**6**	**7**	**8**	**9**	**10**	**TOTAL**
WT brood size	0.0	114.6	132.1	20.9	0.1	0.0	0.0	0.0	0.0	0.0	268
ok1710 brood size	0.9	65.1	93.5	18.6	0.9	0.3	0.1	0.0	0.0	0.0	179
WT oocytes laid	0.0	0.0	8.4	33.1	6.9	0.9	0.0	0.0	0.0	0.0	49
ok1710 oocytes laid	0.0	1.9	11.3	90.9	66.1	37.3	15.3	6.6	2.2	0.0	232
