# Supplementary material for: HES-Mediated Repression of Pten in Caenorhabditis elegans
Source: G3 (Bethesda). 2015 Oct 4;5(12):2619–28. doi: 10.1534/g3.115.019463 (PMC4683635; doi:10.1534/g3.115.019463)
Supplement: Supporting Information [file supp_g3.115.019463_TableS2.pdf]

Table S2. Lifespan Measurements

Strain	Biological Replicate	Total # of Animals to Die/or to be Censored	Median Life Span of All Animals (days)	P-value Log-Rank Test compared to N2 animals
N2	1	88/13	15	
	2	90/10	17	
	3	86/12	15	
	**all**	**264/35**	**16**	
hlh-25	1	93/7	20	< 0.0001
	2	93/7	19	0.0046
	3	96/4	19	< 0.0001
	**all**	**282/18**	**19**	**< 0.0001**
