# Supplementary material for: HES-Mediated Repression of Pten in Caenorhabditis elegans
Source: G3 (Bethesda). 2015 Oct 4;5(12):2619–28. doi: 10.1534/g3.115.019463 (PMC4683635; doi:10.1534/g3.115.019463)
Supplement: Supporting Information [file supp_g3.115.019463_TableS3.pdf]

Table S3 Genes changed by 2.0 fold or greater in *hlh-25(ok1710)* animals.

Genes Downregulated in *hlh-25* Mutants						
Probe Set ID	FCAbsolute	regulation	log2 FC	Gene Symbol	WormBase Gene ID	Entrez Gene
192195_at	3.28185	down	-1.71451	acs-2	WBGene00009221	3565680
174675_at	9.91220	down	-3.30920	acs-2	WBGene00009221	3565680
**175809_at**	**2.54856**	**down**	**-1.34968**	**akt-2***	**WBGene00000103**	**181524**
174489_at	3.19418	down	-1.67544	anc-1	WBGene00000140	172034
179272_at	3.27792	down	-1.71278	C06B3.6	WBGene00007365	182293
186281_s_at	2.94725	down	-1.55937	C08F11.12	WBGene00007459	178333
180306_at	2.03640	down	-1.02602	C10G8.4	WBGene00015683	182503
187800_at	2.35348	down	-1.23479	C16H3.3	WBGene00015868	181787
178441_at	3.74486	down	-1.90491	C34C6.7	WBGene00007919	174393
175482_at	2.40693	down	-1.26719	C34E10.10	WBGene00016411	175715
178672_s_at	2.02000	down	-1.01435	C45E1.4	WBGene00016663	171885
188382_s_at	2.02560	down	-1.01835	C52B9.8*	WBGene00016868	180705
173429_at	2.26820	down	-1.18155	cdk-4	WBGene00000406	181472
175601_at	2.07108	down	-1.05038	ceh-20	WBGene00000443	3565545
173465_at	2.41219	down	-1.27034	chk-1	WBGene00000498	3565921
175786_at	2.34089	down	-1.22706	cki-1	WBGene00000516	174260
190344_at	2.20686	down	-1.14199	clec-196	WBGene00009156	178506
175764_s_at	2.57972	down	-1.36722	clh-3	WBGene00000530	174187
173398_s_at	2.55895	down	-1.35555	cnc-7*	WBGene00010005	180337
172240_x_at	3.20165	down	-1.67882	cnc-7 *	WBGene00010005	180337
175247_s_at	2.17671	down	-1.12215	cTel55X.1	WBGene00007068	181791
188900_at	2.57840	down	-1.36648	ctr-9*	WBGene00007184	176335
175602_at	2.01796	down	-1.01289	cul-1	WBGene00000836	176466
**173185_s_at**	**2.15732**	**down**	**-1.10924**	**D1043.1**	**WBGene00008361**	**174777**
190646_s_at	2.01751	down	-1.01257	ddo-2	WBGene00017565	179130
193932_s_at	2.00236	down	-1.00170	dnj-17	WBGene00001035	176761
182470_at	5.60096	down	-2.48568	F09F7.6	WBGene00017303	184260
174649_s_at	2.01300	down	-1.00935	F13H8.2	WBGene00017435	174038
180425_s_at	3.02041	down	-1.59474	F15E6.3	WBGene00017484	177195
186521_at	2.01465	down	-1.01053	F21C10.10*	WBGene00017659	179290
187613_at	2.11831	down	-1.08292	F22D6.8*	WBGene00043279	4363015
174514_at	2.12010	down	-1.08413	F26F12.3	WBGene00017834	178972
173086_s_at	2.93479	down	-1.55326	F26F12.3	WBGene00017834	178972
174420_at	2.25216	down	-1.17131	F29C6.1	WBGene00009243	172947

174067_at	2.02129	down	-1.01528	F33H1.4	WBGene00009365	174580
173629_at	2.28682	down	-1.19334	F36A2.13	WBGene00009460	172697
187019_at	2.08845	down	-1.06243	F37C12.10	WBGene00018153	185422
187391_at	2.00271	down	-1.00195	F40B5.1	WBGene00018225	185512
173550_at	2.72834	down	-1.44802	F45D3.4*	WBGene00009724	179718
175278_at	2.86457	down	-1.51832	F46A8.7*	WBGene00009750	185823
180098_s_at	2.48319	down	-1.31220	F49E2.5	WBGene00009888	181175
174012_s_at	2.22870	down	-1.15620	F53B2.8	WBGene00009957	178203
174415_at	2.14591	down	-1.10159	F53B3.5	WBGene00018743	180578
182757_at	2.22093	down	-1.15116	F53F4.14*	WBGene00009996	179852
179078_s_at	2.03969	down	-1.02835	F54C9.3	WBGene00010041	174369
178192_at	2.12972	down	-1.09066	F54C9.9	WBGene00010044	174374
182088_s_at	38.09506	down	-5.25153	F57F4.4	WBGene00019017	3564957
179523_at	2.26317	down	-1.17835	F59A2.5*	WBGene00010305	175444
173696_at	2.14082	down	-1.09816	fbf-2	WBGene00001402	174017
172220_x_at	2.10182	down	-1.07164	fipr-23	WBGene00007989	173283
191759_at	3.02902	down	-1.59885	fmo-2*	WBGene00001477	177958
174574_at	2.19332	down	-1.13312	gmps-1	WBGene00010912	174672
173341_at	5.02048	down	-2.32783	haf-6	WBGene00001816	171694
**194214_x_at**	**2.04421**	**down**	**-1.03155**	**hcp-2**	**WBGene00001830**	**179353**
191712_s_at	2.26523	down	-1.17965	icl-1	WBGene00001564	178583
173301_s_at	4.87536	down	-2.28551	icl-1	WBGene00001564	178583
194142_x_at	3.12544	down	-1.64406	icl-1	WBGene00001564	178583
180721_at	5.41142	down	-2.43601	ilys-2	WBGene00016669	183475
180946_at	6.65323	down	-2.73405	ilys-3	WBGene00016670	177033
180815_at	2.02369	down	-1.01698	K01H12.1	WBGene00010484	186854
172998_s_at	2.04023	down	-1.02873	K07A1.6	WBGene00010613	172797
173190_s_at	2.11630	down	-1.08155	K08A2.4*	WBGene00019513	173643
191313_at	2.02656	down	-1.01903	K10C3.5	WBGene00010732	172838
173634_at	2.03807	down	-1.02720	lact-3*	WBGene00010897	174644
187999_at	2.12534	down	-1.08769	lea-1	WBGene00002263	3564838
174552_s_at	3.26460	down	-1.70690	lea-1	WBGene00002263	3564838
173232_s_at	2.25943	down	-1.17596	lmp-2*	WBGene00015471	180467
184694_at	7.69971	down	-2.94480	lys-10	WBGene00003099	184622
**173362_s_at**	**2.44661**	**down**	**-1.29079**	**M03C11.2***	**WBGene00010839**	**176457**
181968_s_at	2.19844	down	-1.13648	mac-1	WBGene00003119	174974
194188_x_at	2.35817	down	-1.23767	mps-2	WBGene00019282	173419
**193857_s_at**	**3.26952**	**down**	**-1.70908**	**msh-5**	**WBGene00003421**	**178268**
172744_at	17.33867	down	-4.11592	mtl-1*	WBGene00003473	179060

178512_at	2.88412	down	-1.52813	nlp-39	WBGene00008295	183774
172268_x_at	2.14778	down	-1.10284	nspc-20*	WBGene00008356	181549
174923_at	2.08022	down	-1.05673	ntl-4*	WBGene00003827	177580
173471_at	2.14734	down	-1.10255	plk-2*	WBGene00004043	171838
188279_at	2.44021	down	-1.28701	polk-1	WBGene00017696	176209
173487_at	2.28395	down	-1.19153	R04D3.2	WBGene00011010	181451
191364_at	2.36960	down	-1.24465	R05H10.5	WBGene00011045	187630
179476_at	2.21504	down	-1.14733	R07E5.1	WBGene00011109	175574
190514_at	6.27039	down	-2.64856	R10E4.11	WBGene00011206	175554
180522_at	2.22923	down	-1.15654	R11G1.2	WBGene00020011	187816
**189050_at**	**2.21158**	**down**	**-1.14508**	**R74.6**	**WBGene00011280**	**187888**
185637_at	2.01391	down	-1.01000	roik-1	WBGene00019698	171883
**190859_s_at**	**4.16545**	**down**	**-2.05847**	**sel-5**	**WBGene00004762**	**175599**
173195_s_at	2.41617	down	-1.27272	smu-2	WBGene00004896	173693
188388_at	2.63219	down	-1.39626	spg-7	WBGene00004978	171915
178149_s_at	2.11180	down	-1.07847	spp-1	WBGene00004986	176452
174411_s_at	2.04177	down	-1.02982	T01H3.2	WBGene00011348	174271
174180_at	2.14385	down	-1.10020	T02E9.5	WBGene00011383	179568
177899_at	3.30412	down	-1.72426	T03D8.7	WBGene00011394	180363
179805_s_at	2.02226	down	-1.01597	T04G9.7	WBGene00020218	180424
186427_at	2.13525	down	-1.09441	T05H4.11*	WBGene00020274	179024
174789_at	2.04914	down	-1.03502	T09B4.5	WBGene00020379	172300
172193_at	9.47063	down	-3.24346	T12D8.5	WBGene00011733	176797
179094_s_at	2.10954	down	-1.07693	T16G1.4*	WBGene00011798	179776
173020_s_at	3.44097	down	-1.78281	T19A5.1 *	WBGene00020553	179275
173496_s_at	3.60722	down	-1.85089	T23G7.3	WBGene00011966	174456
193610_s_at	3.24662	down	-1.69894	taf-1	WBGene00006382	173257
171855_x_at	2.30497	down	-1.20475	tcc-1	WBGene00013340	180167
181127_s_at	2.86219	down	-1.51712	thoc-2	WBGene00015813	175900
193643_s_at	2.49799	down	-1.32077	trxr-1*	WBGene00015553	177466
171783_x_at	2.56619	down	-1.35963	ubc-1	WBGene00006701	177170
175566_s_at	2.27868	down	-1.18820	ugt-50	WBGene00011564	181413
174606_at	2.12963	down	-1.09060	utx-1	WBGene00017046	181110
189587_s_at	2.72560	down	-1.44657	W03F9.4	WBGene00021002	178543
174109_at	6.97238	down	-2.80165	Y102A5C.6	WBGene00014955	
187234_s_at	4.36119	down	-2.12472	Y110A2AL.9	WBGene00022445	173632
171895_s_at	2.24218	down	-1.16490	Y17G7B.17	WBGene00012468	174849
174752_at	2.25226	down	-1.17137	Y39A3CL.1	WBGene00021440	175329
172668_x_at	2.30313	down	-1.20360	Y39A3CL.3*	WBGene00021441	189725

173609_at	2.94121	down	-1.55641	Y39B6A.1	WBGene00012664	180264
176082_s_at	2.16887	down	-1.11694	Y41D4B.6	WBGene00021513	176974
181789_at	2.35508	down	-1.23578	Y47H9C.8	WBGene00012951	173068
173297_at	2.49411	down	-1.31852	Y48G8AL.13*	WBGene00021691	171699
173257_s_at	2.64024	down	-1.40067	Y53F4B.45	WBGene00044728	
176209_at	2.02557	down	-1.01833	Y58A7A.5*	WBGene00021979	190383
176183_at	4.64676	down	-2.21623	Y69A2AR.25	WBGene00022096	190546
185811_at	2.05850	down	-1.04159	Y71H2B.4	WBGene00022194	175381
173410_s_at	2.00814	down	-1.00586	Y87G2A.1	WBGene00013593	173221
180427_at	3.00260	down	-1.58621	ZC395.5*	WBGene00022597	191152
173558_at	2.81234	down	-1.49177	ZC443.3	WBGene00013898	179758
174073_at	4.64250	down	-2.21490			

Genes Upregulated in *hlh-25* Mutants

Probe Set ID	FCAbsolute	regulation	log2 FC	Gene Symbol	WormBase Gene ID	Entrez Gene
177193_s_at	4.99339	up	2.32002	abu-1*	WBGene00000024	181800
179983_at	4.79301	up	2.26093	abu-10*	WBGene00000033	185248
173482_s_at	2.96507	up	1.56807	abu-11*	WBGene00000034	173404
172323_x_at	6.39367	up	2.67664	abu-6*	WBGene00000029	178903
172321_x_at	4.84522	up	2.27656	abu-7	WBGene00000030	178904
172587_x_at	4.95913	up	2.31009	abu-8 *	WBGene00000031	178904
194211_x_at	4.30954	up	2.10753	abu-8 *	WBGene00000031	178904
185654_s_at	2.03238	up	1.02317	abu-9*	WBGene00000032	187749
193635_s_at	2.31687	up	1.21218	acl-2	WBGene00011543	179398
174833_at	2.46321	up	1.30054	acs-1	WBGene00018488	179043
191641_s_at	2.29608	up	1.19917	acs-19	WBGene00007969	175500
AFFX-r2-13514-3_s_at	5.72112	up	2.51630	act-1	WBGene00000064	179533
AFFX-r2-13514-5_x_at	8.63106	up	3.10954	act-1	WBGene00000065	179533
AFFX-Ce_Actin_5_f_at	11.03174	up	3.46359	act-1*	WBGene00000063	
AFFX-Ce_Actin_M_s_at	5.75383	up	2.52452	act-1*	WBGene00000063	
190043_s_at	5.34771	up	2.41892	act-1 *	WBGene00000063	179533
172816_x_at	2.74062	up	1.45450	act-2*	WBGene00000063	179533
189551_s_at	2.20945	up	1.14369	act-5	WBGene00000067	176793
**191329_s_at**	**2.12977**	**up**	**1.09070**	**air-1***	**WBGene00000098**	**179202**
189114_at	2.16346	up	1.11334	ampd-1	WBGene00016415	173891
186867_s_at	2.04306	up	1.03073	anat-1	WBGene00015938	177439
174791_s_at	2.06205	up	1.04408	anc-1	WBGene00000140	172034
173676_s_at	2.34910	up	1.23211	apy-1	WBGene00017244	181019

189807_at	3.36129	up	1.74902	aqp-7	WBGene00000175	180589
192156_at	2.78453	up	1.47743	arf-3*	WBGene00000183	177595
173972_s_at	2.53520	up	1.34210	art-1	WBGene00000198	174140
173989_s_at	2.32190	up	1.21531	asp-6*	WBGene00000219	179209
189086_s_at	3.07307	up	1.61968	atn-1	WBGene00018488	179709
188137_s_at	2.20591	up	1.14138	atp-3	WBGene00000230	172195
193256_at	2.07521	up	1.05326	B0393.3	WBGene00007168	175630
187506_s_at	2.49310	up	1.31794	B0416.5	WBGene00015181	181158
187297_s_at	2.15766	up	1.10947	B0495.7	WBGene00015206	174245
175994_s_at	3.12701	up	1.64478	bath-42	WBGene00016803	176152
174152_s_at	2.25633	up	1.17398	bli-1	WBGene00000251	174653
188391_at	2.53678	up	1.34300	bli-2	WBGene00000252	191611
191062_s_at	2.00611	up	1.00440	bli-4	WBGene00000254	172333
184599_at	2.39537	up	1.26025	C01B10.6	WBGene00015280	177432
172353_x_at	2.52659	up	1.33719	C02E7.6	WBGene00015339	178885
172325_x_at	2.49361	up	1.31824	C02E7.7	WBGene00015340	178884
183211_s_at	4.27528	up	2.09602	C04F12.7	WBGene00007301	172819
177577_s_at	2.68114	up	1.42284	C04G2.8	WBGene00007307	177877
**187292_s_at**	**2.16427**	**up**	**1.11388**	**C05C10.5**	**WBGene00007332**	**174551**
177934_s_at	2.06550	up	1.04649	C06A1.6	WBGene00007356	174625
179164_at	2.01153	up	1.00830	C06G8.1	WBGene00007384	177972
188162_at	2.66530	up	1.41430	C09H5.2	WBGene00019875	179189
188163_s_at	2.40738	up	1.26747	C10G11.8	WBGene00015688	179189
186105_s_at	3.72186	up	1.89602	C10G11.9	WBGene00015689	172293
190329_at	2.83003	up	1.50081	C11E4.1	WBGene00007516	181178
184411_at	2.11154	up	1.07829	C12D12.1	WBGene00015713	180642
191943_at	2.01161	up	1.00835	C14C10.1	WBGene00007584	182608
183526_at	2.42174	up	1.27604	C14C6.5*	WBGene00015759	178574
184320_at	4.07984	up	2.02851	C14F11.4	WBGene00015780	182619
181473_s_at	2.87685	up	1.52449	C15C6.2*	WBGene00007601	173099
190619_at	2.19918	up	1.13697	C15C8.3	WBGene00007605	182635
**187346_at**	**2.50465**	**up**	**1.32461**	**C16C10.3***	**WBGene00007624**	**175535**
187412_at	2.32412	up	1.21668	C18H9.6*	WBGene00016004	182809
189758_s_at	2.17872	up	1.12348	C25A1.4*	WBGene00007706	172881
186257_s_at	3.01128	up	1.59038	C29H12.2	WBGene00016235	174022
**175939_s_at**	**2.24848**	**up**	**1.16895**	**C30A5.3***	**WBGene00016238**	**176158**
180909_at	2.28174	up	1.19013	C32H11.4	WBGene00007867	178244
180997_at	3.02588	up	1.59735	C34D4.3	WBGene00016399	177488
181291_at	2.53047	up	1.33941	C34D4.4	WBGene00016400	183205

183115_at	2.96271	up	1.56692	C34H4.2	WBGene00016425	183220
179035_s_at	2.47370	up	1.30667	C35A5.8	WBGene00007952	179467
181840_s_at	2.00966	up	1.00695	C35C5.3*	WBGene00007955	181343
182691_s_at	2.47417	up	1.30694	C36A4.5	WBGene00007966	172918
180231_at	2.49613	up	1.31969	C37C3.9*	WBGene00016501	179164
192234_s_at	2.91803	up	1.54500	C38C10.2	WBGene00008000	176317
189054_at	2.48832	up	1.31517	C39H7.1	WBGene00016541	177341
180540_at	3.27268	up	1.71047	C42D4.3	WBGene00016596	177492
183193_at	2.15666	up	1.10880	C46G7.2	WBGene00016722	177367
183956_at	2.00333	up	1.00240	C48E7.1	WBGene00016749	172322
179858_at	2.35668	up	1.23675	C49C8.5	WBGene00016769	177668
178517_at	2.39023	up	1.25715	C49F8.3	WBGene00008215	181459
192995_at	2.13310	up	1.09295	C50F7.4*	WBGene00016844	177555
179176_at	2.65226	up	1.40722	C52G5.2*	WBGene00008260	181420
180628_at	3.39175	up	1.76203	C53B7.3*	WBGene00016894	180957
180064_at	2.15915	up	1.11046	C54D2.1*	WBGene00016915	183776
180685_at	2.07157	up	1.05073	C54E4.2*	WBGene00016918	177010
190667_s_at	2.85074	up	1.51134	calu-1	WBGene00019760	180769
193984_s_at	2.03876	up	1.02769	cdc-48.1	WBGene00007352	174624
**188316_s_at**	**2.12480**	**up**	**1.08733**	**cdl-1**	**WBGene00000411**	**174659**
**187802_at**	**2.51161**	**up**	**1.32861**	**cgh-1**	**WBGene00000479**	**176061**
**193886_at**	**3.29850**	**up**	**1.72181**	**cks-1***	**WBGene00001051**	**177658**
184002_at	2.85086	up	1.51140	clc-1	WBGene00000522	181293
186278_at	2.48240	up	1.31173	clec-150	WBGene00019914	175261
184035_at	2.10788	up	1.07579	clec-265	WBGene00019738	180590
189915_s_at	2.14301	up	1.09964	clec-41	WBGene00007153	179797
192509_at	2.16135	up	1.11193	clec-60	WBGene00014046	191384
176743_s_at	2.02588	up	1.01855	clec-67	WBGene00018971	177151
188109_s_at	2.64431	up	1.40289	clec-87	WBGene00007709	172885
189797_s_at	2.82274	up	1.49709	col-104	WBGene00000678	176947
173235_at	3.03217	up	1.60035	col-109*	WBGene00000683	176988
172879_x_at	2.07574	up	1.05363	col-12	WBGene00000601	179452
172701_x_at	2.62046	up	1.38982	col-125	WBGene00000699	178029
172878_x_at	2.24083	up	1.16403	col-13	WBGene00000601	179452
188364_s_at	3.33723	up	1.73865	col-130	WBGene00000704	178193
183666_at	3.33237	up	1.73655	col-138	WBGene00000711	183722
188500_at	2.71134	up	1.43900	col-142	WBGene00000715	188514
172475_x_at	3.89070	up	1.96003	col-145	WBGene00000718	179297
171735_x_at	2.07681	up	1.05437	col-147	WBGene00000720	179351

188350_at	2.12146	up	1.08506	col-149	WBGene00000722	179431
172700_x_at	2.21459	up	1.14704	col-157	WBGene00000730	188426
172746_x_at	3.26193	up	1.70572	col-166	WBGene00000742	180902
172735_x_at	2.97588	up	1.57332	col-167	WBGene00000740	180907
189482_s_at	2.40100	up	1.26364	col-175	WBGene00000748	181154
188475_at	2.79251	up	1.48156	col-180	WBGene00000753	181356
189968_s_at	2.75721	up	1.46321	col-38	WBGene00000615	174370
190584_at	2.53568	up	1.34237	col-39	WBGene00000616	174651
190134_s_at	2.19661	up	1.13528	col-41*****	WBGene00000618	181610
188747_at	4.18272	up	2.06444	col-48	WBGene00000625	171873
189762_at	2.50837	up	1.32675	col-49	WBGene00000626	187239
173767_s_at	2.73004	up	1.44892	col-62	WBGene00000596	172494
189099_at	4.04222	up	2.01515	col-77	WBGene00000653	174336
190093_at	2.48279	up	1.31196	col-88	WBGene00000663	175170
177032_at	3.38155	up	1.75769	col-91	WBGene00000666	184269
188473_s_at	3.04046	up	1.60429	col-97	WBGene00000672	176721
188113_s_at	2.16606	up	1.11508	cox-15	WBGene00011526	174712
**187525_s_at**	**2.46684**	**up**	**1.30267**	**cpg-2**	**WBGene00015102**	**175991**
192114_s_at	2.36435	up	1.24145	cth-2	WBGene00022856	174155
178608_s_at	2.09800	up	1.06901	cup-2*****	WBGene00000843	172915
172706_x_at	3.45465	up	1.78854	cuticlin (F53F1.4)	WBGene00009982	179822
172741_x_at	3.75105	up	1.90729	Cuticlin (F41F3.3)	WBGene00018297	178856
**172738_x_at**	**2.95630**	**up**	**1.56379**	**cyb-2.1**	**WBGene00000866**	**177994**
**172615_x_at**	**2.75252**	**up**	**1.46075**	**cyb-2.2**	**WBGene00000867**	**171993**
**194213_x_at**	**3.38700**	**up**	**1.76001**	**cyb-2.2**	**WBGene00000867**	**171993**
**188916_s_at**	**2.80402**	**up**	**1.48750**	**cyb-3**	**WBGene00000868**	**180040**
178786_s_at	2.57252	up	1.36318	D1081.7	WBGene00008385	172638
177920_at	3.02031	up	1.59470	D1086.3	WBGene00008390	179907
187176_s_at	3.21072	up	1.68289	D2096.6	WBGene00017073	177633
188215_at	2.97808	up	1.57438	daf-18*	WBGene00000913	176869
**188460_s_at**	**2.19884**	**up**	**1.13674**	**daz-1***	**WBGene00000935**	**173931**
177978_at	2.24693	up	1.16796	dct-18	WBGene00010266	174934
188558_s_at	3.51117	up	1.81195	dim-1	WBGene00001000	181062
173606_s_at	2.11260	up	1.07902	dlc-1	WBGene00001005	175922
189606_s_at	2.08120	up	1.05742	dnj-19	WBGene00001037	3565862
177463_at	2.02057	up	1.01476	dod-19	WBGene00022644	178564
172218_x_at	2.80608	up	1.48856	dod-6*	WBGene00011869	176433
187205_s_at	2.04749	up	1.03386	dpff-1*	WBGene00016200	175832
190360_s_at	3.00888	up	1.58923	dpy-5	WBGene00001067	172197

180747_s_at	2.24597	up	1.16734	dsc-4	WBGene00001099	176852
177729_at	3.74121	up	1.90351	E01G4.6	WBGene00008448	174990
193298_s_at	2.73133	up	1.44961	eat-6	WBGene00001137	179796
193064_at	2.03858	up	1.02756	eef-1B.2	WBGene00012768	178419
190244_s_at	2.22306	up	1.15254	egl-21	WBGene00001189	177940
191276_s_at	3.65283	up	1.86901	elo-5	WBGene00001243	177320
173725_s_at	2.61705	up	1.38794	elo-6	WBGene00001244	177321
192676_s_at	2.17146	up	1.11867	emb-5	WBGene00001259	175621
190353_s_at	3.43813	up	1.78163	emb-9	WBGene00001263	176314
AFFX-r2-3026-5_at	2.41198	up	1.27022	enol-1	WBGene00011884	174423
177953_s_at	3.30550	up	1.72487	ent-2*	WBGene00010701	181648
182964_s_at	2.01310	up	1.00942	ergo-1	WBGene00019971	178602
189677_s_at	2.80319	up	1.48707	erm-1	WBGene00001333	172174
192910_s_at	2.72480	up	1.44615	F01G4.6	WBGene00008505	178020
188758_at	2.84345	up	1.50764	F07H5.8	WBGene00008559	174407
183194_s_at	2.21621	up	1.14809	F08F3.6	WBGene00017263	178925
183159_at	2.02530	up	1.01814	F09F9.2	WBGene00017307	184263
191454_at	2.11365	up	1.07973	F10G8.7	WBGene00008665	172867
**181081_at**	**2.66038**	**up**	**1.41163**	**F11A10.5***	**WBGene00008686**	**178152**
179439_s_at	2.76979	up	1.46978	F11E6.3	WBGene00008707	178533
**188584_s_at**	**2.41958**	**up**	**1.27476**	**F14B4.2**	**WBGene00008780**	**174333**
176786_at	2.21912	up	1.14999	F14H12.3	WBGene00017471	180709
178625_s_at	3.43432	up	1.78003	F14H3.6	WBGene00008825	180077
186492_s_at	3.02105	up	1.59505	F15E11.1	WBGene00017490	178710
186519_at	2.92835	up	1.55009	F15E11.12	WBGene00017498	178712
177131_s_at	2.15912	up	1.11044	F15E11.13	WBGene00017499	178709
181257_at	2.57681	up	1.36559	F17C11.2	WBGene00008914	184614
**175074_s_at**	**2.35833**	**up**	**1.23777**	**F18A1.7**	**WBGene00017548**	**174237**
178164_at	2.10092	up	1.07102	F21H7.5	WBGene00009031	180090
187770_s_at	2.35600	up	1.23634	F25B5.3	WBGene00017775	175843
194218_x_at	2.20529	up	1.14097	F25D7.4	WBGene00009113	172918
172775_x_at	2.16286	up	1.11294	F25D7.4	WBGene00009113	172918
181410_s_at	2.82273	up	1.49709	F26G1.5	WBGene00017841	173823
183734_at	2.32308	up	1.21604	F27C1.1	WBGene00017851	172198
181937_s_at	2.08541	up	1.06033	F27C1.2	WBGene00017852	172196
187429_s_at	2.12729	up	1.08902	F31E3.4*	WBGene00017951	175974
189761_s_at	2.29851	up	1.20070	F32B5.1*	WBGene00017975	173236
192078_at	2.27793	up	1.18772	F32B6.2	WBGene00009319	177841
185764_s_at	2.10595	up	1.07447	F33D4.6*	WBGene00017998	177552

176681_at	2.30441	up	1.20440	F35B3.4	WBGene00018031	185255
185153_s_at	2.08501	up	1.06005	F35E12.10	WBGene00009434	179868
179695_at	2.84233	up	1.50707	F35E12.5	WBGene00009429	185307
185480_s_at	2.38707	up	1.25524	F36H12.8	WBGene00018122	177276
187476_at	2.02052	up	1.01473	F37A4.1*	WBGene00018131	175948
184083_s_at	2.14928	up	1.10386	F37B12.3*	WBGene00009505	174439
181941_s_at	2.23337	up	1.15922	F37C4.5*	WBGene00018145	177146
173727_s_at	2.88791	up	1.53002	F40F4.6	WBGene00018237	180613
185537_at	3.39274	up	1.76245	F41E6.11	WBGene00018292	179245
186757_s_at	2.71824	up	1.44267	F42A8.1	WBGene00009626	174481
190977_s_at	2.28372	up	1.19138	F42D1.2*	WBGene00009628	181574
190958_s_at	2.36332	up	1.24081	F44E5.4*	WBGene00009691	174805
182163_s_at	2.00320	up	1.00231	F44E7.5	WBGene00018427	178967
186801_s_at	2.36351	up	1.24093	F45D11.14	WBGene00018459	173480
184276_at	2.03535	up	1.02528	F46F11.6	WBGene00018509	172218
179187_s_at	2.69422	up	1.42987	F46F2.3	WBGene00009787	181619
189706_s_at	2.18530	up	1.12783	F46H5.3	WBGene00018519	180986
191301_s_at	2.06672	up	1.04735	F47B10.2	WBGene00009813	181279
190419_s_at	2.05987	up	1.04255	F47G9.1*	WBGene00009829	179567
183020_at	2.03937	up	1.02812	F48G7.8	WBGene00018619	185996
186468_s_at	2.50372	up	1.32407	F49C12.12	WBGene00009881	177755
181311_s_at	2.24230	up	1.16498	F49C12.9*	WBGene00009878	177752
178587_s_at	2.29119	up	1.19610	F52A8.1	WBGene00009915	172482
182970_at	2.89645	up	1.53429	F55G11.4	WBGene00010124	178248
180707_at	2.34325	up	1.22851	F55H12.4	WBGene00010135	186344
182717_at	2.27705	up	1.18717	F56B3.6	WBGene00018930	176906
188635_at	2.81536	up	1.49332	F58E6.13	WBGene00077697	6418750
177974_s_at	2.23964	up	1.16326	F58G1.1	WBGene00010263	174932
172190_x_at	2.65311	up	1.40768	F59F4.2*	WBGene00010337	181669
183396_s_at	2.99084	up	1.58055	farl-11	WBGene00017349	174165
192186_at	2.05141	up	1.03661	fat-2*	WBGene00001394	178293
179338_at	2.20772	up	1.14256	frm-10	WBGene00001496	179828
193200_s_at	2.37643	up	1.24880	gale-1	WBGene00008132	173171
190626_s_at	5.58029	up	2.48034	gdh-1*	WBGene00014095	178130
188046_s_at	16.28070	up	4.02509	gfi-1	WBGene00001581	179022
190303_s_at	2.06318	up	1.04487	gly-8	WBGene00001633	176595
AFFXCe_Gapdh_M_s_at	3.14864	up	1.65473	gpd-3	WBGene00001685	
185646_at	2.73173	up	1.44982	grd-14	WBGene00001703	187934
181972_at	2.22868	up	1.15619	grd-3	WBGene00001692	177109

180979_at	2.13624	up	1.09508	grd-5	WBGene00001694	179244
188028_at	2.71935	up	1.44326	grd-6	WBGene00001695	179305
186927_s_at	2.31360	up	1.21014	grl-16*	WBGene00001725	171649
182143_s_at	2.34199	up	1.22774	grl-4	WBGene00001713	3564844
173004_s_at	2.64340	up	1.40239	grl-7	WBGene00001716	179569
187085_s_at	2.58184	up	1.36840	gst-10*	WBGene00001758	178725
190852_at	2.12030	up	1.08427	gst-13	WBGene00001761	188915
**181288_at**	**2.24946**	**up**	**1.16958**	**H02I12.5***	**WBGene00010353**	**178064**
173083_s_at	2.45459	up	1.29548	H10E21.4	WBGene00019184	175168
184288_at	2.05102	up	1.03634	H14A12.3*	WBGene00019196	176058
185593_at	2.54010	up	1.34488	H23N18.5	WBGene00019236	186770
179787_at	2.44103	up	1.28749	H36L18.2	WBGene00010424	181411
174393_at	2.59412	up	1.37524	H40L08.2	WBGene00014793	
173124_s_at	2.06670	up	1.04733	H42K12.3	WBGene00019272	
AFFX-r2-3487-5_s_at	2.07932	up	1.05611	hel-1*	WBGene00001840	180474
**188740_at**	**2.01638**	**up**	**1.01177**	**hil-5**	**WBGene00001856**	**172751**
**183760_s_at**	**2.43204**	**up**	**1.28216**	**him-1**	**WBGene00001860**	**172242**
**188001_s_at**	**2.15270**	**up**	**1.10614**	**him-17**	**WBGene00001874**	**172116**
190225_s_at	2.26938	up	1.18230	his-24	WBGene00001898	179800
172891_x_at	2.14184	up	1.09885	his-48	WBGene00001922	181545
172758_x_at	2.32038	up	1.21436	his-48	WBGene00001922	178049
172702_x_at	2.08274	up	1.05848	his-62	WBGene00001936	178049
184409_s_at	2.56318	up	1.35793	hmg-1.1*	WBGene00001971	186326
176044_at	3.38532	up	1.75929	hp0-6	WBGene00021518	175081
173626_s_at	3.60333	up	1.84933	hpo-32*	WBGene00009259	181340
188444_at	2.30207	up	1.20293	hsp-16.41	WBGene00002018	176965
172901_x_at	2.22904	up	1.15642	hsp-16.48	WBGene00002019	178660
190900_s_at	2.04685	up	1.03341	hsp-25	WBGene00002023	179287
**189914_s_at**	**2.07102**	**up**	**1.05034**	**htz-1**	**WBGene00019947**	**180872**
177684_s_at	2.49077	up	1.31659	icl-1	WBGene00001564	177212
190796_s_at	2.80481	up	1.48790	idh-1*	WBGene00010317	177775
193131_at	2.05506	up	1.03918	ifa-1	WBGene00002050	177730
192118_s_at	2.02219	up	1.01592	ifb-2	WBGene00002054	181316
189324_s_at	2.00638	up	1.00460	ile-1	WBGene00002070	173973
**192178_s_at**	**2.69565**	**up**	**1.43063**	**ima-1***	**WBGene00002072**	**172799**
**192711_at**	**2.16085**	**up**	**1.11160**	**ima-2**	**WBGene00002073**	**179555**
193731_s_at	3.36484	up	1.75054	immt-1	WBGene00020511	172329
173547_s_at	2.07086	up	1.05023	imp-1	WBGene00007979	180566
189792_s_at	2.15281	up	1.10622	imp-2*	WBGene00011481	172684

192422_s_at	2.05060	up	1.03605	ina-1	WBGene00002081	178013
188535_s_at	2.28230	up	1.19049	inf-1	WBGene00002083	176296
192240_s_at	5.17754	up	2.37227	inf-1	WBGene00018997	175966
171811_s_at	2.17749	up	1.12267	K01D12.7*	WBGene00010466	175966
187787_at	2.11033	up	1.07747	k02D10.1	WBGene00019301	176232
193627_at	3.17719	up	1.66775	K02F2.2	WBGene00019322	179698
179892_at	2.84474	up	1.50830	K06A5.2	WBGene00019430	172408
173922_s_at	2.11228	up	1.07880	K07H8.2	WBGene00019504	259316
178927_at	2.78953	up	1.48002	K08D8.5*	WBGene00010659	177623
177700_at	4.66185	up	2.22090	K08D8.6	WBGene00010660	178243
177701_s_at	2.23729	up	1.16175	K08D8.6	WBGene00010660	178241
194129_s_at	2.66204	up	1.41253	K08E3.5	WBGene00010665	178241
189319_at	2.46684	up	1.30267	K08F4.1	WBGene00010676	176814
187580_s_at	2.17728	up	1.12253	K09G1.1	WBGene00010727	177879
190815_s_at	2.71363	up	1.44023	K10C2.1	WBGene00019617	179346
189595_s_at	2.64570	up	1.40365	K10C2.3	WBGene00019619	180915
192003_s_at	2.21892	up	1.14986	K11G12.5	WBGene00019656	180917
**186482_s_at**	**2.15586**	**up**	**1.10826**	**kca-1***	**WBGene00015698**	**180940**
**187874_at**	**2.12927**	**up**	**1.09036**	**kin-3**	**WBGene00002191**	**172090**
**188638_s_at**	**2.54340**	**up**	**1.34676**	**klp-15**	**WBGene00002225**	**172978**
193446_s_at	3.76413	up	1.91232	klp-16	WBGene00002226	172213
194001_s_at	2.75596	up	1.46256	lec-1*	WBGene00002264	172784
192341_s_at	2.41289	up	1.27076	let-75*	WBGene00002348	174964
**183157_s_at**	**3.00312**	**up**	**1.58646**	**lin-37**	**WBGene00003022**	**172471**
**177366_s_at**	**2.12442**	**up**	**1.08707**	**lin-54**	**WBGene00003037**	**175986**
176605_s_at	2.22468	up	1.15360	lir-1	WBGene00003044	178280
193082_s_at	2.37902	up	1.25037	lmn-1	WBGene00003052	174240
187660_s_at	2.13794	up	1.09622	lmp-1	WBGene00003053	172687
188368_at	2.23830	up	1.16241	lon-3*	WBGene00003057	180912
AFFX-r2-18565-5_x_at	4.44784	up	2.15310	mai-1*	WBGene00003124	179673
AFFX-Ce_Gapdh_5_s_at	4.60056	up	2.20181	mai-1	WBGene00003124	174350
177535_s_at	2.09050	up	1.06385	MBOA-6	WBGene00020115	176016
**193664_s_at**	**2.07894**	**up**	**1.05585**	**mcm-3**	**WBGene00003155**	**3565232**
**173443_s_at**	**2.46743**	**up**	**1.30301**	**mei-2**	**WBGene00003184**	**180010**
**193943_at**	**2.06826**	**up**	**1.04841**	**mex-5**	**WBGene00003230**	**172374**
**192002_at**	**2.17034**	**up**	**1.11792**	**mex-6**	**WBGene00003231**	**178296**
190057_s_at	3.28763	up	1.71705	mig-6	WBGene00003242	179224
185322_s_at	2.39461	up	1.25979	mlp-1	WBGene00003375	174504
187408_s_at	2.15781	up	1.10957	mogs-1*	WBGene00008775	177998

**179898_s_at**	**2.06763**	**up**	**1.04798**	**mrg-1**	**WBGene00003406**	**175847**
193046_at	3.12463	up	1.64369	mrp-4	WBGene00003410	176702
183958_s_at	2.36102	up	1.23941	MTCH-1	WBGene00018395	174207
173556_s_at	2.21505	up	1.14734	mthf-1	WBGene00015512	174254
173285_s_at	2.24516	up	1.16682	ncx-1	WBGene00003566	181202
187929_s_at	2.46059	up	1.29900	ndg-4	WBGene00003577	180318
191753_at	2.68600	up	1.42546	NEP-17	WBGene00010070	174996
188026_at	2.13004	up	1.09088	nex-1	WBGene00003588	175582
184985_s_at	2.48704	up	1.31443	nlp-40*	WBGene00022276	175714
**184943_s_at**	**2.99886**	**up**	**1.58441**	**nos-2***	**WBGene00003784**	**171591**
**188236_s_at**	**2.23052**	**up**	**1.15738**	**nos-2***	**WBGene00003784**	**174158**
191157_s_at	2.70489	up	1.43557	npp-12	WBGene00003798	174158
179133_at	2.04446	up	1.03172	NRA-1	WBGene00011488	176788
185454_s_at	2.78433	up	1.47733	nspb-1	WBGene00018162	172348
172469_x_at	3.13336	up	1.64771	nspb-10	WBGene00007246	185443
172474_x_at	2.00632	up	1.00455	nspb-11*	WBGene00009398	182088
172344_x_at	2.33607	up	1.22408	nspb-12	WBGene00017305	185265
172595_x_at	2.61925	up	1.38915	nspb-2	WBGene00018162	184261
172417_x_at	2.94094	up	1.55628	nspb-3	WBGene00018162	185443
185645_s_at	2.04218	up	1.03011	nstp-2*	WBGene00017480	177424
187818_s_at	2.20317	up	1.13958	nurf-1	WBGene00009180	185443
**189867_s_at**	**2.38188**	**up**	**1.25210**	**oma-1***	**WBGene00003864**	**175098**
**189988_s_at**	**2.96016**	**up**	**1.56567**	**oma-2**	**WBGene00003865**	**177703**
193068_at	3.20311	up	1.67947	opt-2	WBGene00003877	179183
191389_s_at	2.11166	up	1.07838	ost-1	WBGene00003893	180919
194118_s_at	2.07389	up	1.05234	pab-1*	WBGene00003902	176931
173882_s_at	2.08967	up	1.06328	pab-2	VWBGene00003903	172920
**177834_s_at**	**3.54755**	**up**	**1.82682**	**patr-1***	**WBGene00009661**	**181473**
191298_s_at	2.45518	up	1.29583	pcp-2	WBGene00003957	174808
192032_s_at	2.00937	up	1.00675	pdi-6	WBGene00015168	177741
176873_s_at	3.10960	up	1.63673	perm-3*	WBGene00022776	180974
192285_at	2.07257	up	1.05142	pfd-3	WBGene00006889	174191
182024_at	2.17601	up	1.12168	pfn-2*	WBGene00003990	173141
190248_s_at	2.79126	up	1.48092	pgp-14	WBGene00004008	180808
180962_s_at	2.21761	up	1.14901	pgrn-1	WBGene00011936	178777
194228_x_at	2.05370	up	1.03823	pgrn-1	WBGene00011936	173054
182713_s_at	2.05814	up	1.04134	phat-3	WBGene00016781	178809
182573_at	2.92596	up	1.54891	phat-4	WBGene00020237	181416
181762_s_at	2.80537	up	1.48819	pisy-1	WBGene00012897	178812

**191208_s_at**	**3.00971**	**up**	**1.58963**	**plk-3**	**WBGene00004044**	**174902**
188111_at	2.59410	up	1.37524	plp-1	WBGene00004046	266900
**192079_s_at**	**3.77060**	**up**	**1.91479**	**pos-1**	**WBGene00004078**	**177540**
191504_at	2.06631	up	1.04706	pps-1	WBGene00004091	179162
187280_s_at	2.28044	up	1.18931	ppw-2	WBGene00004094	177893
185070_s_at	2.18892	up	1.13022	pqn-22	WBGene00004112	172152
180505_at	2.86081	up	1.51642	pqn-32	WBGene00004120	177369
183975_s_at	2.02240	up	1.01607	pqn-46	WBGene00004133	174955
172360_x_at	7.77992	up	2.95976	pqn-5	WBGene00004099	175970
183344_at	5.70779	up	2.51293	pqn-54	WBGene00004139	182136
179437_s_at	3.36556	up	1.75085	pqn-74*	WBGene00004156	187728
172443_x_at	2.49704	up	1.32022	pqn-91	WBGene00004170	178294
183577_at	3.41566	up	1.77217	pqn-95*	WBGene00004174	190671
174071_at	3.95570	up	1.98393	pqn-95*	WBGene00004174	174464
**177402_s_at**	**2.61558**	**up**	**1.38713**	**prg-1**	**WBGene00004178**	**174464**
179616_at	2.04505	up	1.03213	prx-14	WBGene00004199	172515
175239_at	3.66044	up	1.87202	pud-3*	WBGene00017501	178711
**194076_s_at**	**3.01185**	**up**	**1.59065**	**puf-3***	**WBGene00004239**	**178026**
**188144_s_at**	**2.28995**	**up**	**1.19531**	**puf-8**	**WBGene00004244**	**178320**
193700_s_at	2.14681	up	1.10220	pxf-1	WBGene00004254	174197
189418_s_at	2.08112	up	1.05736	PYK-1	WBGene00009126	172744
193523_at	2.04895	up	1.03488	qdpr-1*	WBGene00011398	177894
189080_at	2.12397	up	1.08676	R02D3.1	WBGene00019819	176842
183661_at	2.36587	up	1.24237	R02F2.1	WBGene00019831	176762
189696_s_at	2.13310	up	1.09295	R05G6.7	WBGene00019900	175757
191828_at	2.36316	up	1.24071	R08E3.1	WBGene00019957	177524
182747_at	2.49547	up	1.31931	R08E5.3	WBGene00019963	180758
192726_at	2.04977	up	1.03546	R11H6.2	WBGene00011250	178795
190616_at	2.35689	up	1.23688	R12C12.1*	WBGene00020022	179964
172547_x_at	3.26982	up	1.70921	R12E2.14	WBGene00020039	174012
171795_x_at	2.70173	up	1.43388	R12E2.14	WBGene00020039	172001
172497_x_at	4.60786	up	2.20410	R12E2.15	WBGene00020040	172001
172346_x_at	5.24090	up	2.38982	R12E2.7	WBGene00020033	172000
192383_s_at	3.42270	up	1.77513	R151.2	WBGene00020107	172002
189752_s_at	2.06616	up	1.04695	R53.2	WBGene00011272	175339
189793_at	2.80140	up	1.48615	rab-5*	WBGene00004268	174553
190167_s_at	2.44436	up	1.28946	rab-6.1	WBGene00004269	172755
191142_s_at	2.36470	up	1.24166	rad-23	WBGene00013924	176275
188311_s_at	6.73507	up	2.75169	ram-2	WBGene00004300	174785

189729_s_at	2.81688	up	1.49410	ran-1*	WBGene00004302	174687
192220_at	2.27523	up	1.18601	rer-1	WBGene00009783	176503
190792_s_at	2.96265	up	1.56689	ret-1	WBGene00004336	174410
193165_at	2.68164	up	1.42312	rhr-1	WBGene00004358	179981
189552_at	2.19476	up	1.13406	ribo-1*	WBGene00020683	177456
179134_s_at	2.04319	up	1.03082	rmd-3	WBGene00007190	178927
190655_at	2.10888	up	1.07648	rnp-1	WBGene00004384	174732
**181020_at**	**2.08841**	**up**	**1.06240**	**rod-1**	**WBGene00018900**	**179672**
188337_at	2.35086	up	1.23319	rol-1	WBGene00004394	177520
192968_s_at	2.29877	up	1.20086	rol-8	WBGene00004398	174857
**190813_at**	**2.45808**	**up**	**1.29753**	**rpa-1**	**WBGene00017546**	**174226**
193674_s_at	2.07018	up	1.04976	rsp-2	WBGene00004699	174238
192547_s_at	2.02236	up	1.01604	sams-4*	WBGene00015540	177354
188971_s_at	3.44806	up	1.78579	sca-1	WBGene00004736	174747
178399_s_at	2.41910	up	1.27447	scc-3	WBGene00004738	176512
173198_s_at	2.16781	up	1.11624	sdhb-1*	WBGene00006433	179749
180256_at	2.68077	up	1.42265	sdz-24	WBGene00019495	174482
186638_s_at	2.22852	up	1.15609	sec-16	WBGene00013985	173457
185913_at	2.03995	up	1.02854	sec-23*	WBGene00004754	176289
192713_s_at	2.17381	up	1.12023	sec-24.1	WBGene00004755	180317
181627_at	2.10770	up	1.07567	sec-6	WBGene00017284	178078
193652_s_at	4.04500	up	2.01614	sec-61	WBGene00013311	173910
174940_at	2.24985	up	1.16983	SKPO-3	WBGene00017968	177324
193777_s_at	2.09274	up	1.06539	skr-1	WBGene00004807	178407
188304_at	2.28808	up	1.19413	smd-1	WBGene00004875	172775
191015_s_at	2.50954	up	1.32742	snb-1	WBGene00004897	173269
192356_s_at	2.07901	up	1.05590	spc-1	WBGene00004951	266648
**192036_at**	**4.17071**	**up**	**2.06029**	**spn-4**	**WBGene00004984**	**180603**
192674_s_at	3.62093	up	1.85636	sqt-1	WBGene00005016	179070
**188115_s_at**	**2.40859**	**up**	**1.26819**	**sqv-7***	**WBGene00005025**	**174731**
190983_at	2.14582	up	1.10153	srs-2*	WBGene00005663	174145
187924_s_at	2.08369	up	1.05914	sss-1	WBGene00006056	177859
**179485_s_at**	**2.09596**	**up**	**1.06761**	**sun-1**	**WBGene00006311**	**177843**
183970_at	2.56546	up	1.35922	T04C12.3	WBGene00011428	179802
177794_at	2.44551	up	1.29014	T05B9.1*	WBGene00011464	179532
192888_s_at	2.13107	up	1.09158	T05D4.1	WBGene00011474	174718
181494_s_at	2.14888	up	1.10359	T07C4.3	WBGene00011560	172803
189458_s_at	2.02657	up	1.01904	T07F10.1	WBGene00011587	176451
191403_s_at	2.78086	up	1.47553	T08G11.1	WBGene00011629	179763

187557_s_at	2.26625	up	1.18031	T12A2.2	WBGene00020437	172705
189733_at	2.07873	up	1.05570	T12A2.8	WBGene00020442	175886
179619_at	2.43740	up	1.28534	T12G3.5	WBGene00011740	175885
182858_at	2.08321	up	1.05881	T15B7.1	WBGene00020516	188457
181714_at	2.84927	up	1.51059	T19B10.2	WBGene00011831	188512
192370_at	2.31709	up	1.21231	T19B4.3	WBGene00020557	179551
181644_at	3.71167	up	1.89207	T21C9.13	WBGene00011898	172232
176676_s_at	2.07760	up	1.05492	T22F3.3	WBGene00020696	179480
183341_at	2.40432	up	1.26563	T23G11.1	WBGene00011968	173054
179304_at	2.59192	up	1.37402	T24F1.2	WBGene00011994	172534
188823_s_at	2.93626	up	1.55398	T25C12.3	WBGene00012018	259727
173774_s_at	2.15379	up	1.10688	T27A8.1	WBGene00013895	171921
179502_at	2.01740	up	1.01250	T28D6.3	WBGene00012123	174727
186127_at	2.37393	up	1.24728	tag-18*	WBGene00006408	176567
190568_at	2.04250	up	1.03033	tag-72	WBGene00006447	180662
189586_s_at	2.22381	up	1.15303	tbb-1*	WBGene00006536	182875
189042_at	2.09348	up	1.06590	tkt-1*	WBGene00008506	176501
185643_s_at	2.60378	up	1.38061	tnt-2	WBGene00006587	177906
176321_s_at	2.84085	up	1.50632	trap-1	WBGene00022122	181119
176353_s_at	2.99136	up	1.58080	trap-3*	WBGene00021420	171865
192151_at	2.55109	up	1.35111	tsn-1	WBGene00006626	177028
190773_s_at	2.27201	up	1.18397	tsp-12	WBGene00006638	173811
191068_at	2.09721	up	1.06847	ttr-14*	WBGene00011460	177890
190473_at	2.60745	up	1.38264	ttr-27	WBGene00011284	181272
173919_s_at	2.16214	up	1.11246	ttr-44	WBGene00008341	179769
191548_s_at	2.02966	up	1.02124	uba-1*	WBGene00006699	179835
173746_s_at	2.04287	up	1.03060	UBQL-1	WBGene00008852	172434
193282_at	2.18463	up	1.12739	uda-1	WBGene00010697	177855
**193326_s_at**	**2.61653**	**up**	**1.38765**	**ufd-2**	**WBGene00006734**	**179395**
173905_s_at	2.08181	up	1.05784	ugt-46	WBGene00015141	174295
191440_at	2.26600	up	1.18015	ugt-47	WBGene00011006	180404
193683_s_at	2.22557	up	1.15417	unc-112*	WBGene00006836	187570
172018_x_at	2.01901	up	1.01365	unc-112*	WBGene00006836	179972
190436_at	4.28773	up	2.10021	unc-15	WBGene00006754	179972
194006_at	2.00147	up	1.00106	unc-25	WBGene00006762	172491
172153_x_at	2.01902	up	1.01366	unc-44	WBGene00006780	176713
**176736_at**	**2.04168**	**up**	**1.02976**	**uri-1**	**WBGene00016944**	**177366**
193697_s_at	3.90605	up	1.96571	vha-1*	WBGene00006910	183841
189302_s_at	3.21966	up	1.68691	vha-10	WBGene00006919	176383

193564_s_at	3.29787	up	1.72153	vha-12	WBGene00006921	172216
193987_s_at	3.68330	up	1.88100	vha-13	WBGene00013025	180692
189591_s_at	2.92140	up	1.54666	vha-15	WBGene00020507	3564970
177070_s_at	2.46908	up	1.30397	vha-16	WBGene00016258	180534
187683_at	2.32334	up	1.21620	vha-17	WBGene00009882	172134
193422_s_at	3.59784	up	1.84713	vha-2*	WBGene00006911	177757
192832_s_at	2.45723	up	1.29703	vha-4	WBGene00006913	187779
173717_s_at	2.29601	up	1.19913	vha-7	WBGene00006916	174272
187561_s_at	2.91470	up	1.54335	vha-8	WBGene00006917	178219
176872_at	2.49483	up	1.31894	vit-3	WBGene00006927	177442
190596_s_at	2.05098	up	1.03632	vps-15	WBGene00014151	180647
191005_at	2.04413	up	1.03149	W02B12.12	WBGene00012207	174827
191006_s_at	2.13260	up	1.09261	W02B12.12	WBGene00012207	174755
192905_s_at	2.50124	up	1.32264	W02F12.5*	WBGene00020950	174755
181867_s_at	2.59086	up	1.37343	W03F8.4	WBGene00020994	179063
**180101_s_at**	**2.04177**	**up**	**1.02982**	**W05F2.3**	**WBGene00021035**	**177293**
191299_s_at	2.21094	up	1.14466	W07G4.4	WBGene00012338	171908
184769_at	4.05770	up	2.02066	W08E12.2	WBGene00021083	179790
172503_x_at	4.96499	up	2.31179	W08E12.3*	WBGene00021084	189291
172545_x_at	4.43934	up	2.15035	W08E12.4*	WBGene00021085	177098
178417_s_at	2.03611	up	1.02581	W09C5.7*	WBGene00012353	189292
177973_at	2.03039	up	1.02175	wago-4	WBGene00010263	174932
192784_at	2.04782	up	1.03409	wdr-5.1	WBGene00006474	171831
190863_at	2.29453	up	1.19820	xbp-1*	WBGene00006959	175474
193650_at	2.57896	up	1.36679	xbx-6	WBGene00009580	175541
176162_at	2.57633	up	1.36532	Y105E8B.5	WBGene00013690	179361
182839_at	2.09079	up	1.06405	Y11D7A.3	WBGene00012428	173323
184279_at	2.07926	up	1.05607	Y17G9B.5	WBGene00021202	177745
177160_at	2.28604	up	1.19285	Y38C1AA.7	WBGene00021398	177244
**179987_at**	**2.18934**	**up**	**1.13049**	**Y39E4B.5**	**WBGene00012716**	**176830**
191219_at	2.05307	up	1.03779	Y41C4A.11	WBGene00012757	176735
182743_at	2.61380	up	1.38615	Y47D3B.6*	WBGene00012942	189814
176471_at	2.09709	up	1.06839	Y47D7A.13	WBGene00021625	176573
175393_at	2.02450	up	1.01756	Y47D7A.13	WBGene00021625	178841
177239_s_at	2.23800	up	1.16221	Y54E10BR.5*	WBGene00021844	171876
187073_at	2.78928	up	1.47990	Y57A10A.23	WBGene00013263	178841
187345_at	2.06958	up	1.04934	Y57E12AL.1	WBGene00021956	174863
181091_at	2.73243	up	1.45019	Y69E1A.2*	WBGene00013474	179093
187891_at	2.04595	up	1.03277	Y71H10B.1	WBGene00022201	177992

175576_s_at	2.84414	up	1.50799	Y75B7AR.1*	WBGene00022287	180573
178069_s_at	2.61506	up	1.38684	ZC247.1	WBGene00013859	178630
181230_at	2.02378	up	1.01705	zip-2	WBGene00019327	172895
184102_s_at	2.67029	up	1.41700	ZK1025.2	WBGene00014182	175240
182104_at	2.62702	up	1.39343	zk1025.3	WBGene00014183	191492
182942_at	2.41681	up	1.27311	zk1058.9	WBGene00014207	175508
183237_s_at	4.77231	up	2.25469	ZK180.5	WBGene00022679	173030
173576_at	2.28066	up	1.18945	ZK180.5	WBGene00022679	177218
180664_s_at	2.10083	up	1.07096	ZK180.6	WBGene00022680	177219
179543_s_at	2.08201	up	1.05798	ZK 265.9*	WBGene00044094	177218
182638_at	2.40699	up	1.26723	ZK418.5	WBGene00022734	3565961
175796_s_at	2.56988	up	1.36170	ZK484.1	WBGene00022748	175987
179401_at	3.03787	up	1.60306	ZK662.2	WBGene00014039	172291
190938_s_at	2.06836	up	1.04848	ZK829.7*	WBGene00014096	181660
189819_s_at	2.03957	up	1.02826	ZK829.9	WBGene00014097	178132
178652_at	2.70029	up	1.43311	ZK84.1	WBGene00022649	178134
177539_at	2.88947	up	1.53080	ZK858.2	WBGene00014116	174008
193893_at	2.18448	up	1.12729	ZK909.3	WBGene00014148	191443
191572_s_at	2.70249	up	1.43429	zyg-9	WBGene00006994	191461

*target previously predicted by protein binding microarray (GROVE *et al.* 2009)

Predicted or known interaction with *daf-18*

GROVE, C. A., F. DE MASI, M. I. BARRASA, D. E. NEWBURGER, M. J. ALKEMA *et al.*, 2009 A multiparameter network reveals extensive divergence between C. elegans bHLH transcription factors. Cell **138:** 314-327.
